# Reversible electron transfer in organolanthanide chemistry

**DOI:** 10.28954/2019.csq.06.001

**Published:** 2019

**Authors:** Arnaud Jaoul, Maxime Tricoire, Jules Moutet, Marie Cordier, Carine Clavaguéra, Grégory Nocton

**Affiliations:** a LCM, CNRS, Ecole polytechnique, IP Paris, Route de Saclay, Palaiseau, France; b Laboratoire de Chimie Physique, CNRS-Université Paris-Sud, Université Paris-Saclay, 15 avenue Jean Perrin, 91405 Orsay Cedex, France

**Keywords:** Electron transfer, lanthanides, Platinum, Palladium, quantum chemistry, electron density analysis

## Abstract

This article relates the synthesis and characterization of novel heterobimetallic complexes containing a low-valent lanthanide, a tetradentate redox non-innocent ligand, *viz.* the 4,5,9,10-tetraazaphenanthrene, taphen ligand and transition metal fragments of PdMe_2_ and PtMe_2_. The experimental results are supported by a theoretical study. Investigation of their reduction properties allowed the formation of isostructural original heterotrimetallic complexes containing two Cp*_2_Yb fragments and the (taphen)MMe_2_ (M = Pd and Pt) motifs. These complexes are stable in non-coordinating solvent such as toluene but decompose in coordinating solvents such as thf. Investigation of the internal electron transfer shows that the taphen ligand behaves as a two-electrons reservoir but is capable of transferring back only one electron in thf. This reversible electron(s) transfer is rare in organolanthanide chemistry and show the potential interest of these complexes in reductive chemistry. Additionally, the trinuclear complexes feature odd X-ray crystal structures in which a deviation of symmetry is observed. The latter observation was studied in depth using quantum chemistry calculations highlighting the role of non-covalent weak interactions.

## I Introduction

Reductive chemistry with divalent lanthanides is still a modern area of research more than 40 years after the original report of Kagan exploiting the reductive properties of SmI_2_ in organic chemistry [1, 2]. The synthesis of more elaborated complexes such as substituted biscyclopentadienyl complexes [3, 4] allowed developing a very rich chemistry from small molecule activation (including N_2_) [5], reversible C-C coupling reactions [6, 7], C-H activation [8] and many other useful reactions [9]. In agreement with their accessible redox potential, Yb and Sm are the two elements that are the mostly used and recently, efficient applications have been published [10]. Other divalent complexes with non-classical lanthanides [11] have also been reported, Tm being the next one on the list of the commonly used divalent lanthanides [12]. However, their electron transfer reactivity is less explored [13, 14]. Another important information, is the electronic structure of these organometallic complexes, that is sometimes difficult to understand within simple formal Lewis structures, since Yb complexes with N-heteroaromatic cycles can form multiconfigurational ground-state wave functions and several resonant structures co-exist [15–17]. Therefore, understanding what is happening while the electron is transferred is not easy to apprehend and this necessitates the use of many spectroscopic and theoretical tools. These studies allowed us a better understanding of CO_2_ reactivity with samarocene [18,19] but also allowed rationalizing C-H activation vs. C-C coupling reaction [6,8,20], or sterically induced bipyridine reduction [14].

**Scheme 1:**
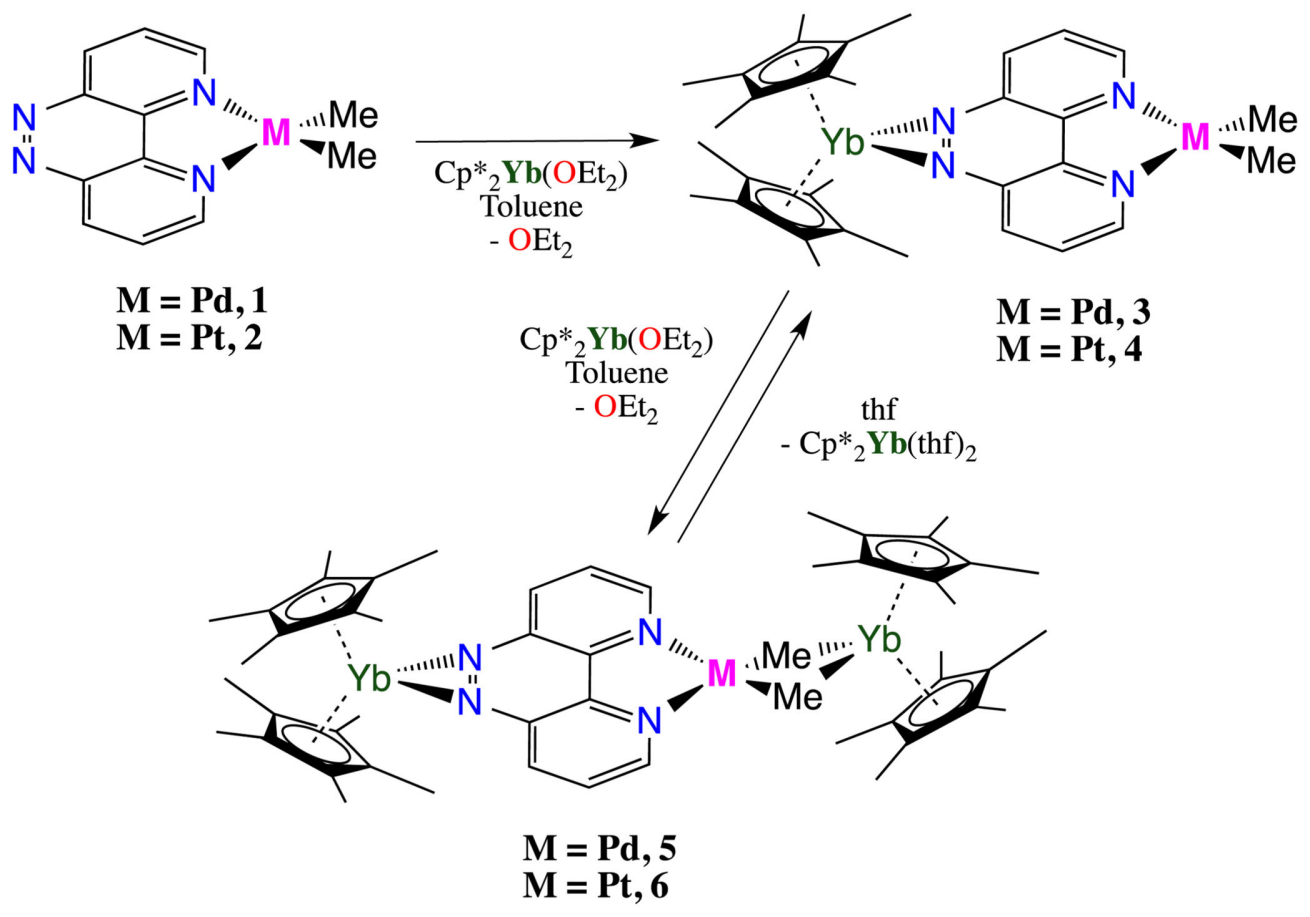
Reaction scheme for **1**-**6**.

Thereby, since N-aromatic heterocycles have also been shown to behave as useful electron reservoirs [21] when they are combined with divalent lanthanides, we have recently embarked in the synthesis of an original series of compounds, combining a reductive divalent lanthanide fragment, a redox non-innocent ligand and a reactive transition metal fragment [22]. The first step in this original approach was to build on the heterometallic complexes of Yb and Pd with two different bridging ligands, the bipyrimidine (bipym) ligand, and the 4,5,9,10-tetraazaphenanthrene, taphen ligand (see Scheme 1). The modulation of the ligands leads to the modulation of the reactivity with methyl iodine (MeI) and a relatively stable Pd^IV^ species was described upon oxidative addition with MeI [22]. After this important step, we were intrigued in knowing whether the reactivity would be also influenced by other factors, such as the number of electrons present on the ligand reservoir.

In this context, this work will present the addition of a second equivalent of the divalent ytterbium fragment, Cp*_2_Yb on **3**, leading to the formation of the heterotrimetallic complex **5**, {[Cp*_2_Yb(taphen)PdMe_2_](Cp*_2_Yb)}, in which the divalent ytterbium fragment is coordinated through the methyl groups of the Pd and in which the taphen ligand undergoes a second reduction. This unusual chemistry was also extended to Pt and has a similar outcome. In addition to the original heterometallic complexes synthesis and further characterization with theoretical methods, the key point of this work is that the second reduction occurs only in non-coordinating solvent, such as toluene, and is reversible upon addition of a coordinating solvent, such as thf. Reversible electron transfer from a redox non-innocent ligand to a reductive divalent lanthanide fragment is not usual and allows envisioning a reactivity modulation induced by the solvent, an attractive concept in organolanthanide chemistry.

## II Results and Discussion

### Experimental work. Synthesis, X-Ray diffraction, solid-state magnetism and solution NMR spectroscopy

**Figure 1:**
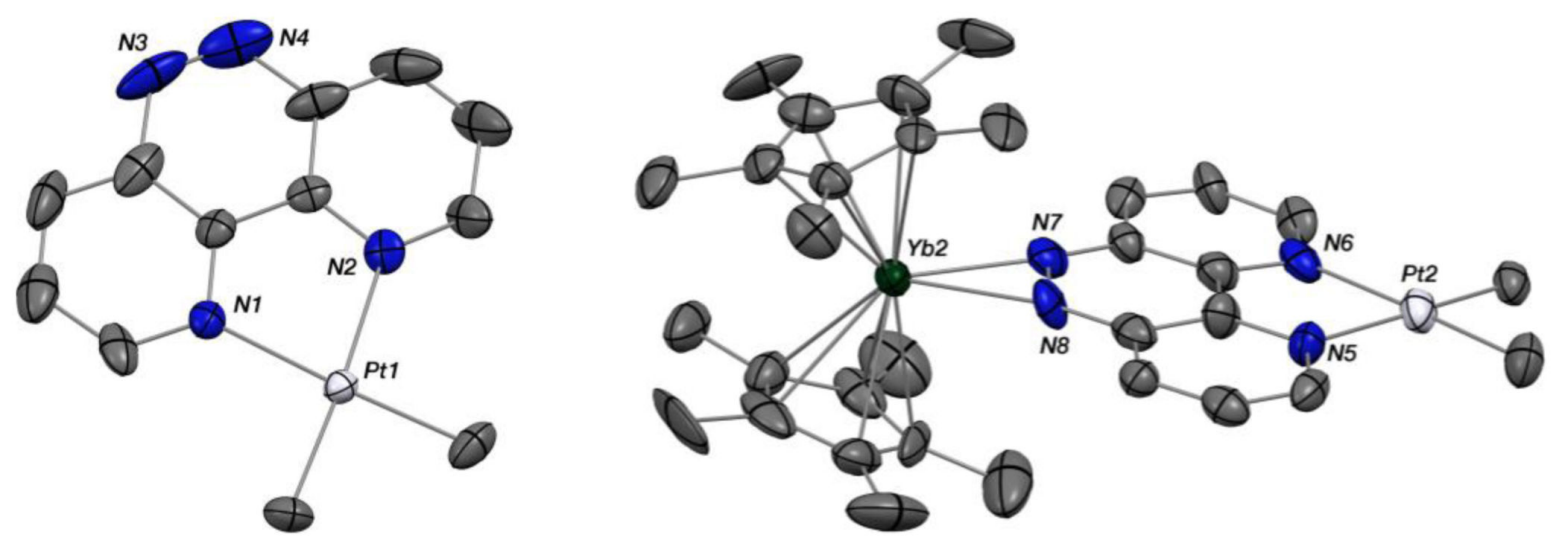
ORTEPs of the (taphen)PtMe_2_ complex **2** (left), and of Cp*_2_Yb(taphen)PtMe_2_ complex **4** (right). Ellipsoids are at 50% level. The hydrogen atoms have been removed for clarity. Atoms in grey are carbon, nitrogen atoms are in blue, ytterbium in dark-green and Pt in off-white.

The 4,5,9,10-tetraazaphenanthrene, taphen ligand, has been synthetized according to published procedure [23]. X-ray suitable yellow crystals have been obtained by crystallization in toluene. ORTEP and main metrics are available in SI and in Table S8 (see Supporting Information, SI). The synthesis of **1** and **3** were reported in a previous work [20]. A similar synthetic procedure has been used for the preparation of **2** and **4**. In toluene, the red suspension of **2** is converted to **4** by addition of one equivalent of Cp*_2_Yb(OEt_2_), leading to a brown solution, from which X-ray suitable brown crystals of **4** can be crystalized at -35 °C. X-ray suitable crystals of **2** can be grown from dilute CH_2_Cl_2_ solution at -35°C. Main distances are given in Table 1, while an ORTEP representation of the structure is given in Figure 1. The ^1^H NMR in solution of crystals of **4** is shown in Figure S2 and that of the precursor **1** in Figure S1.

The solution symmetry is C_2v_ with one signal for the Cp* resonance at 13.4 ppm, three signals for the taphen ligand at 72.7, -138.4 and 290.8 ppm, and one signal for the methyl resonance of the PtMe_2_ fragment. The chemical shifts are characteristic of the paramagnetism of the solution and indicate that the ytterbium metal center is oxidized at its trivalent state (f^13^), while an electron has been transferred to the (taphen)PtMe_2_ fragment. The ^1^H NMR is very close to that reported for the Pd analogue [22], which indicates that the electronic structure is likely to be similar. Additionally, the solid-state magnetic measurements of **4** (Figure S11) is clearly a triplet as shown for **3**. Moreover, similarly to **3**, the VT NMR chemical shift vs. 1/T plot (Figure S6) shows that **4** follows the Curie law over the studied temperature regime.

When a second equivalent of Cp*_2_Yb(OEt_2_) was added to **4** in toluene, the color of the solution turned to deep purple within minutes at room temperature. An *in situ* ^1^H NMR made after 15 min (Figures S4 and S5) indicated the presence of a new paramagnetic species **6**, in solution. The first key point is the presence of two signals for the Cp* resonances while the taphen signals were still three, at 180.7, 71.8 and 21.4 ppm, in agreement with a plane of symmetry (symmetry C_s_) or with a C_2_ axis (Symmetry C_2v_). Since one Cp* signal lied under the residual peak of the solvent, a ^1^H NMR spectra at 60 °C confirmed the presence of two distinct resonances. Another interesting point in these ^1^H NMR spectra is the absence of the methyl resonances of the PtMe_2_ fragment. The same experiment was performed with **3** and one more equivalent of Cp*_2_Yb(OEt_2_) in toluene, which led to a very similar outcome, although the *in situ* NMR is less clean since many other resonances appear rapidly (Figure S3). However, the close resemblance of the two ^1^H NMR spectra envisioned similar molecules. VT NMR of the *in situ* solution of **6** (Figure S8) also confirmed that the species follows the Curie law in the studied temperature range; this can also be observed with **5** (Figure S7). This situation accounts for the formation of a novel paramagnetic species, but does not inform neither on its structural nature, nor its electronic structure.

**Figure 2:**
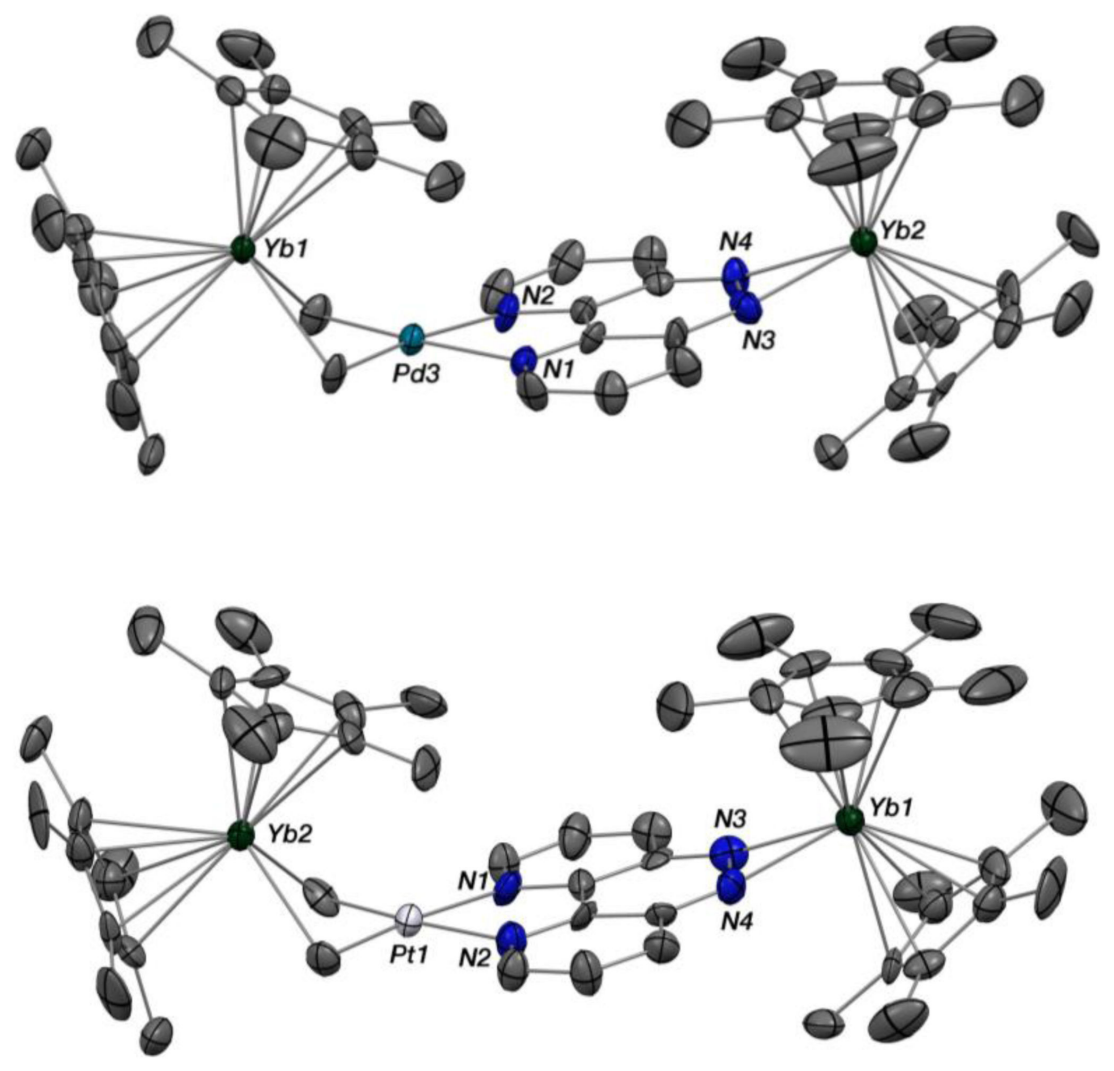
ORTEPs of the {[Cp*_2_Yb(taphen)PdMe_2_](Cp*_2_Yb)} complex **5** (top), and of the {[Cp*_2_Yb(taphen)PtMe_2_](Cp*_2_Yb)} complex **6** (bottom). Ellipsoids are at 50% level. The hydrogen atoms have been removed for clarity. Atoms in grey are carbon, nitrogen atoms are in blue, ytterbium in dark green, Pd in light green, and Pt in off-white.

Both purple solutions were left standing at -35°C overnight and produced deep purple crystals. ORTEP representation of **5** and **6** are shown in Figure 2 and main distances are given in Table 1. Both structures are essentially isostructural and feature the former Cp*_2_Yb(taphen)MMe_2_ fragments (M = Pd, **3**; M = Pt, **4**) with an additional Cp*_2_Yb complex coordinated through the methyl of the MMe_2_ moieties. This original structure is accompanied with an odd bending of the latter Cp*_2_Yb fragment, closing the gap between the Yb metal center and the transition metal, a point that needs to be clarified. However, care is needed since this structure does not correspond to the symmetric solution structure observed in NMR, even at lower temperature. Therefore, the solution structure and solid-state structure do not agree. Another interesting point resides in the electronic structure of such a complex. In the Cp*_2_Yb(taphen)MMe_2_ complexes (M = Pd, **3**; M = Pt, **4**), the metal is oxidized and the (taphen)MMe_2_ is reduced. However, in **5** and **6**, two electrons are available from the two divalent ytterbium centers and their localization is unclear. At this stage, many resonant structures may be written to form an overall paramagnetic structure.

Since we have access to the metric parameters of the free ligand and to the different complexes, in which the taphen ligand is involved, it is possible to directly compare these useful data. The metric paramaters of the taphen ligand and of complexes **1**-**6** are presented in Table 1. The δ column represents the metric difference between the previous column and the metric of the free ligand, so that for each complex, a deviation of the metric from the free ligand is given. No or little deviation is not highlighted; a moderate one is shown in orange and a larger one in red. The Cp*-M distances are also reported as well as the M-L coordination distances. **1** and **2** have similar M-C distances, while M-N distances are smaller for **2** (2.106(8) Å vs 2.153(4) Å in **1**). In the bimetallic complexes **3** and **4**, these distances are only slightly modified compared to the Pd and Pt complexes **1** and **2** [22]. In the trimetallic complexes **5** and **6**, these are also only slightly modified, although an increase of the M-C bond distances of 0.04 Å and 0.05 Å is noted for the Pd and Pt compounds, respectively, which arises from the ytterbium coordination. The differences between the Yb-Cp* and Yb-N(C) for the dinuclear complexes **3** and **4** and the trinuclear complexes **5** and **6** are more interesting: a small elongation is observed for the Yb-Cp* distances, while the Yb-N distance decreases strongly from 0.08 and 0.07 Å for the Pd and Pt compounds, respectively. This decrease is due to a charge modification on the ligand. In the event of a possible second reduction of the taphen ligand, the charge would be dianionic and the Yb-N distance would appear smaller. The Cp*-Yb distance of the ytterbium center coordinated to the methyl moieties is a little larger than that of the other by about 0.05 Å, but yet in the range found for trivalent ytterbium complexes [20], and therefore agrees with a second reduction of the taphen ligand. The longer distances are well explained by increased steric at this side because of the bending of the Cp*Yb fragment over the Pd/PtMe_2_ fragment. Entry O in Table 1 indicates very strong δ modifications for the N=N bond in the taphen ligand. This increase also fits with the presence of more electronic density on this bond, in agreement with a taphen^2-^ ligand. The other distances in the ligand are also significantly modified indicating a strong modification of the ligand electronics. As this stage, and from the X-ray analysis, a possible electronic picture for **5** and **6** is given by the presence of two trivalent ytterbium atoms, a divalent group-10 metal center, and a doubly reduced taphen ligand, taphen^2-^.

The magnetic data have already been reported for **3** [22]. The data were in agreement with the presence of a triplet at all temperature, *viz.* a trivalent ytterbium and a radical located on the taphen ligand. Since **4** possesses a very similar magnetic behavior in NMR, the electronic structure is likely to be similar. However, temperature dependent magnetic data were recorded for **5** (Figure S12), in which a second ytterbium is coordinated. The χT value is 4.78 cm^3^.K.mol^-1^ at room temperature, in agreement with the presence of two trivalent ytterbium centers (^2^F_7/2_) for which the theoretical value is 5.08 cm^3^emu.K.mol^-1^. The value decreases when decreasing the temperature to reach 3.21 cm^3^.K.mol^-1^. This behavior is typical of lanthanide magnetism and is due to the depopulation of the crystal-field state. No sign of any coupling is present. Therefore, the magnetism is a strong evidence for the presence of two trivalent ytterbium centers and a *diamagnetic* transition-metal fragment, *i.e.* the taphen ligand is doubly reduced and is dianionic and *diamagnetic*.

**Scheme 3:**
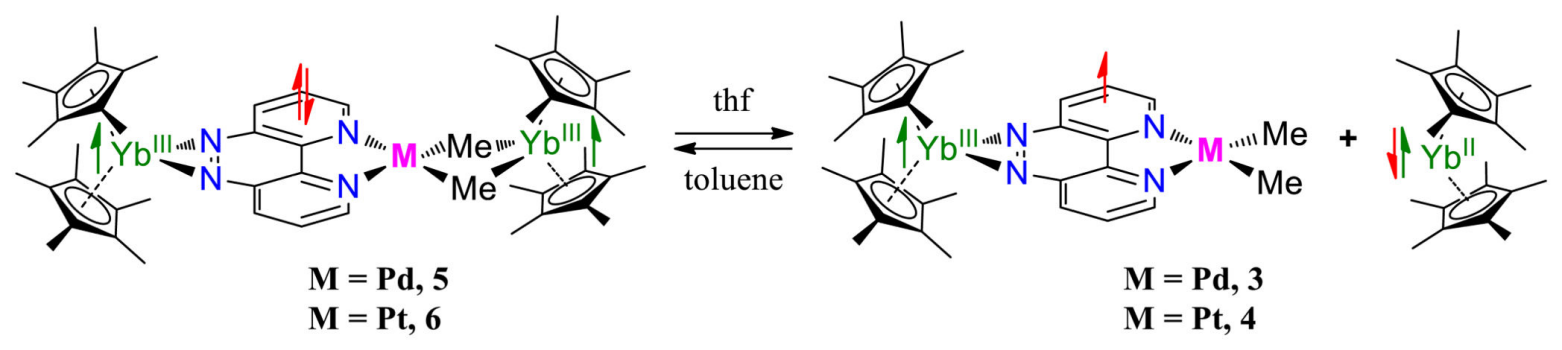
Reversible electron-transfer processes from for **3**-**6**.

Similarly to what was done in a previous work [22], reactivity with MeI was attempted. However, the resulting ^1^H NMR after the reaction of 1 equivalent of MeI shows a mixture of compounds, mostly the complexes **4** and **6**, with **4** in a higher amount, indicating that the trinuclear structure has been disrupted (see Figure S9). It is likely that the weak coordination of the Cp*_2_Yb fragment to the methyl group does not compete with a favorable coordination of MeI. However, and interestingly, since **4** is the major species, the second electron that was stored on the taphen ligand has been removed and is used to cleave MeI. This is particularly interesting since it means that a reversible electron transfer has occurred. Such reactivity is not unique but is rare in organolanthanides [10]. It mostly shows that electron(s) can be selectively stored on ligand centers and re-used for further reactivity at an oxidized metal center. However, in this particular example, the reactivity is difficult to apprehend since many species are formed and the fate of MeI is not well understood. Notably, there is an eventuality of an outer-sphere electron transfer from the taphen ligand to explain this observation; the ytterbium metal center may not participate. In order to better assign this reversible electron transfer, we chose to look at the solvent nature and selected a strongly coordinating solvents, such as thf (Scheme 3).

Dissolution of **6** in thf leads to the clean formation of **4** and Cp*_2_Yb(thf)_x_ (Figure S10) [3]. Once again, it shows that the stored electron can be re-used but, this time, the electron goes back to the ytterbium metal center with the coordination of thf: the ytterbium metal center is formally reduced. Thus, it seems that the coordination of the ytterbium to the methyl groups helps to reduce the taphen ligand with a second electron. Therefore, the coordination is the key point for the electron transfer and the de-coordination leads to the electron-back transfer. This feature is really interesting in organolanthanides and is closer to what is usually observed in transition-metal chemistry. It validates the concept that the stored electrons in redox non-innocent ligands can be back-transferred upon coordination-chemistry engineering. Additionally, the solution containing **4** and Cp*_2_Yb(thf)_x_ can be exposed to reduced pressure for 12 h to removethe thf molecules. The dissolution of the residue in toluene-d_8_ recovers the typical ^1^H spectrum of **6**; the process is therefore reversible.

### Theoretical work. Ligand description, DFT, electron density analysis

**Figure 3:**
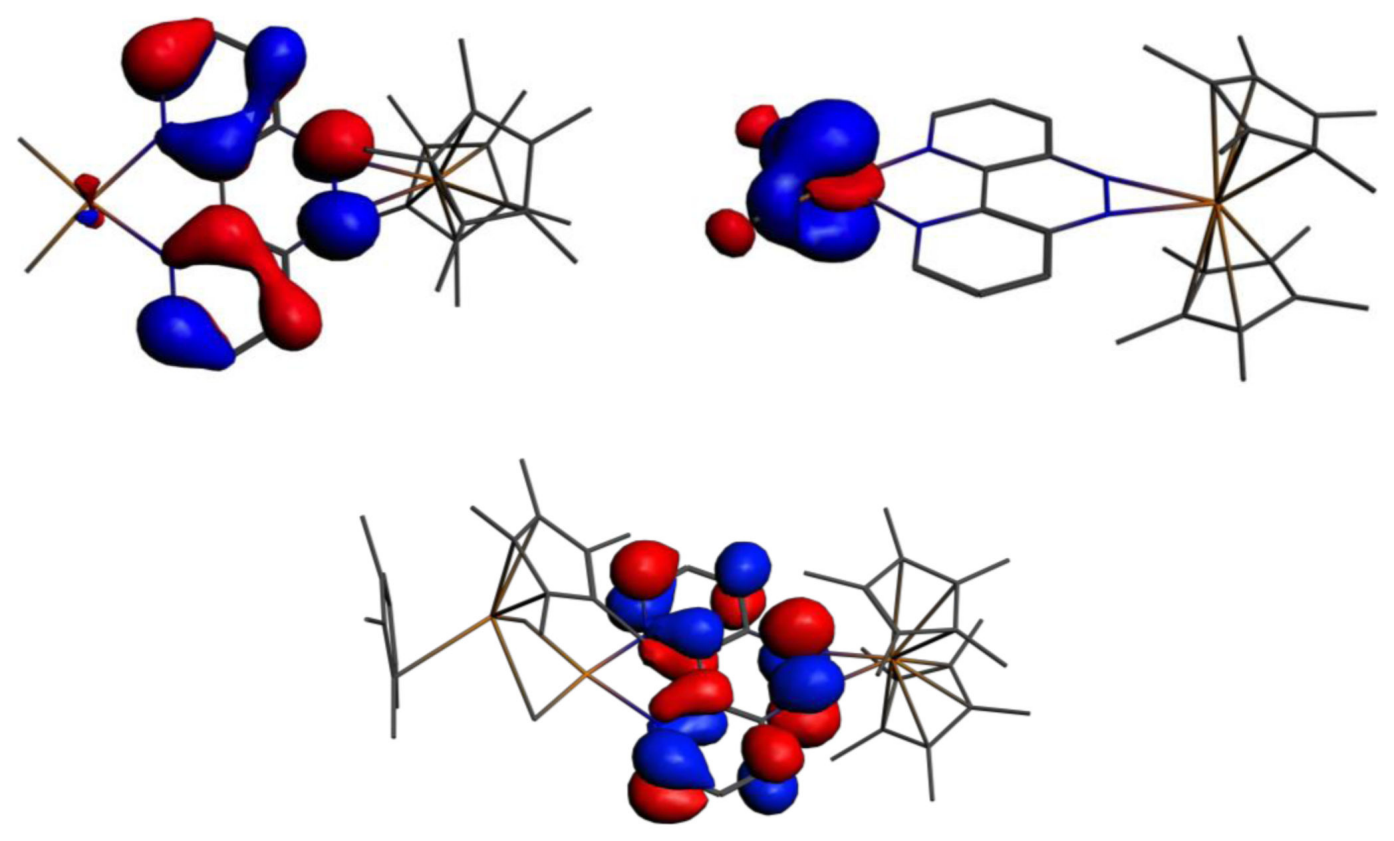
The two highest molecular orbitals for Cp*_2_Yb(taphen)PdMe_2_ **3** (top). HOMO of {[Cp*_2_Yb(taphen)PdMe_2_](Cp*_2_Yb)} **5** (bottom).

The geometry of the taphen ligand, as well as the geometries of the palladium complexes **3** and **5** and the platinum complexes **4** and **6**, were optimized at the DFT/PBE-D3(BJ) level. The electronic structure of the singlet dianion taphen ligand was also computed at the CASSCF(8,7) level of theory (larger active spaces provide similar results). At this level, the molecule presents a ground state electronic structure that is represented as π_1_*^2^ (Figure S14), similar to the DFT results. The electron density analysis of **3** and **5** is described below; moreover, similar results have been obtained for **4** and **6** (see SI). Figure 3 represents the two highest Kohn-Sham orbitals of **3** and **5** at the DFT/PBE0-D3 level (those for complexes **4** and **6** are described in the Figures S15 and S16). The lowest energy of **3** was measured to be the triplet state compared to the singlet state. The optimized geometry for the triplet state agrees with the experimental one. The SOMO is clearly located on the taphen ligand and, interestingly, the energy of the dz^2^ orbital located on the Pd remains high as suggested by the reactivity with MeI, which reacts preferably with the Pd center and not with the lanthanide center. For **5**, three spin states, *i.e.* singlet, triplet and quintet, were computed at the PBE0-D3 level, resulting to a lower energy for the triplet, in agreement with the SQUID measurements as well (Figure S12). Two single electrons reside on each Yb metal centers and the taphen ligand has a doubly occupied π* orbital (Figure 3, bottom). Interestingly, the dz^2^ orbital of the Pd is lower in energy compared to **3**. The computed geometry of **5** agrees well with the XRD experimental one, including the bent of the second Cp*_2_Yb fragment over the Pd metal center. It is important to note at this stage that the use of a density functional that includes dispersion corrections is essential in order to reproduce the solid-state experimental data. On the other hand, the ^1^H NMR shows a symmetrical complex even at low temperature and is not indicative of any specific interaction between the Cp*_2_Yb fragment and Pd metal center in solution. This dichotomy will be discussed in the following sections.

An energy decomposition analysis was performed to quantify the interaction strength between the ytterbium center and the methyl groups of the PdMe_2_ fragment in **5**. The molecule was divided into two fragments: one Cp*_2_Yb fragment and one Cp*_2_Yb(taphen)PdMe_2_ fragment. The energy results are presented in Table 2.

The total bond strength between the lanthanide and the methyl groups is weaker than between the lanthanide center and the taphen moiety: in the present case, the bond strength is 35 kcal.mol^-1^ while it is 60 kcal.mol^-1^ between Cp*^2^Yb and (taphen)PdMe_2_. The bonding interaction is spread over electrostatic (51%) and orbital interactions (39%) with a small influence of the dispersion effects (5%), as observed in **3** (Table S1). The orbital interaction is strong between the lanthanide and the methyl group and accounts for 39% of the attractive bonding interaction. The bent angle observed experimentally for this complex is strongly related to the bonding interaction that occurs between the lanthanide and the methyl groups.

Compared to Cp*_2_Yb(thf)_2_, the bond strength between the lanthanide and the methyl groups in **5** is almost similar (35 vs 31 kcal.mol^-1^). However, the repartition of the bonding interaction is different. For Cp*_2_Yb(thf)_2_, the dispersion forces account for 20% of the bonding, the orbital interaction for 26% and the electrostatic interaction for 55%. While this percentage for the electrostatic interaction is similar for the two complexes, the dispersion forces play a more important role in the interaction of Cp*_2_Yb(thf)_2_. The similar value for the bond strength can explain why experimentally at room temperature in thf, drops of thf compete with the Yb⋯Me_2_Pd interaction.

**Figure 4:**
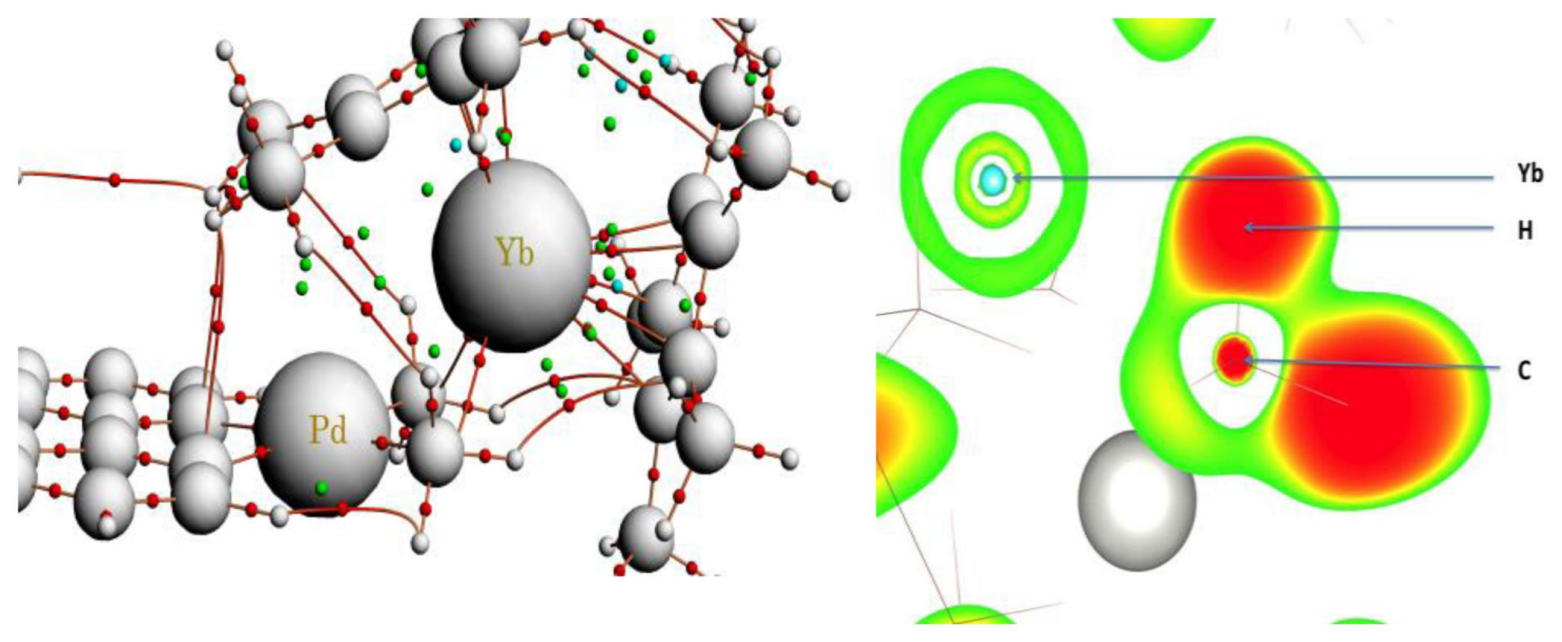
QTAIM and EFL analyses of the coordination of the second Cp*2Yb fragment in {[Cp*2Yb(taphen)PdMe2](Cp*2Yb)} **5**.

QTAIM and ELF calculations were performed to complement the analysis of the bonding structure of both the dinuclear and the trinuclear complexes **3** and **5**, respectively. QTAIM results show two bond critical points (BCP) between the ytterbium center and the two methyl groups (Figure 4, left). The interaction between the palladium and its ligands is different in **5** compared to **3**. In **3**, the Pd-N and Pd-C bonding are slightly more ionic, with Laplacian values larger than in **5** (from 0.36 to 0.34 for Pd-N, in **3** and **5**, respectively, and from 0.17 to 0.14 for Pd-C, in **3** and **5**, respectively). The BCP located between the methyl groups and the ytterbium center has a positive Laplacian value (0.111) and a small density (0.028): this bonding can be considered as weak and electrostatic.

On the contrary to the QTAIM calculation, no ELF valence basin is found between Yb and C. Instead, valence basins V(Yb,H) located close to the lanthanide (at 1.45 Å) and shared with the four closest hydrogen atoms are present (see Figure 4, right). The total density for these basins is 7.6 electrons, which means 3.8 electrons per methyl group. This value can be compared with that found for the basins between the lanthanide and the carbon atoms of the Cp* rings, V(Yb,C(Cp*)): these basins are located at 1.44 Å from the lanthanide and the total density for the two Cp* rings of Yb1 is 5.8 electrons. Hence, there is more electron shared with the two methyl groups than with the two Cp*. However, the deformation of the valence basins is small, corresponding to an electrostatic interaction. As a result, the interaction with the two methyl groups can be correlated to the presence of valence basins between the lanthanide and the hydrogen atoms and the presence of a BCP between the lanthanide and the carbon atoms.

**Figure 5:**
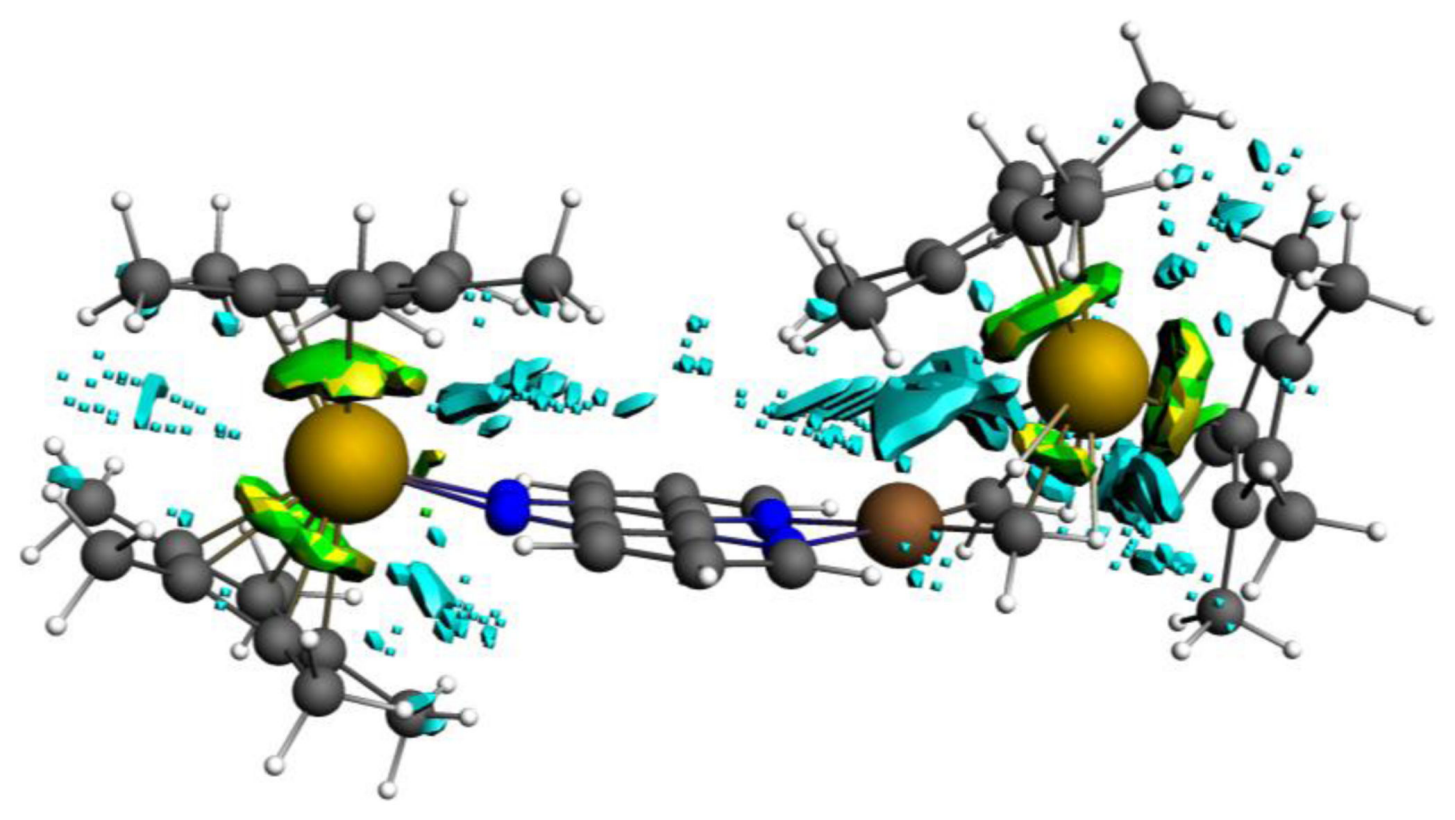
NCI plot: blue, yellow and green surfaces represent weak van der Waals interactions, isosurface of 0.03 for{[Cp*_2_Yb(taphen)PdMe_2_](Cp*_2_Yb)} **5.**

Additionally, dispersion effects are keeping the cohesion of this structure. The geometry optimization without the Grimme’s dispersion corrections (D3) leads to a linear structure. The plot of the non-covalent interactions (NCI) highlights a large amount of van der Waals forces between Pd and Yb (Figure 5). We can conclude that dispersion forces are in part responsible for the interaction between the two metals.

Finally, the analysis of the molecular orbitals of the complex does not show any bonding interaction between the methyl groups and the ytterbium ion. However, taking a closer look at the molecular orbitals, one interaction was found to possibly explain this structure: there is a small interaction between the π orbitals of one Cp* ring and the 4d_z2_ orbital of the palladium center (Figure S17). This type of orbital overlap is not supposed to be favorable as it is a four-electrons-in-two-orbitals interaction. However, the Pd 4d_z2_ orbital energy decreases compared to **3**, going from the HOMO to a deeper orbital in **5**. Hence, this is enhancing the stability of the compound.

## III Conclusions

Heterometallic complexes containing two low-valent lanthanide metal centers and group-10 transition metals (Pd and Pt) have been synthesized, characterized and analyzed computationaly. The bimetallic complexes Cp*_2_Yb(taphen)PdMe_2_ **3** and Cp*_2_Yb(taphen)PtMe_2_ **4** are paramagnetic and one single electron is located on the taphen ligand. When one more equivalent of the low-valent lanthanide Cp*_2_Yb fragment is added, trinuclear species form in which two Cp*_2_Yb fragments surround the (taphen)MMe_2_ (M = Pd and Pt) complex as confirmed by XRD data. Variable temperature^1^H NMR, the solid-state magnetism as well as the XRD data analysis all indicate that the taphen ligand is doubly reduced and both ytterbium centers are oxidized. Quantum chemistry calculations at the DFT level confirmed these experimental evidences. The rather odd bending observed in the solid-state (XDR) but not in solution (^1^H NMR) was investigated deeply with electronic-density tools, named QTAIM and ELF, as well as with NCI analysis. The conclusion is that this behavior comes from weak – and mostly electrostatic– interactions in the solid-state. When the heterometallic trimers are exposed to thf, the dimers are re-formed, implying that one electron is going back to one of the ytterbium fragments. This process is reversible and therefore highlights reversible electron transfers in organolanthanide chemistry playing with the lanthanide-ligand interaction. Two important take-home messages from this work are: i) the solid-state XRD data should be treated cautiously since packing forces may lead to over-interpretations in the overall bonding studies in organolanthanides complexes; ii) reversible electron transfers can be taken into account for the development of reductive reactivity with compounds containing low-valent lanthanides ions. Looking for more redox-switchable reactivity processes upon coordination and/or solvation is the next step to this work.

## IV Materials and Methods

All reactions were performed using standard Schlenk-line techniques or in a drybox (MBraun). All glassware was dried at 120 °C for at least 12 h prior to use. Toluene and thf were dried over sodium, degassed and transferred under reduced pressure in a cold flask. Toluene-d_8_ was dried over sodium while thf-d_8_ was dried and stored over molecular sieves. Elemental analyses were obtained from Mikroanalytisches Labor Pascher. ^1^H NMR spectra were recorded on Bruker Avance II or III-300 MHz spectrometers with J. Young valve NMR tubes. ^1^H chemical shifts are expressed relative to TMS in ppm. Magnetic susceptibility measurements were made for all samples on powder in sealed quartz tubes at 0.5 and 20 kOe in a 7 T Cryogenic SX600 SQUID magnetometer. Diamagnetic corrections were made using Pascal’s constants. The 4,5,9,10-tetraazaphenanthrene (taphen) ligand [23], the (SMe_2_)_2_Pt_2_Me_4_ [24], Cp*_2_Yb(OEt_2_) [25] and Cp*_2_Yb(taphen)PdMe_2_ (**3**) [22] complexes were synthetized according to published procedures. The X-band EPR spectrum for **3** was recorded on a Bruker ELEXSYS 500 spectrometer equipped with a Bruker ER4119HS X band resonator, an Oxford Instrument continuous flow ESR 900 cryostat, and a temperature control system. The sample was prepared in a sealed quartz 4 mm tube and was recorded under non-saturating conditions.

### Crystal structures

Single crystals of the taphen ligand as well as **2**, **4**, **5**, and **6** were mounted on a Kapton loop using a Paratone N oil on a Nonius diffractometer equipped with a APEX II CCD BRUKER detector and a graphite Mo-Kα monochromator were used for the data acquisition. All measurements were done at 150 K and a refinement method was used for solving the structure. The resolution of the solid-state structure was accomplished using the SHELXS-97 [26] and SHELXT [27] program. The refinement was performed with the SHELXL [28] program and the structure solution and the refinement were achieved with the PLATON software [29]. ORTEP representations are obtained with the MERCURY software. All atoms – except hydrogens – were refined anisotropically. The position of the hydrogen atoms was determined using residual electronic densities, which are calculated by a Fourier difference. A final weighting step, followed by multiples loops of refinement, was performed. The crystal structures of **1**-**3** have been deposited in the CCDC with # 1906955–1906959 for taphen, **2**, **4**, **5**, and **6**, respectively.

### Quantum chemistry calculations

Geometry optimizations of the taphen ligand and the various complexes were performed at the DFT/PBE-D3(BJ) level associated to all-electron Gaussian SVP basis set and using the ORCA software [30, 31]. The ZORA Hamiltonian [32] implemented in ORCA was employed to take into account relativistic effects. Single-point energy calculations were performed at the PBE0-D3/TZVP level. Complete active space self-consistent field (CASSCF) calculations were performed on the taphen ligand using ANO-RCC-VTZP basis sets. The MOLCAS 8.0 program package was used [33]. The Cholesky decomposition framework [34] was also used to accelerate the calculation of the two-electron integrals. The ADF program package [35] was used for molecular orbital and energy decomposition analyses and bonding analyses by the Quantum Theory of Atoms In Molecules (QTAIM) [36] and Non-Covalent Interactions (NCI) [37]. The DFT/PBE0-D3 level was used with the ZORA Hamiltonian and all-electron TZP basis set. Further calculations were performed to compute the Electron Localisation Function (ELF) with the DGrid 4.6 program [38, 39].

### Syntheses

(taphen)PtMe_2_, **2**. The reaction of (SMe_2_)_2_Pt_2_Me_4_ (114 mg, 0.21 mmol) with the ligand taphen (74.9 mg, 0.420 mmol) was performed in thf at room temperature. The yellow suspension became darker and darker over several hours upon stirring. After 12 h, the dark red suspension was let stand at room temperature, centrifuged and filtered. The dark red solid was washed with minimal amount of CH_2_Cl_2_ and dried under reduced pressure. The complex is obtained in good yield as dark red powder (147 mg, 0.365 mmol, 87 %). The crystals were obtained from a cold (-35 °C) saturated CH_2_Cl_2_ solution. ^1^H NMR (taphen)PtMe_2_ **2**, (δ ppm, dmso-d_6_, 293 K): 9.67 (s, br, 2H), 9.63 (s, br, 2H), 8.39 (t, *^3^J_PtH_* = 7 Hz, 2H), 1.08 (t, *^2^J_PtH_* = 44 Hz).

Cp*_2_Yb(taphen)PtMe_2_, **4**. 35.8 mg (0.088 mmol) of (taphen)PtMe_2_, **2,** were combined with 45.9 mg of Cp*_2_YbOEt_2_ (0.088 mmol) in 2 mL of toluene. The solution turned dark brown immediately along with dissolution of the powders. The reaction was stirred for 3 h at room temperature, filtered and cooled at -35 °C to yield dark brown X-ray suitable crystals of **4** in moderate yield (38.9 mg, 0.046 mmol, 53 %). ^1^H NMR (δ ppm, toluene-d_8_, 293 K): 72.7 (s, 2H, bipym), 13.4 (s, 30 H, Cp*), -13.2 (s, 6H, Me), -138.4 (s, 2H, bipym), -290.8 (s, 2H, bipym). Elemental analysis for C_32_H_42_N_4_PtYb•1.20Toluene, calcd. C, 49.56; H, 5.83; 4.19, found C, 49.44; H, 5.70; 4.25.

{[Cp*_2_Yb(taphen)PdMe_2_](Cp*_2_Yb)}, **5.** 21.2 mg (0.0675 mmol) of (taphen)PdMe_2_, **1**, were combined with 69.8 mg of Cp*_2_YbOEt_2_ (0.1026 mmol) in 4 mL of toluene. The reaction was stirred for 12 h at room temperature and turned dark purple after a short dark brown stage. After this, it was filtered and cooled at -35 °C to yield dark purple X-ray suitable crystals of **5** in low yield (25.4 mg, 0.0212 mmol, 31 %). ^1^H NMR (δ ppm, toluene-d_8_, 293 K): 260.8 (s, 2H, bipym), 82.81 (s, 2H, bipym), 22.58 (s, 2H, bipym), 7.94 (s, 15H, Cp*), 4.20 (s, 15H, Cp*). The obtained crystals are not stable above 0 °C and elemental analysis could not be obtained for **5**.

{[Cp*_2_Yb(taphen)PtMe_2_](Cp*_2_Yb)}, **6.** 19.8 mg (0.0491 mmol) of (taphen)PtMe_2_, **2**, were combined with 50.8 mg of Cp*_2_YbOEt_2_ (0.982 mmol) in 2 mL of toluene. The reaction was stirred for 12 h at room temperature and turned dark purple after a short dark brown stage. The solution was filtered and cooled at -35 °C to yield dark-purple X-ray suitable crystals of **6** in good yield (50.9 mg, 0.0395 mmol, 80 %). ^1^H NMR (δ ppm, toluene-d_8_, 293 K): 250.4 (s, 2H, bipym), 83.2 (s, 2H, bipym), 20.9 (s, 2H, bipym), 6.98, (s, 30 H, Cp*), 5.44 (s, 30 H, Cp*). Elemental analysis for C_52_H_72_N_4_PtYb_2_•0.75Toluene, calcd. C, 49.22; H, 5.51; 5.88, found C, 49.17; H, 5.34; 5.94.

### Reactivity

The reaction of MeI on **6** was followed in toluene-d_8_ in a J. Young valve NMR tube. An aliquot of 2 equivalents of thf in a toluene-d_8_ solution was added on a toluene-d_8_ solution of **6**. The thf can be removed under reduced pressure for 12 h and the dissolution of the residue in toluene-d_8_ recovers the ^1^H NMR spectrum of **6**.

## Supplementary Material

Supporting information

## Figures and Tables

**Scheme 2:**
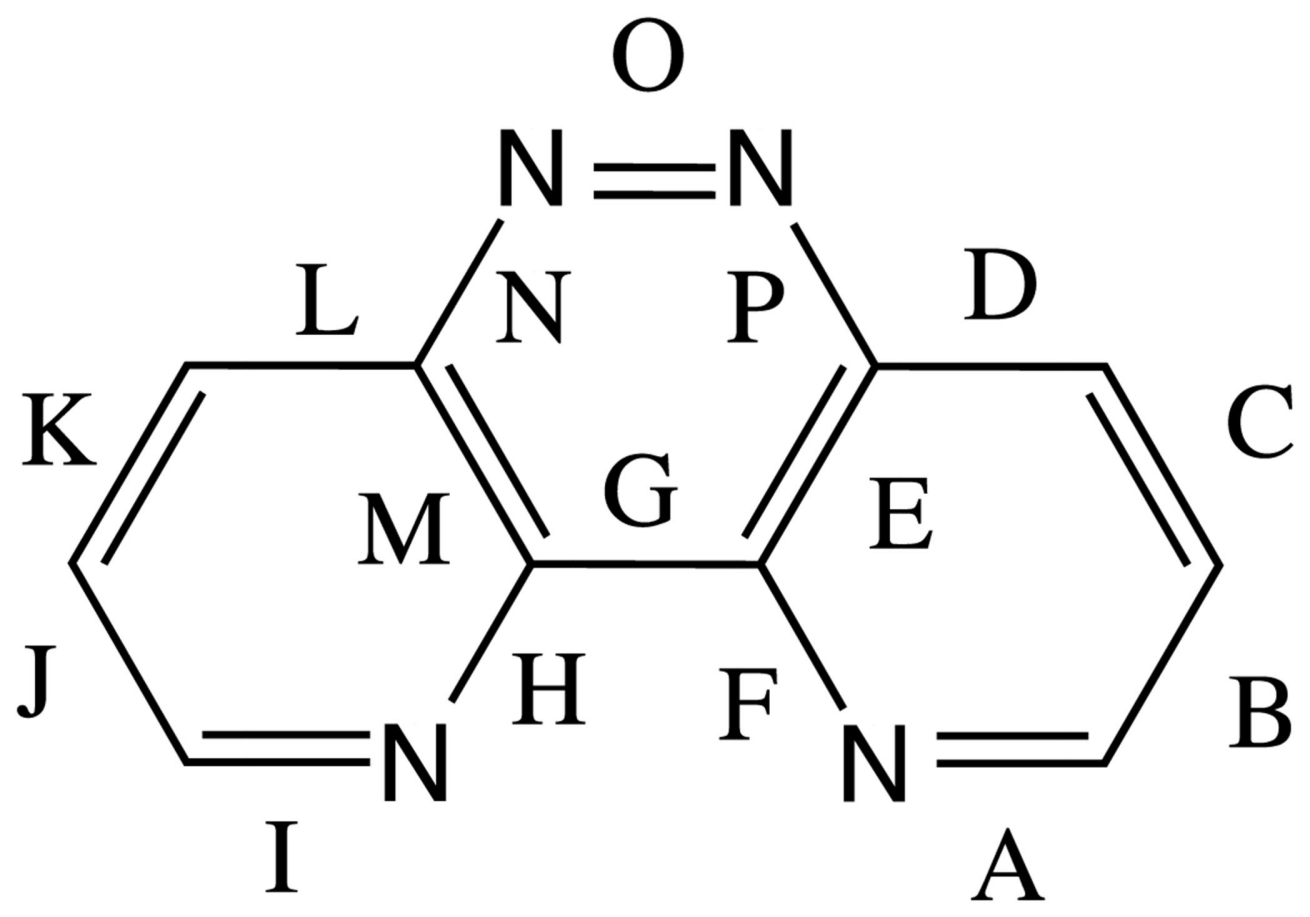
Bond labeling for the taphen ligand

**Table 1:** Metric parameters gathered from X-ray diffraction at 150 °C for the free ligand and complexes 1 to 6.

Bond labels for taphen (L) (Scheme 2)	Taphen (L)	1	δ	2	δ	3	δ	4	δ	5	δ	6	δ
A	1.324(2)	1.333(1)	+0.01	1.35(1)	+0.03	1.33(1)	+0.01	1.38(2)	**+0.06**	1.37(1)	**+0.05**	1.37(1)	**+0.05**
B	1.406(6)	1.394(2)	-0.01	1.39(1)	-0.01	1.40(1)	0	1.39(1)	-0.01	1.36(1)	-0.04	1.39(2)	-0.01
C	1.366(5)	1.37(1)	0	1.37(2)	0	1.36(1)	0	1.36(2)	0	1.39(1)	+0.03	1.38(2)	+0.02
D	1.409(2)	1.406(1)	0	1.40(1)	0	1.43(1)	+0.02	1.41(1)	0	1.41(1)	0	1.44(2)	+0.03
E	1.403(2)	1.395(2)	0	1.38(1)	-0.02	1.41(1)	+0.01	1.42(1)	+0.02	1.42(1)	+0.02	1.41(2)	+0.01
F	1.359(3)	1.363(2)1.	0	1.36(1)	0	1.35(1)	0	1.36(1)	0	1.33(1)	-0.02	1.34(1)	-0.02
G	1.443(4)	418(4)	-0.02	1.42(1)	-0.02	1.42(1)	-0.02	1.44(2)	0	1.45(1)	+0.01	1.47(2)	+0.03
H	1.349(3)	1.321(2)	-0.02	1.36(1)	+0.01	1.34(1)	0	1.36(2)	+0.01	1.34(1)	0	1.33(1)	-0.02
I	1.326(2)	1.399(6)	0	1.32(1)	0	1.33(1)	0	1.35(4)	+0.03	1.34(1)	+0.02	1.37(2)	**+0.05**
J	1.407(2)	1.408(1)	0	1.41(1)	0	1.41(1)	0	1.38(2)	-0.02	1.38(1)	+0.02	1.38(2)	-0.02
K	1.365(2)	1.370(6)	0	1.35(1)	-0.01	1.35(1)	-0.01	1.40(1)	+0.04	1.37(1)	+0.04	1.38(2)	+0.02
L	1.413(2)	1.392(1)	-0.02	1.39(1)	-0.02	1.42(1)	+0.01	1.40(3)	-0.01	1.44(1)	+0.03	1.41(2)	0
M	1.412(2)	1.363(2)	**-0.05**	1.38(1)	-0.03	1.42(2)	+0.01	1.40(1)	-0.01	1.42(1)	+0.01	1.40(2)	-0.01
N	1.381(1)	1.387(3)	0	1.40(1)	+0.02	1.38(1)	-0	1.35(3)	-0.03	1.35(1)	-0.03	1.34(1)	-0.04
O	1.293(1)	1.299(5)	0	1.30(1)	0	1.38(1)	**+0.09**	1.38(2)	**+0.09**	1.44(1)	**+0.15**	1.45(1)	**+0.16**
P	1.387(1)	1.385(1)	0	1.39(1)	0	1.36(1)	-0.02	1.37(1)	-0.01	1.35(1)	-0.03	1.36(1)	-0.02

M-C(ave)	-	2.028(7)	-	2.03(2)	-	1.99(1)	-0.03	2.05(5)	+0.02	2.06(1)	+0.04	2.08(1)	**+0.05**
M-N(ave)	-	2.153(4)	-	2.106(8)	-	2.172(6)	+0.02	2.10(2)	0	2.120(2)	-0.03	2.11(2)	0
Yb-Cp*(ctr, ave)	-	-	-			2.29(1)	-	2.28(1)	-	2.31(1)	-	2.306(7)	-
										2.34(2)		2.35(3)	
Yb-N(ave)	-	-	-			2.29(3)	-	2.29(2	-	2.21(2)	-	2.22(1)	-
Yb-C(ave)	-	-	-			-	-	-	-	2.566(6)	-	2.58(2)	-

**Table 2:** DFT/PBE0-D3 energy decomposition analysis

Energy (kcal.mol^-1^)	5	{[(tmeda)PdMe_2_](Cp*_2_Yb)}	Cp*_2_Yb(thf)_2_
Pauli repulsion	67	60	54
Electrostatic interaction	-107	-47	-53
Orbital interaction	-79	-28	-25
Dispersion	-18	-15	-18
Bond Strength	35	24	31
