# Supplementary material for: Reversible electron transfer in organolanthanide chemistry
Source: Chem Sq. Author manuscript; Available in PMC 2019 Aug 28. (PMC6713560; doi:10.28954/2019.csq.06.001)
Supplement: Supporting information [file EMS83980-supplement-Supporting_information.pdf]

Supporting Information

Reversible electron transfer in organolanthanide chemistry

Arnaud Jaoul,[a] Maxime Tricoire,[a] Jules Moutet,[a] Marie Cordier,[a] Carine Clavaguéra[b]* and Grégory Nocton[a]*

greg.nocton@polytechnique.edu

[a] LCM, CNRS, Ecole polytechnique, IP Paris, Route de Saclay, Palaiseau, France

[b] Laboratoire de Chimie Physique, CNRS-Université Paris-Sud, Université Paris-Saclay, 15 avenue Jean Perrin, 91405 Orsay Cedex, France

Table of Contents.

^{1}H NMR data …………………………………………………………………………… p 3

Magnetic data …………………………………………………………………………… p 8

Theoretical data ………………………………………………………………………….. p 10

X-ray crystallography …………………………………………………………………….. p 16

^{1}H NMR data

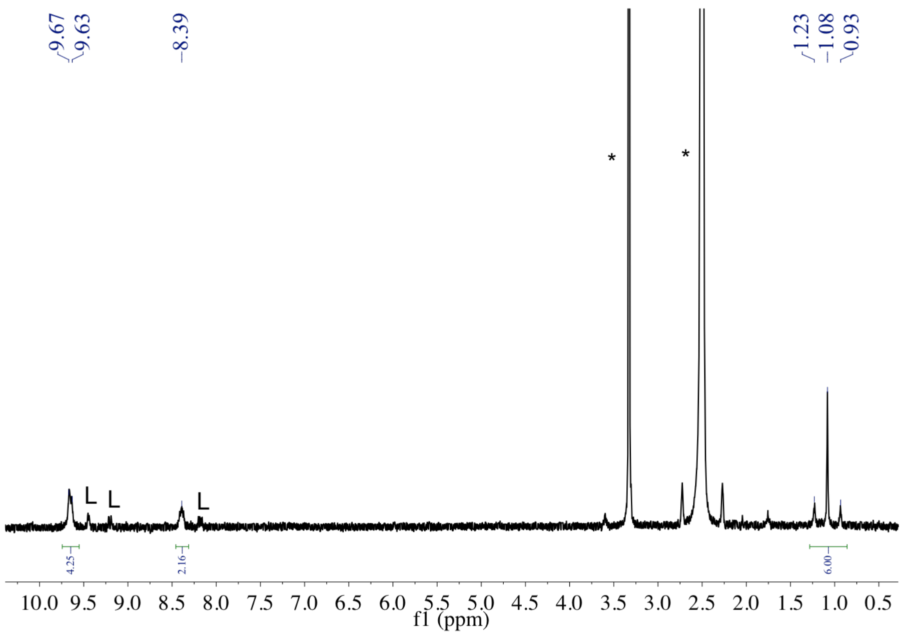

Figure S1. ^{1}H NMR of (taphen)PtMe$_2$, **2** in dmso-d$_6$ at 300 K. L are for free taphen ligand displaced by dmso and * for solvents and impurities.

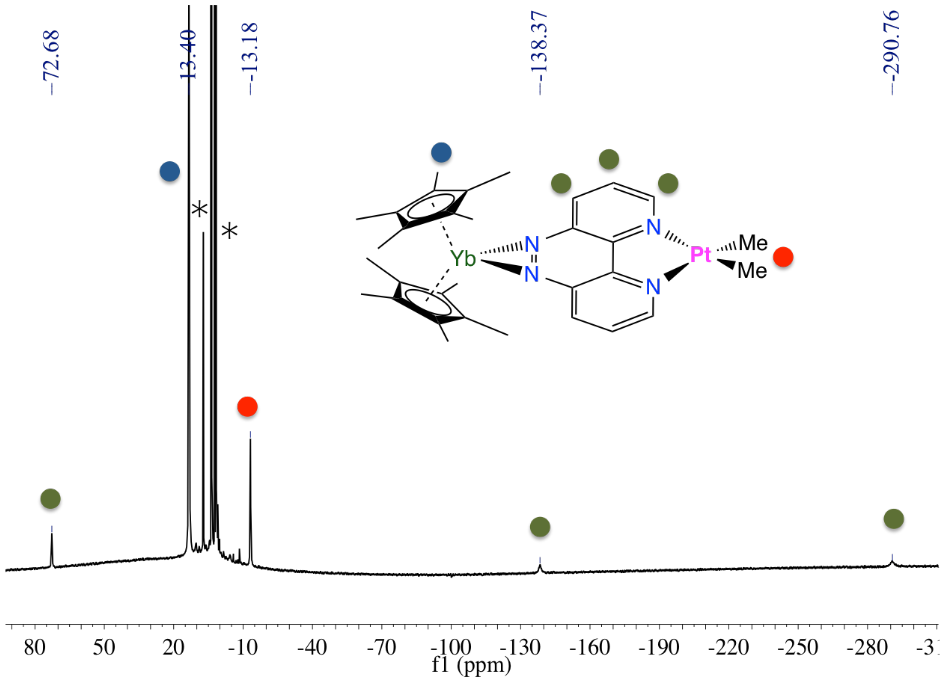

Figure S2. ^{1}H NMR of Cp*$_2$Yb(taphen)PtMe$_2$, **4** in thf-d$_8$ at 300 K.

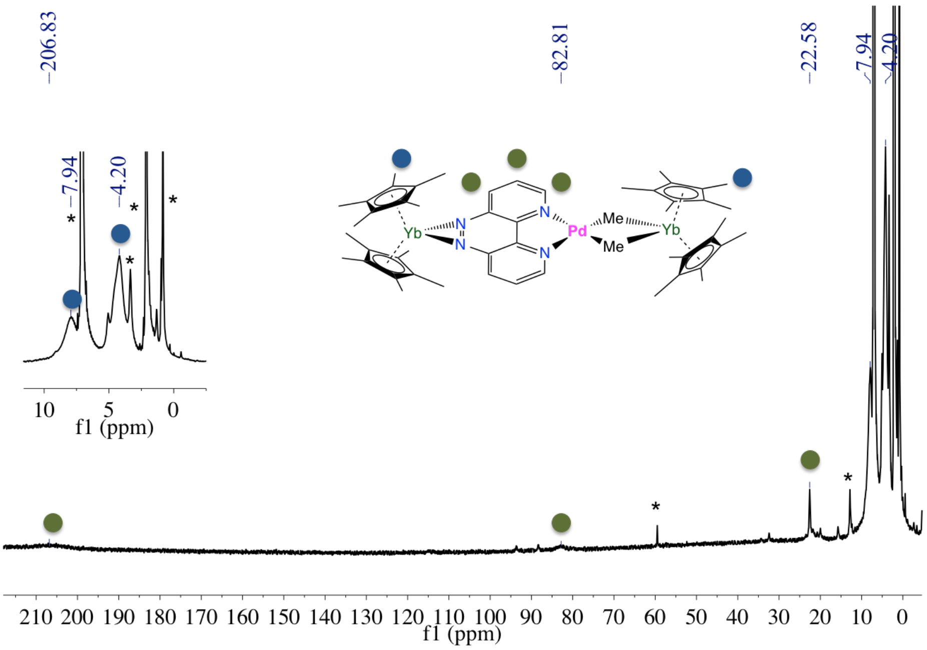

Figure S3. ^{1}H NMR of {[Cp*$_2$Yb(taphen)PdMe$_2$](Cp*$_2$Yb)}, **5** in toluene-d$_8$ at 293 K made *in situ* after addition of 1 equivalent of Cp*$_2$Yb(OEt$_2$).

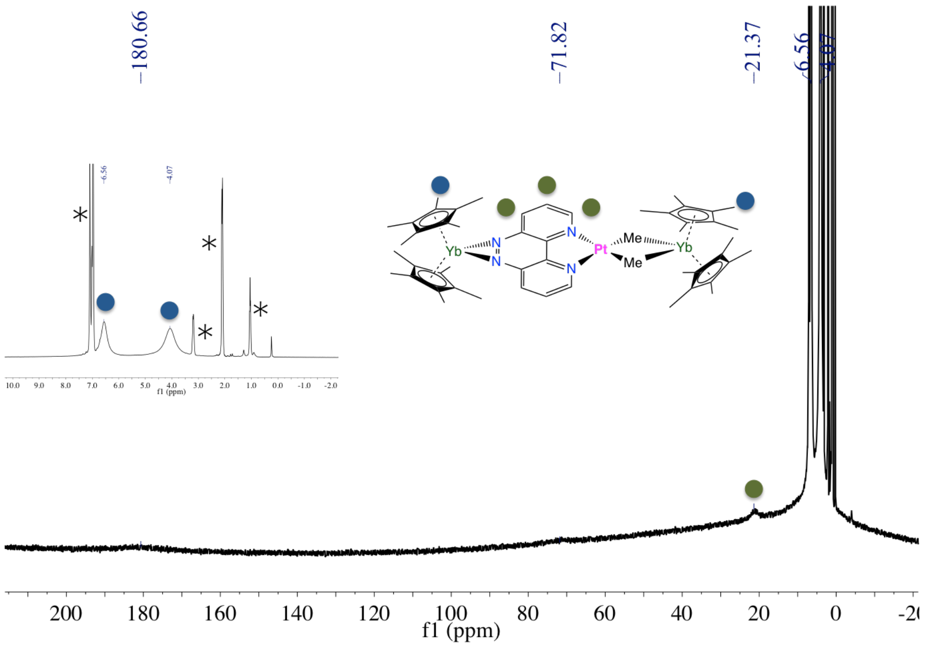

Figure S4. ^{1}H NMR of {[Cp*$_2$Yb(taphen)PtMe$_2$](Cp*$_2$Yb)}, **6** in toluene-d$_8$ at 333 K made *in situ* after addition of 1 equivalent of Cp*$_2$Yb(OEt$_2$). * correspond to a residual Cp* signal of **4** (at 12.68 ppm), to diethyl ether and to ^{1}H residue of toluene. The resonance of the methyl groups are not observed. Because of the broadness of the peaks, the resonances at 180.66 and 71.82 were not definitely assigned.

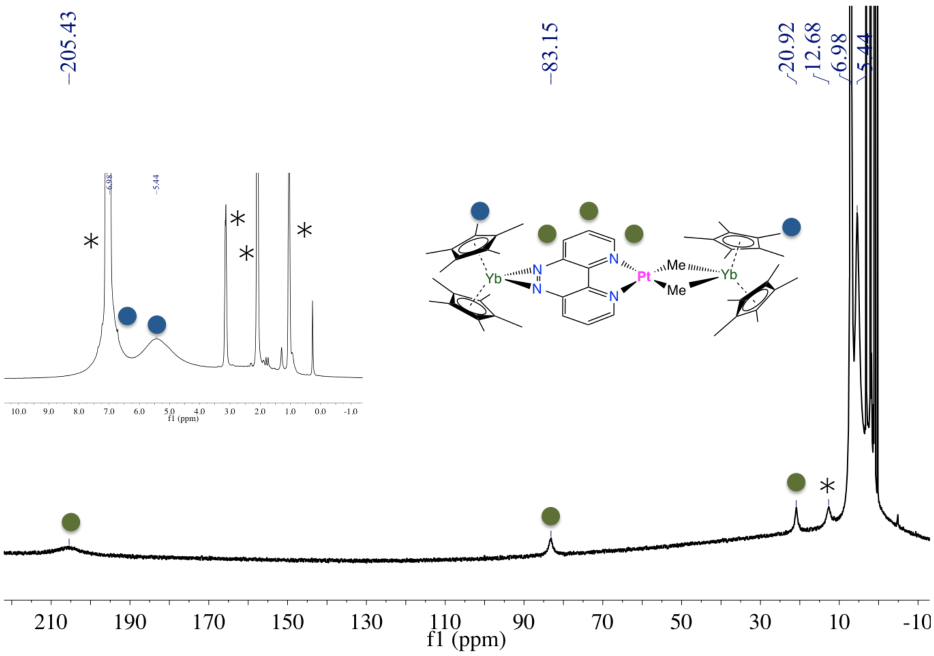

Figure S5. ^{1}H NMR of {[Cp*$_2$Yb(taphen)PtMe$_2$](Cp*$_2$Yb)}, **6** in toluene-d$_8$ at 293 K made *in situ* after addition of 1 equivalent of Cp*$_2$Yb(OEt$_2$). * correspond to a residual Cp* signal of **4** (at 12.68 ppm), to diethyl ether and to ^{1}H residue of toluene. The resonance of the methyl groups are not observed.

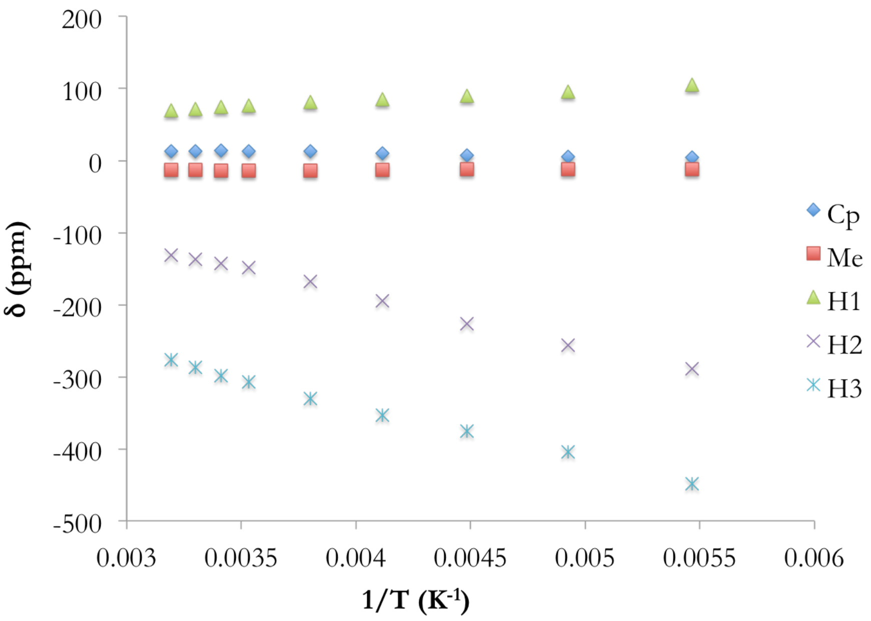

Figure S6. VT ^{1}H NMR of Cp*$_2$Yb(taphen)PtMe$_2$, **4** in toluene-d$_8$.

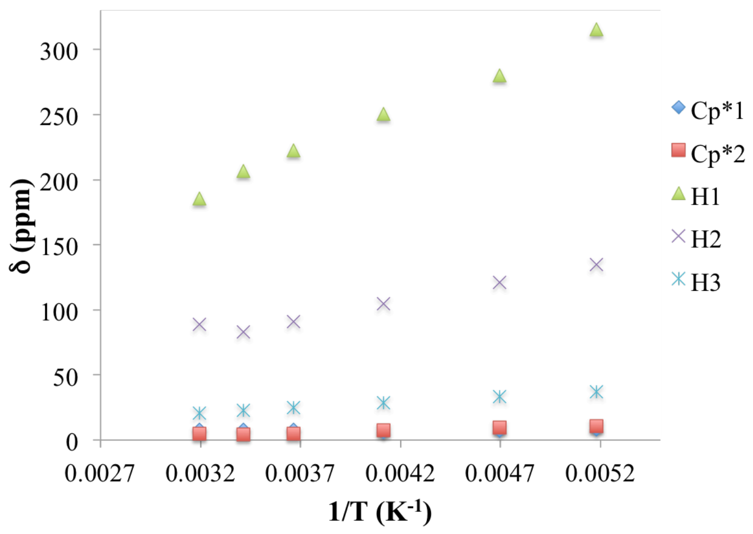

Figure S7. VT ^{1}H NMR of the {[Cp*$_2$Yb(taphen)PdMe$_2$](Cp*$_2$Yb)} complex, **5** in toluene-d$_8$.

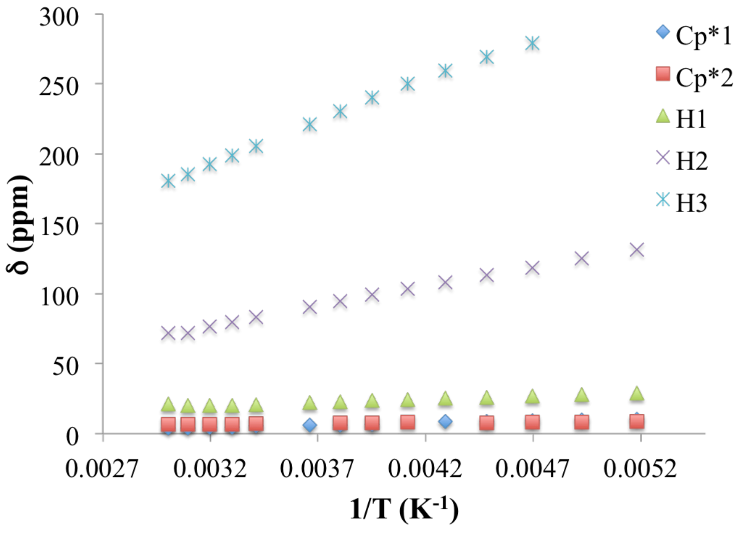

Figure S8. VT ^{1}H NMR of the {[Cp*$_2$Yb(taphen)PtMe$_2$](Cp*$_2$Yb)} complex, **6** in toluene-d$_8$.

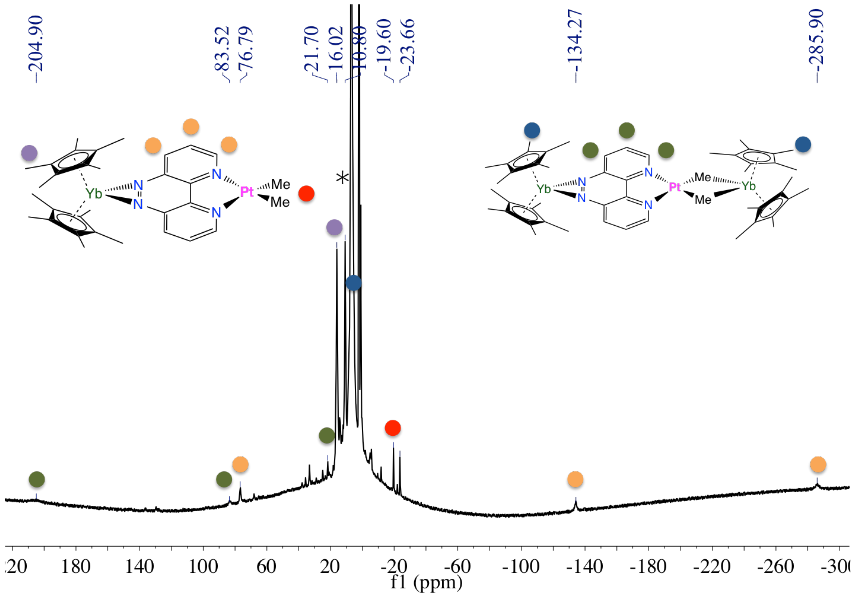

Figure S9. ^{1}H NMR at 20 °C in toluene-d_8 of **6** after addition of 1 equivalent of MeI. * are for solvents. The resonances characteristic of **6** and **4** are mixed together with an additional unidentified species.

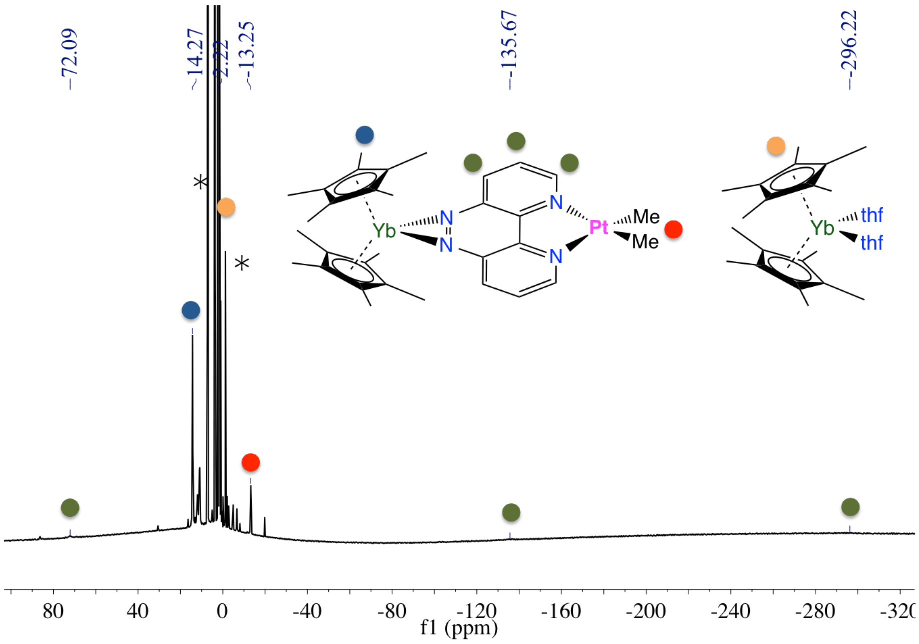

Figure S10. ^{1}H NMR at 20 °C in toluene-d_8 of **6** after addition of 10 equivalent of thf. * are for solvents. The resonances characteristic of **4** and $Cp^*_2Yb(thf)_2$ are mixed together.

Magnetic data

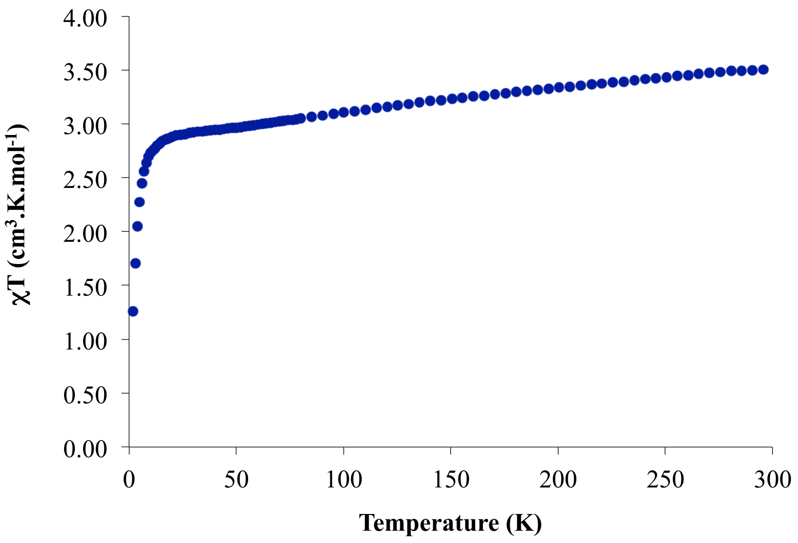

Figure S11. Temperature magnetic data for [Cp*$_2$Yb(taphen)PtMe$_2$], **4**. χT vs. T.

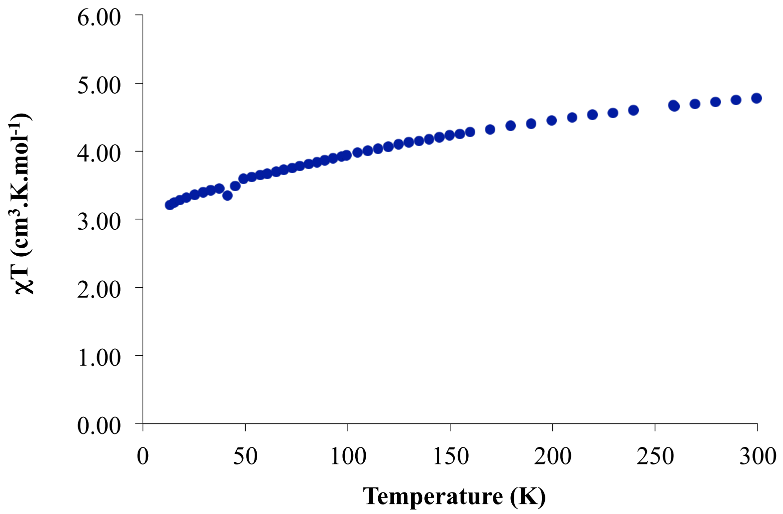

Figure S12. Temperature magnetic data for {[Cp*$_2$Yb(taphen)PdMe$_2$](Cp*$_2$Yb)}, **5**. χT vs. T.

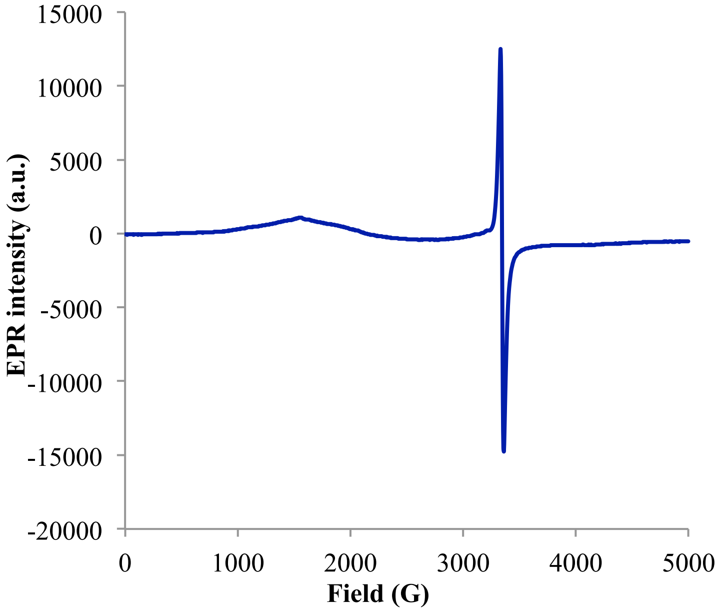

Figure S13. X-band (9.387652 GHz) EPR data for [$Cp^*{}_2Yb$(taphen)$PdMe_2$], **3** recorded at low temperature (10 K) (Power, 1.008 mW; Power attenuation, 23 dB).

Theoretical data

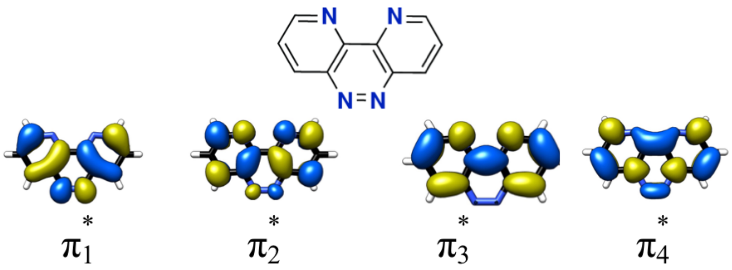

Figure S14. Valence molecular orbitals of the dianion taphen ligand at the CASSCF(8,7) level. Other π orbitals that are not represented are doubly occupied.

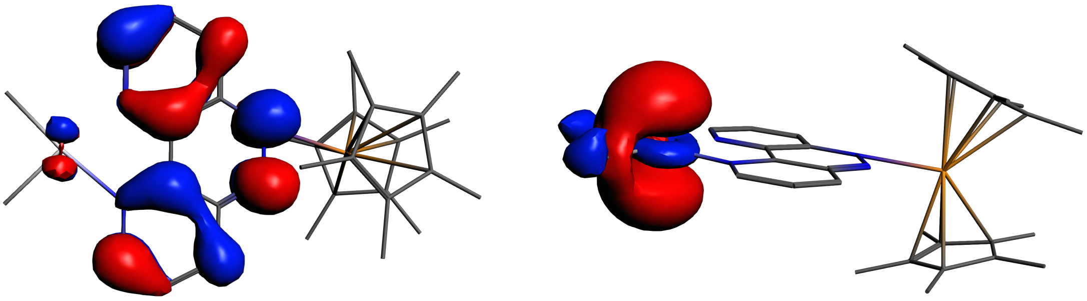

Figure S15. The two highest occupied molecular orbitals for **4.**

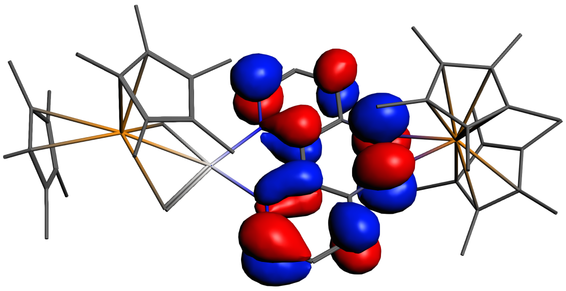

Figure S16. HOMO for **6.**

Table S1. Decomposition energy for **3**.

Energy (kcal/mol)	**3**
Pauli repulsion	74
Electrostatic interaction	-128
Orbital interaction	-84
Dispersion	-11
Bond Strength	60

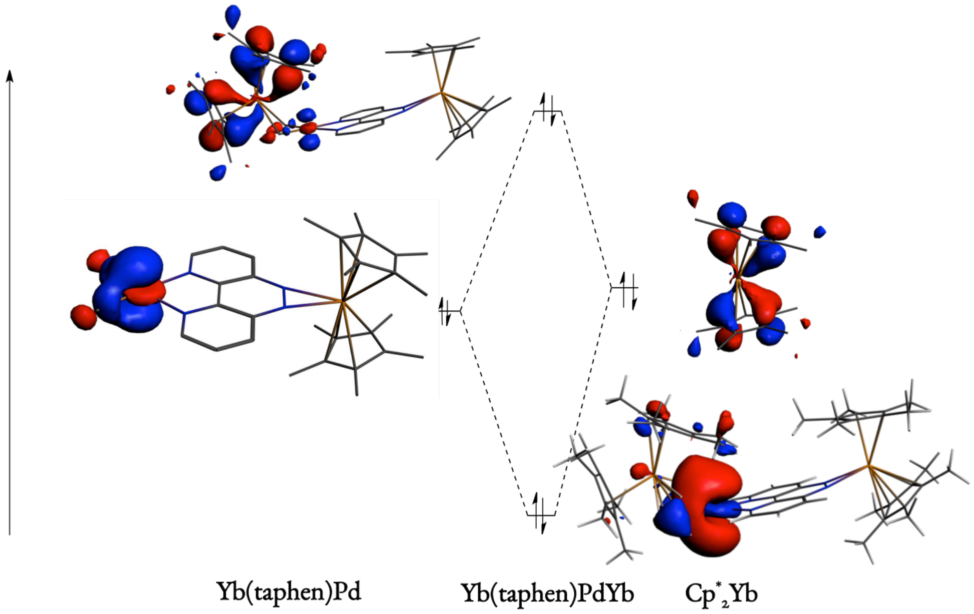

Figure S17. Orbital interaction diagram to explain the bent structure of **5**.

Table S2. Coordinates for **3**.

Yb	1.954525	-4.222103	6.527257
Yb	1.294276	4.878938	2.609163
Pd	2.093274	1.790520	2.210323
N	3.443330	0.603655	3.373732
N	0.903469	0.023429	2.479042
N	2.737960	-2.500890	5.216184
N	1.495120	-2.790142	4.771987
C	4.686175	0.877285	3.820300
H	5.157352	1.787793	3.435997
C	5.351107	0.041060	4.742313
H	6.360188	0.311383	5.072749
C	4.729642	-1.106418	5.230250
H	5.214916	-1.773360	5.951113
C	3.416746	-1.415428	4.767062
C	2.827089	-0.517526	3.821869
C	1.503004	-0.822875	3.352700
C	0.875113	-2.006213	3.857392
C	-0.432151	-2.307602	3.371649
H	-0.941677	-3.208103	3.731440
C	-1.017218	-1.437553	2.456252
H	-2.019444	-1.635652	2.060424
C	-0.330428	-0.276840	2.034685
H	-0.788336	0.433517	1.337631
C	3.402413	3.367918	2.109456
H	3.201813	4.183784	1.386381
H	4.355275	2.932108	1.757634
H	3.601775	3.800123	3.117238
C	0.616708	2.744589	1.150705
H	0.877049	3.651384	0.573985
H	-0.272416	2.946094	1.791046
H	0.297159	2.018976	0.380749
C	3.872660	-5.689715	5.518715
C	3.431392	-6.385921	6.693785
C	2.091587	-6.845472	6.457799
C	1.705895	-6.419367	5.142301
C	2.812004	-5.718306	4.556524
C	5.241224	-5.098594	5.315691
H	5.978435	-5.863395	4.999108
H	5.638969	-4.644039	6.242718
H	5.234568	-4.314761	4.537774
C	4.298082	-6.724399	7.876090
H	3.701025	-7.079039	8.732313
H	4.895798	-5.861929	8.226313
H	5.020220	-7.527668	7.627685
C	1.300913	-7.766226	7.344958
H	1.521744	-8.828963	7.120357
H	0.212453	-7.631833	7.219367
H	1.526366	-7.610829	8.414404
C	0.393347	-6.711327	4.466036
H	0.422437	-7.657124	3.889007
H	0.116045	-5.913075	3.753232
H	-0.433673	-6.809053	5.193023
C	2.874307	-5.172818	3.157849
H	3.525368	-4.281646	3.094365
H	1.877281	-4.874747	2.787436
H	3.276469	-5.919034	2.444725
C	2.097752	-2.826035	8.742189
C	1.500242	-4.081617	9.105553
C	0.211994	-4.150304	8.478757
C	0.010936	-2.930133	7.744017
C	1.168618	-2.108950	7.917116
C	3.429599	-2.310552	9.216646
H	4.130846	-3.132084	9.450544
H	3.332650	-1.703488	10.138836
H	3.914220	-1.663209	8.462170
C	2.057303	-5.049235	10.112008
H	1.740516	-4.785474	11.140894
H	3.160830	-5.058121	10.108860
H	1.722488	-6.084352	9.928167
C	-0.829079	-5.219282	8.674748
H	-1.669502	-4.853005	9.296898
H	-0.413800	-6.105517	9.181452
H	-1.271230	-5.565764	7.720620
C	-1.248354	-2.548189	7.017264
H	-1.968059	-2.035112	7.686554
H	-1.772842	-3.431667	6.609034
H	-1.049888	-1.861853	6.174987
C	1.337800	-0.703343	7.413860
H	0.859709	-0.546998	6.429660
H	2.401285	-0.426911	7.308125
H	0.881723	0.033507	8.104016
C	2.647603	6.607818	1.071874
C	1.828321	5.875258	0.152306
C	0.457930	6.197077	0.434609
C	0.433265	7.159665	1.493884
C	1.790955	7.411647	1.891821
C	4.150584	6.687322	1.071283
H	4.505990	7.596897	0.546236
H	4.613075	5.822734	0.564725
H	4.567652	6.729908	2.094665
C	2.323977	5.046565	-1.001496
H	1.553063	4.345998	-1.367970
H	3.213706	4.441584	-0.746685
H	2.607011	5.687925	-1.859193
C	-0.751925	5.761226	-0.347235
H	-0.575193	4.825927	-0.906235
H	-1.050945	6.528808	-1.089300
H	-1.628911	5.594661	0.304997
C	-0.786538	7.952180	1.873915
H	-0.593314	8.617776	2.728844
H	-1.650372	7.315468	2.139397
H	-1.113706	8.590040	1.028876
C	2.294088	8.507638	2.789552
H	3.029674	8.150197	3.533615
H	1.477758	9.001558	3.340647
H	2.802838	9.293893	2.196866
C	1.546174	5.334707	5.204942
C	1.434587	3.906955	5.110872
C	0.112252	3.594403	4.655344
C	-0.592922	4.826607	4.468502
C	0.279470	5.902438	4.852866
C	2.726124	6.069241	5.782296
H	3.689755	5.654194	5.431136
H	2.743182	6.007757	6.889556
H	2.711661	7.140826	5.519853
C	2.454782	2.939212	5.636688
H	2.528639	3.008145	6.740352
H	3.471392	3.126679	5.242862
H	2.200480	1.897367	5.387598
C	-0.519368	2.232455	4.607025
H	-1.187435	2.075075	5.477284
H	0.239591	1.435351	4.625495
H	-1.132552	2.074055	3.701480
C	-2.051350	4.936042	4.113284
H	-2.344636	4.204758	3.336838
H	-2.308000	5.939013	3.731713
H	-2.704457	4.745250	4.988797
C	-0.155398	7.305526	5.164711
H	-0.357680	7.404054	6.250263
H	-1.084770	7.579707	4.641197
H	0.608109	8.060101	4.914655

Table S3. Coordinates for **4**.

Pt	3.79975	-1.39494	15.68661	H	12.72317	5.35190	12.73486
Yb	9.62596	3.23576	15.53173	H	12.58375	5.63746	14.48112
N	5.82064	-1.29320	16.31042	H	11.25300	6.10281	13.39106
N	4.34267	0.51394	14.92996	C	9.38546	4.32962	12.11145
N	7.56020	2.28812	15.13715	H	9.19935	5.33605	12.53128
N	8.29178	1.39151	15.85066	H	8.40310	3.85631	11.93719
C	6.41514	-0.12972	15.94415	H	9.84959	4.48431	11.11757
C	5.63665	0.82284	15.20212	C	10.00930	3.76735	18.06128
C	3.61047	1.40637	14.22392	C	8.59943	3.84972	17.85929
H	2.57246	1.12454	14.01898	C	8.34565	4.96538	16.98363
C	4.15398	2.62713	13.77831	C	9.59786	5.59061	16.67519
H	3.51557	3.31348	13.20996	C	10.62989	4.85724	17.34611
C	5.47986	2.96574	14.05826	C	12.06750	5.26447	17.48311
H	5.92190	3.91203	13.72800	H	12.43122	5.82678	16.60580
C	6.26630	2.04098	14.80300	H	12.73553	4.39793	17.62647
C	7.76490	0.20904	16.26306	H	12.20511	5.92395	18.36392
C	8.51107	-0.73316	17.02594	C	9.75745	6.87753	15.91380
H	9.54685	-0.50844	17.30285	H	9.45284	7.74734	16.52892
C	7.88102	-1.92379	17.39974	H	9.13651	6.90706	14.99908
H	8.42100	-2.67613	17.98631	H	10.80182	7.04648	15.60673
C	6.54766	-2.18110	17.03070	C	6.99542	5.45982	16.54810
H	6.03739	-3.10914	17.30989	H	7.01697	5.86988	15.52175
C	10.25372	3.49080	13.00950	H	6.63328	6.27546	17.20538
C	11.40693	3.96399	13.73744	H	6.23479	4.66005	16.57014
C	11.97580	2.84944	14.43781	C	7.54895	2.98425	18.49406
C	11.18037	1.69442	14.13979	H	7.17406	3.43617	19.43281
C	10.12923	2.08808	13.23762	H	7.93197	1.98156	18.74759
C	9.12860	1.16367	12.60719	H	6.67599	2.84560	17.83235
H	8.20645	1.69319	12.31182	C	10.73138	2.77084	18.92719
H	8.83201	0.34635	13.28772	H	10.96244	3.18563	19.92791
H	9.53979	0.69229	11.69336	H	11.69676	2.45747	18.48716
C	11.45432	0.29589	14.61501	H	10.12809	1.86000	19.08745
H	12.10208	-0.25500	13.90339	C	3.46974	-3.25593	16.44579
H	10.52681	-0.29248	14.72979	H	4.25594	-3.95616	16.09528
H	11.97827	0.28985	15.58789	H	2.49143	-3.69262	16.17198
C	13.27300	2.83311	15.20017	H	3.51535	-3.21709	17.55460
H	14.07913	2.36246	14.60322	C	1.86364	-1.29922	15.06623
H	13.20709	2.26003	16.14403	H	1.83092	-1.37509	13.95762
H	13.61156	3.84932	15.45776	H	1.41417	-0.32591	15.35337
C	12.01455	5.32838	13.58754	H	1.21157	-2.09591	15.46881

Table S4. Coordinates for **5**.

Yb	1.95452	-4.22210	6.52726
Yb	1.29428	4.87894	2.60916
Pd	2.09327	1.79052	2.21032
N	3.44333	0.60366	3.37373
N	0.90347	0.02343	2.47904
N	2.73796	-2.50089	5.21618
N	1.49512	-2.79014	4.77199
C	4.68617	0.87729	3.82030
H	5.15735	1.78779	3.43600
C	5.35111	0.04106	4.74231
H	6.36019	0.31138	5.07275
C	4.72964	-1.10642	5.23025
H	5.21492	-1.77336	5.95111
C	3.41675	-1.41543	4.76706
C	2.82709	-0.51753	3.82187
C	1.50300	-0.82288	3.35270
C	0.87511	-2.00621	3.85739
C	-0.43215	-2.30760	3.37165
H	-0.94168	-3.20810	3.73144
C	-1.01722	-1.43755	2.45625
H	-2.01944	-1.63565	2.06042
C	-0.33043	-0.27684	2.03469
H	-0.78834	0.43352	1.33763
C	3.40241	3.36792	2.10946
H	3.20181	4.18378	1.38638
H	4.35527	2.93211	1.75763
H	3.60177	3.80012	3.11724
C	0.61671	2.74459	1.15070
H	0.87705	3.65138	0.57398
H	-0.27242	2.94609	1.79105
H	0.29716	2.01898	0.38075
C	3.87266	-5.68972	5.51871
C	3.43139	-6.38592	6.69378
C	2.09159	-6.84547	6.45780
C	1.70590	-6.41937	5.14230
C	2.81200	-5.71831	4.55652
C	5.24122	-5.09859	5.31569
H	5.97844	-5.86339	4.99911
H	5.63897	-4.64404	6.24272
H	5.23457	-4.31476	4.53777
C	4.29808	-6.72440	7.87609
H	3.70103	-7.07904	8.73231
H	4.89580	-5.86193	8.22631
H	5.02022	-7.52767	7.62768
C	1.30091	-7.76623	7.34496
H	1.52174	-8.82896	7.12036
H	0.21245	-7.63183	7.21937
H	1.52637	-7.61083	8.41440
C	0.39335	-6.71133	4.46604
H	0.42244	-7.65712	3.88901
H	0.11605	-5.91307	3.75323
H	-0.43367	-6.80905	5.19302
C	2.87431	-5.17282	3.15785
H	3.52537	-4.28165	3.09436
H	1.87728	-4.87475	2.78744
H	3.27647	-5.91903	2.44473
C	2.09775	-2.82604	8.74219
C	1.50024	-4.08162	9.10555
C	0.21199	-4.15030	8.47876
C	0.01094	-2.93013	7.74402
C	1.16862	-2.10895	7.91712
C	3.42960	-2.31055	9.21665
H	4.13085	-3.13208	9.45054
H	3.33265	-1.70349	10.13884
H	3.91422	-1.66321	8.46217
C	2.05730	-5.04924	10.11201
H	1.74052	-4.78547	11.14089
H	3.16083	-5.05812	10.10886
H	1.72249	-6.08435	9.92817
C	-0.82908	-5.21928	8.67475
H	-1.66950	-4.85300	9.29690
H	-0.41380	-6.10552	9.18145
H	-1.27123	-5.56576	7.72062
C	-1.24835	-2.54819	7.01726
H	-1.96806	-2.03511	7.68655
H	-1.77284	-3.43167	6.60903
H	-1.04989	-1.86185	6.17499
C	1.33780	-0.70334	7.41386
H	0.85971	-0.54700	6.42966
H	2.40129	-0.42691	7.30812
H	0.88172	0.03351	8.10402
C	2.64760	6.60782	1.07187
C	1.82832	5.87526	0.15231
C	0.45793	6.19708	0.43461
C	0.43326	7.15967	1.49388
C	1.79095	7.41165	1.89182
C	4.15058	6.68732	1.07128
H	4.50599	7.59690	0.54624
H	4.61307	5.82273	0.56473
H	4.56765	6.72991	2.09467
C	2.32398	5.04656	-1.00150
H	1.55306	4.34600	-1.36797
H	3.21371	4.44158	-0.74669
H	2.60701	5.68792	-1.85919
C	-0.75192	5.76123	-0.34723
H	-0.57519	4.82593	-0.90623
H	-1.05094	6.52881	-1.08930
H	-1.62891	5.59466	0.30500
C	-0.78654	7.95218	1.87392
H	-0.59331	8.61778	2.72884
H	-1.65037	7.31547	2.13940
H	-1.11371	8.59004	1.02888
C	2.29409	8.50764	2.78955
H	3.02967	8.15020	3.53361
H	1.47776	9.00156	3.34065
H	2.80284	9.29389	2.19687
C	1.54617	5.33471	5.20494
C	1.43459	3.90696	5.11087
C	0.11225	3.59440	4.65534
C	-0.59292	4.82661	4.46850
C	0.27947	5.90244	4.85287
C	2.72612	6.06924	5.78230
H	3.68976	5.65419	5.43114
H	2.74318	6.00776	6.88956
H	2.71166	7.14083	5.51985
C	2.45478	2.93921	5.63669
H	2.52864	3.00815	6.74035
H	3.47139	3.12668	5.24286
H	2.20048	1.89737	5.38760
C	-0.51937	2.23245	4.60703
H	-1.18744	2.07508	5.47728
H	0.23959	1.43535	4.62550
H	-1.13255	2.07406	3.70148
C	-2.05135	4.93604	4.11328
H	-2.34464	4.20476	3.33684
H	-2.30800	5.93901	3.73171
H	-2.70446	4.74525	4.98880
C	-0.15540	7.30553	5.16471
H	-0.35768	7.40405	6.25026
H	-1.08477	7.57971	4.64120
H	0.60811	8.06010	4.91465

Table S5. Coordinates for **6**.

Pt	4.61834	6.17239	6.55673
Yb	5.38454	3.07317	6.17192
Yb	4.76253	12.18351	2.25348
N	3.29285	7.33345	5.40091
N	5.80931	7.88779	6.27202
N	5.23220	10.72922	4.01198
N	3.97221	10.45624	3.58326
C	6.56897	4.30875	4.10646
C	5.24560	3.98662	3.66010
C	5.13452	2.55838	3.59434
C	6.40376	1.99768	3.95771
C	7.27510	3.08103	4.31857
C	8.73601	2.98328	4.66737
H	9.00823	1.97735	5.02868
H	9.02564	3.70552	5.45402
H	9.38256	3.19545	3.79161
C	6.84194	0.59045	3.66947
H	7.74907	0.31045	4.22899
H	7.08553	0.48620	2.59281
H	6.06539	-0.15838	3.89319
C	3.95588	1.81202	3.02971
H	3.97041	0.74575	3.31292
H	3.94018	1.85171	1.92102
H	2.99081	2.23111	3.37267
C	4.22103	4.94751	3.12879
H	4.45457	5.98731	3.40596
H	3.20098	4.73598	3.50006
H	4.17168	4.90061	2.02212
C	7.20095	5.67085	4.12739
H	7.80672	5.85556	5.03369
H	6.44137	6.46712	4.08276
H	7.87899	5.80488	3.26030
C	4.00173	1.33564	7.67131
C	4.90982	0.53917	6.90008
C	6.24383	0.82934	7.34902
C	6.15265	1.80070	8.39609
C	4.76510	2.09376	8.61824
C	4.21006	2.91938	9.74700
H	4.17214	2.33172	10.68545
H	3.18112	3.26435	9.54524
H	4.81550	3.82000	9.95728
C	7.31660	2.26819	9.22734
H	7.06648	3.15606	9.83353
H	8.19537	2.52746	8.60766
H	7.64855	1.47968	9.93286
C	7.50291	0.07676	7.02221
H	7.35374	-0.64294	6.20235
H	7.85101	-0.49946	7.90299
H	8.33868	0.73965	6.73235
C	4.46867	-0.59652	6.01906
H	5.32216	-1.11161	5.55079
H	3.78432	-0.28191	5.21016
H	3.92201	-1.35533	6.61448
C	2.50271	1.23650	7.58892
H	2.00054	2.10968	8.03976
H	2.12428	0.33949	8.12027
H	2.14869	1.15750	6.54411
C	3.89433	8.46367	4.95167
C	5.22213	8.75595	5.41087
C	7.05537	8.16871	6.71837
H	7.50305	7.45066	7.41213
C	7.74829	9.32232	6.29895
H	8.75517	9.50319	6.69079
C	7.17251	10.21317	5.39456
H	7.69612	11.10861	5.04254
C	5.85691	9.93602	4.91370
C	3.29253	9.37374	4.02899
C	1.96879	9.06854	3.58963
H	1.46553	9.74159	2.88674
C	1.35934	7.91458	4.07944
H	0.34378	7.64856	3.76654
C	2.03574	7.06439	4.97734
H	1.57405	6.14888	5.35636
C	2.86968	13.63010	3.32335
C	3.95674	13.66511	4.25233
C	5.04321	14.37128	3.63373
C	4.61641	14.80135	2.33298
C	3.27258	14.33475	2.13738
C	2.37213	14.65922	0.97720
H	1.77901	13.78646	0.64383
H	2.94233	15.02025	0.10500
H	1.64383	15.45146	1.24196
C	5.37117	15.73141	1.42445
H	6.46475	15.60076	1.50794
H	5.15457	16.79252	1.66509
H	5.10554	15.58460	0.36218
C	6.37123	14.66702	4.27644
H	7.15626	14.86483	3.52480
H	6.71841	13.82827	4.90862
H	6.32357	15.55902	4.93309
C	3.94408	13.11995	5.65242
H	3.30482	12.22269	5.73661
H	3.55769	13.86499	6.37585
H	4.95498	12.83239	5.99045
C	1.51150	13.02894	3.56112
H	1.09785	12.56058	2.64779
H	0.77463	13.79173	3.88314
H	1.54229	12.25255	4.34642
C	6.66422	10.89545	0.99287
C	6.44993	12.11530	0.26384
C	5.14522	12.05234	-0.33397
C	4.55311	10.80012	0.04224
C	5.50014	10.07837	0.84442
C	5.33625	8.67217	1.34769
H	5.83852	8.50965	2.31833
H	5.77144	7.93714	0.64214
H	4.27481	8.40026	1.47987
C	3.21046	10.28653	-0.40475
H	2.50822	11.10924	-0.63097
H	2.73660	9.64499	0.36161
H	3.29115	9.67247	-1.32439
C	4.57336	13.02071	-1.33133
H	3.46961	13.03489	-1.30963
H	4.87169	12.75428	-2.36577
H	4.91658	14.05567	-1.15821
C	7.48508	13.18737	0.05162
H	7.05596	14.08030	-0.43060
H	8.30798	12.82919	-0.59859
H	7.95489	13.52174	0.99711
C	7.93381	10.51085	1.70123
H	7.74253	9.83532	2.55441
H	8.47188	11.39351	2.09356
H	8.63739	9.98418	1.02463
C	3.28302	4.62145	6.69157
H	3.50014	3.81543	7.42259
H	3.06713	4.15484	5.70018
H	2.31412	5.02184	7.04747
C	6.09648	5.22955	7.62245
H	7.00607	5.05539	7.00084
H	5.85325	4.28809	8.15761
H	6.41440	5.89826	8.44561

X-ray crystallography

Table S6. Selected Crystal Data Collection Parameters for the taphen ligand (taphen), (taphen)$PtMe_2$ (**2**) and Cp^*_2Yb(taphen)$PtMe_2$ **(3)**.

**Compound**	**Taphen**	**(taphen)$PtMe_2$ (2)**	**Cp^*_2Yb(taphen)$PtMe_2$ (3).**
Formula	$2(C_{10}H_6N_4),CH_2Cl_2$	$C_{12}H_{12}N_4Pt$	$C_{32}H_{42}N_4PtYb$
Crystal size (mm)	0.280x0.100x0.100	0.300x0.100x0.020	0.100x0.060x0.010
Crystal system	triclinic	monoclonic	triclinic
Space group	P -1	P 2_1/c	P -1
Volume (Å)	967.31(8)	1337.35(7)	3745.97(14)
a (Å)	3.8606(2)	6.8917(2)	13.4500(3)
b (Å)	14.8724(7)	14.0417(4)	15.9392(3)
c (Å)	17.6816(8)	14.0951(4)	19.6119(4)
α (deg)	105.797(2)	90	88.664(1)
β (deg)	95.852(2)	101.345(2)	72.997(1)
γ (deg)	93.668(1)	90	69.298(1)
Z	2	4	4
Formula weight –g/mol)	449.30	407.35	850.82
Density (calcd) (g/cm^{-3})	1.543	2.023	1.509
Absorption coefficient (cm^{-1})	0.365	10.476	6.233
F(000)	460	760	1640
Temp (K)	150	150	150
diffractometer[a]	Kappa APEX II CCD	Kappa APEX II CCD	Kappa APEX II CCD
θ range for data collection (deg)	1.206 – 27.519	2.068 – 30.157	1.717 – 25.025
Absorption correction	Multi-scan	Multi-scan	Multi-scan
Total no. reflections	10103	27119	58857
Unique reflections [R_{int}]	4092 [0.0327]	3931 [0.0893]	13184 [0.0820]
Final R[b] indices [I>2σ(I)]	R = 0.0538, R_w = 0.1678	R = 0.0406, R_w = 0.0924	R = 0.0792, R_w = 0.1925
R indices (all data)	R = 0.0597, R_w = 0.1750	R = 0.0520, R_w = 0.0983	R = 0.1038, R_w = 0.2086
Largest diff. peak and hole (e.A^{-3})	0.560(0.068) / -0.314(0.068)	1.631(0.209) / -1.636(0.209)	5.869(0.259) / -4.829(0.259)
GooF	1.020	1.074	1.086

Table S7. Selected Crystal Data Collection Parameters {[Cp*$_2$Yb(taphen)PdMe$_2$](Cp*$_2$Yb)} (**5**) and {[Cp*$_2$Yb(taphen)PtMe$_2$](Cp*$_2$Yb)} **(6).**

**Compound**	**5**	**6**
Formula	C_{52} H_{72} N_4 Pd Yb_2	C_{52} H_{72} N_4 Pt Yb_2
Crystal size (mm)	0.100x0.050x0.050	0.300x0.070x0.020
Crystal system	triclinic	triclinic
Space group	P -1	P -1
Volume (Å)	2515.11(9)	2515.58(19)
a (Å)	8.5468(2)	8.6444(4)
b (Å)	16.9907(3)	16.9738(8)
c (Å)	17.5855(3)	17.6073(8)
α (deg)	92.099(1)	91.943(3)
β (deg)	90.278(1)	90.393(2)
γ (deg)	103.004(1)	102.997(3)
Z	2	2
Formula weight –g/mol)	1205.61	1294.30
Density (calcd) (g/cm^{-3})	1.592	1.709
Absorption coefficient (mm^{-1})	4.078	6.497
F(000)	1196	12600
Temp (K)	150	150
diffractometer[a]	Kappa APEX II CCD	Kappa APEX II CCD
θ range for data collection (deg)	2.318 – 30.030	2.418 – 25.349
Absorption correction	Multi-scan	Multi-scan
Total no. reflections	56905	29999
Unique reflections [R_{int}]	14668 [0.0930]	9118 [0.0865]
Final R[b] indices [I>2σ(I)]	R = 0.0652, R_w = 0.1605	R = 0.0613, R_w = 0.1146
R indices (all data)	R = 0.0968, R_w = 0.1830	R = 0.0913, R_w = 0.1273
Largest diff. peak and hole (e.A^{-3})	2.344(0.227) / -1.447(0.227)	1.709(0.224) / -1.517(0.224)
GooF	0.952	1.102

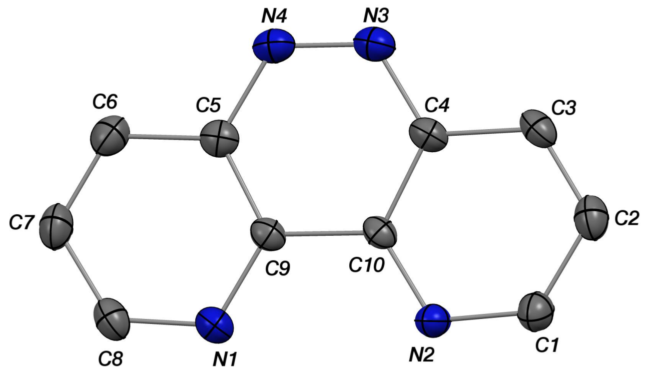

Figure S18. ORTEP of the taphen ligand. Ellipsoids are at 50% level. The hydrogen atoms have been removed for clarity.

Table S8. Bond distances (Å) and Angle (deg) for the taphen ligand.

N(1)-C(8)	1.322(4)	N(1)-C(9)	1.359(3)
N(2)-C(1)	1.327(3)	N(2)-C(10)	1.349(3)
N(3)-N(4)	1.292(3)	N(3)-C(4)	1.382(3)
N(4)-C(5)	1.387(3)	C(1)-C(2)	1.405(4)
C(1)-H(1)	0.98(4)	C(2)-C(3)	1.363(4)
C(2)-H(2)	0.97(4)	C(3)-C(4)	1.414(4)
C(3)-H(3)	0.86(4)	C(4)-C(10)	1.410(3)
C(5)-C(9)	1.404(4)	C(5)-C(6)	1.407(4)
C(6)-C(7)	1.361(4)	C(6)-H(6)	0.98(4)
C(7)-C(8)	1.412(4)	C(7)-H(7)	0.94(4)
C(8)-H(8)	0.97(4)	C(9)-C(10)	1.439(3)
N(5)-C(31)	1.326(4)	N(5)-C(40)	1.359(3)
N(6)-C(38)	1.324(4)	N(6)-C(39)	1.349(3)
N(7)-N(8)	1.294(3)	N(7)-C(35)	1.380(3)
N(8)-C(34)	1.386(3)	C(31)-C(32)	1.400(4)
C(31)-H(31)	0.97(4)	C(32)-C(33)	1.370(4)
C(32)-H(32)	0.93(4)	C(33)-C(34)	1.411(4)
C(33)-H(33)	0.94(4)	C(34)-C(40)	1.401(3)
C(35)-C(36)	1.411(4)	C(35)-C(39)	1.413(3)
C(36)-C(37)	1.366(4)	C(36)-H(36)	0.97(4)
C(37)-C(38)	1.409(4)	C(37)-H(37)	0.97(4)
C(38)-H(38)	0.96(4)	C(39)-C(40)	1.446(4)
Cl(1)-C(41)	1.776(3)	Cl(2)-C(41)	1.764(3)
C(41)-H(41A)	0.92(4)	C(41)-H(41B)	0.92(4)

C(8)-N(1)-C(9)	116.3(2)	C(1)-N(2)-C(10)	116.5(2)
N(4)-N(3)-C(4)	120.1(2)	N(3)-N(4)-C(5)	120.5(2)
N(2)-C(1)-C(2)	124.5(3)	N(2)-C(1)-H(1)	113(2)
C(2)-C(1)-H(1)	122(2)	C(3)-C(2)-C(1)	119.3(3)
C(3)-C(2)-H(2)	119(2)	C(1)-C(2)-H(2)	121(2)
C(2)-C(3)-C(4)	117.9(2)	C(2)-C(3)-H(3)	121(2)
C(4)-C(3)-H(3)	121(2)	N(3)-C(4)-C(10)	123.5(2)
N(3)-C(4)-C(3)	118.0(2)	C(10)-C(4)-C(3)	118.5(2)
N(4)-C(5)-C(9)	123.2(2)	N(4)-C(5)-C(6)	118.0(2)
C(9)-C(5)-C(6)	118.8(2)	C(7)-C(6)-C(5)	118.2(3)
C(7)-C(6)-H(6)	123(2)	C(5)-C(6)-H(6)	118(2)
C(6)-C(7)-C(8)	119.0(3)	C(6)-C(7)-H(7)	120(2)
C(8)-C(7)-H(7)	121(2)	N(1)-C(8)-C(7)	124.6(3)
N(1)-C(8)-H(8)	117(2)	C(7)-C(8)-H(8)	118(2)
N(1)-C(9)-C(5)	123.1(2)	N(1)-C(9)-C(10)	120.4(2)
C(5)-C(9)-C(10)	116.5(2)	N(2)-C(10)-C(4)	123.2(2)
N(2)-C(10)-C(9)	120.6(2)	C(4)-C(10)-C(9)	116.2(2)
C(31)-N(5)-C(40)	116.0(2)	C(38)-N(6)-C(39)	116.6(2)
N(8)-N(7)-C(35)	119.8(2)	N(7)-N(8)-C(34)	120.0(2)
N(5)-C(31)-C(32)	125.0(3)	N(5)-C(31)-H(31)	114(2)
C(32)-C(31)-H(31)	121(2)	C(33)-C(32)-C(31)	119.0(3)
C(33)-C(32)-H(32)	119(2)	C(31)-C(32)-H(32)	122(2)
C(32)-C(33)-C(34)	117.9(3)	C(32)-C(33)-H(33)	121(2)
C(34)-C(33)-H(33)	121(2)	N(8)-C(34)-C(40)	124.2(2)
N(8)-C(34)-C(33)	117.1(2)	C(40)-C(34)-C(33)	118.7(2)
N(7)-C(35)-C(36)	117.3(2)	N(7)-C(35)-C(39)	124.2(2)
C(36)-C(35)-C(39)	118.4(2)	C(37)-C(36)-C(35)	118.3(2)
C(37)-C(36)-H(36)	122(2)	C(35)-C(36)-H(36)	120(2)
C(36)-C(37)-C(38)	118.8(3)	C(36)-C(37)-H(37)	126(2)
C(38)-C(37)-H(37)	115(2)	N(6)-C(38)-C(37)	124.8(3)
N(6)-C(38)-H(38)	117(2)	C(37)-C(38)-H(38)	118(2)
N(6)-C(39)-C(35)	123.1(2)	N(6)-C(39)-C(40)	121.2(2)
C(35)-C(39)-C(40)	115.7(2)	N(5)-C(40)-C(34)	123.4(2)
N(5)-C(40)-C(39)	120.6(2)	C(34)-C(40)-C(39)	116.0(2)
Cl(2)-C(41)-Cl(1)	110.7(2)	Cl(2)-C(41)-H(41A)	110(2)
Cl(1)-C(41)-H(41A)	105(2)	Cl(2)-C(41)-H(41B)	108(2)
Cl(1)-C(41)-H(41B)	109(2)	H(41A)-C(41)-H(41B)	114(3)

Table S9. Bond distances (Å) and Angle (deg) for **2**.

Pt(1)-C(12)	2.015(7)	Pt(1)-C(11)	2.050(7)
Pt(1)-N(1)	2.100(5)	Pt(1)-N(2)	2.111(5)
N(1)-C(10)	1.35(1)	N(1)-C(1)	1.36(1)
N(2)-C(3)	1.32(1)	N(2)-C(2)	1.36(1)
N(3)-N(4)	1.30(1)	N(3)-C(6)	1.40(1)
N(4)-C(7)	1.39(1)	C(1)-C(7)	1.38(1)
C(1)-C(2)	1.42(1)	C(2)-C(6)	1.38(1)
C(3)-C(4)	1.41(1)	C(3)-H(3)	0.9500
C(4)-C(5)	1.35(1)	C(4)-H(4)	0.9500
C(5)-C(6)	1.39(1)	C(5)-H(5)	0.9500
C(7)-C(8)	1.40(1)	C(8)-C(9)	1.37(2)
C(8)-H(8)	0.9500	C(9)-C(10)	1.39(1)
C(9)-H(9)	0.9500	C(10)-H(10)	0.9500
C(11)-H(11A)	0.9800	C(11)-H(11B)	0.9800
C(11)-H(11C)	0.9800	C(12)-H(12A)	0.9800
C(12)-H(12B)	0.9800	C(12)-H(12C)	0.9800

C(12)-Pt(1)-C(11)	87.2(3)	C(12)-Pt(1)-N(1)	176.5(3)
C(11)-Pt(1)-N(1)	96.2(3)	C(12)-Pt(1)-N(2)	96.8(3)
C(11)-Pt(1)-N(2)	175.9(3)	N(1)-Pt(1)-N(2)	79.7(2)
C(10)-N(1)-C(1)	116.3(6)	C(10)-N(1)-Pt(1)	131.2(5)
C(1)-N(1)-Pt(1)	112.5(5)	C(3)-N(2)-C(2)	116.2(6)
C(3)-N(2)-Pt(1)	132.2(5)	C(2)-N(2)-Pt(1)	111.6(4)
N(4)-N(3)-C(6)	120.7(7)	N(3)-N(4)-C(7)	120.2(7)
N(1)-C(1)-C(7)	124.1(7)	N(1)-C(1)-C(2)	117.5(6)
C(7)-C(1)-C(2)	118.5(7)	N(2)-C(2)-C(6)	124.2(7)
N(2)-C(2)-C(1)	118.7(6)	C(6)-C(2)-C(1)	117.1(7)
N(2)-C(3)-C(4)	122.5(8)	N(2)-C(3)-H(3)	118.8
C(4)-C(3)-H(3)	118.8	C(5)-C(4)-C(3)	121(1)
C(5)-C(4)-H(4)	119.6	C(3)-C(4)-H(4)	119.6
C(4)-C(5)-C(6)	117.9(8)	C(4)-C(5)-H(5)	121.0
C(6)-C(5)-H(5)	121.0	C(2)-C(6)-C(5)	118.5(8)
C(2)-C(6)-N(3)	121.8(8)	C(5)-C(6)-N(3)	119.7(8)
C(1)-C(7)-N(4)	121.7(8)	C(1)-C(7)-C(8)	118.6(8)
N(4)-C(7)-C(8)	119.6(8)	C(9)-C(8)-C(7)	117.1(8)
C(9)-C(8)-H(8)	121.4	C(7)-C(8)-H(8)	121.4
C(8)-C(9)-C(10)	122(1)	C(8)-C(9)-H(9)	119.1
C(10)-C(9)-H(9)	119.1	N(1)-C(10)-C(9)	122(1)
N(1)-C(10)-H(10)	119.0	C(9)-C(10)-H(10)	119.0
Pt(1)-C(11)-H(11A)	109.5	Pt(1)-C(11)-H(11B)	109.5
H(11A)-C(11)-H(11B)	109.5	Pt(1)-C(11)-H(11C)	109.5
H(11A)-C(11)-H(11C)	109.5	H(11B)-C(11)-H(11C)	109.5
Pt(1)-C(12)-H(12A)	109.5	Pt(1)-C(12)-H(12B)	109.5
H(12A)-C(12)-H(12B)	109.5	Pt(1)-C(12)-H(12C)	109.5
H(12A)-C(12)-H(12C)	109.5	H(12B)-C(12)-H(12C)	109.5

Table S10. Bond distances (Å) and Angle (deg) for **4**.

Pt(1)-C(31)	2.03(2)	Pt(1)-N(1)	2.08(2)
Pt(1)-N(2)	2.10(1)	Pt(1)-C(32)	2.10(3)
Yb(1)-N(3)	2.26(1)	Yb(1)-N(4)	2.28(1)
Yb(1)-C(21)	2.55(1)	Yb(1)-C(24)	2.56(1)
Yb(1)-C(15)	2.56(2)	Yb(1)-C(25)	2.56(2)
Yb(1)-C(11)	2.58(1)	Yb(1)-C(23)	2.58(2)
Yb(1)-C(14)	2.58(2)	Yb(1)-C(12)	2.59(2)
Yb(1)-C(13)	2.60(2)	Yb(1)-C(22)	2.61(2)
N(1)-C(1)	1.36(2)	N(1)-C(10)	1.39(3)
N(2)-C(2)	1.34(2)	N(2)-C(3)	1.39(2)
N(3)-N(4)	1.37(2)	N(3)-C(6)	1.38(2)
N(4)-C(7)	1.37(2)	C(1)-C(7)	1.41(3)
C(1)-C(2)	1.46(3)	C(2)-C(6)	1.37(2)
C(3)-C(4)	1.36(3)	C(3)-H(36)	0.9500
C(4)-C(5)	1.40(2)	C(4)-H(35)	0.9500
C(5)-C(6)	1.39(2)	C(5)-H(34)	0.9500
C(7)-C(8)	1.40(3)	C(8)-C(9)	1.36(3)
C(8)-H(3)	0.9500	C(9)-C(10)	1.39(4)
C(9)-H(2)	0.9500	C(10)-H(1)	0.9500
C(11)-C(12)	1.38(2)	C(11)-C(15)	1.41(2)
C(11)-C(20)	1.48(2)	C(12)-C(13)	1.40(2)
C(12)-C(19)	1.53(3)	C(13)-C(14)	1.39(3)
C(13)-C(18)	1.60(3)	C(14)-C(15)	1.39(3)
C(14)-C(17)	1.54(2)	C(15)-C(16)	1.51(3)
C(16)-H(4)	0.9800	C(16)-H(5)	0.9800
C(16)-H(6)	0.9800	C(17)-H(7)	0.9800
C(17)-H(8)	0.9800	C(17)-H(9)	0.9800
C(18)-H(10)	0.9800	C(18)-H(11)	0.9800
C(18)-H(12)	0.9800	C(19)-H(13)	0.9800
C(19)-H(14)	0.9800	C(19)-H(15)	0.9800
C(20)-H(16)	0.9800	C(20)-H(17)	0.9800
C(20)-H(18)	0.9800	C(21)-C(22)	1.39(2)
C(21)-C(25)	1.45(2)	C(21)-C(30)	1.51(2)
C(22)-C(23)	1.37(2)	C(22)-C(29)	1.51(2)
C(23)-C(24)	1.40(3)	C(23)-C(28)	1.53(3)
C(24)-C(25)	1.45(3)	C(24)-C(27)	1.56(2)
C(25)-C(26)	1.49(3)	C(26)-H(19)	0.9800
C(26)-H(20)	0.9800	C(26)-H(21)	0.9800
C(27)-H(22)	0.9800	C(27)-H(23)	0.9800
C(27)-H(24)	0.9800	C(28)-H(25)	0.9800
C(28)-H(26)	0.9800	C(28)-H(27)	0.9800
C(29)-H(28)	0.9800	C(29)-H(29)	0.9800
C(29)-H(30)	0.9800	C(30)-H(31)	0.9800
C(30)-H(32)	0.9800	C(30)-H(33)	0.9800
C(31)-H(37)	0.9800	C(31)-H(38)	0.9800
C(31)-H(39)	0.9800	C(32)-H(40)	0.9800
C(32)-H(41)	0.9800	C(32)-H(42)	0.9800
Pt(2)-C(63)	1.99(1)	Pt(2)-C(64)	2.05(2)
Pt(2)-N(5)	2.11(1)	Pt(2)-N(6)	2.12(1)
Yb(2)-N(8)	2.30(1)	Yb(2)-N(7)	2.30(1)
Yb(2)-C(57)	2.55(2)	Yb(2)-C(53)	2.55(1)
Yb(2)-C(45)	2.57(1)	Yb(2)-C(56)	2.57(2)
Yb(2)-C(47)	2.58(2)	Yb(2)-C(46)	2.58(1)
Yb(2)-C(55)	2.60(2)	Yb(2)-C(44)	2.60(1)
Yb(2)-C(54)	2.60(2)	Yb(2)-C(43)	2.61(2)
N(5)-C(33)	1.35(2)	N(5)-C(42)	1.36(2)
N(6)-C(35)	1.32(2)	N(6)-C(34)	1.37(2)
N(7)-C(38)	1.32(2)	N(7)-N(8)	1.39(2)
N(8)-C(39)	1.35(2)	C(33)-C(39)	1.42(2)
C(33)-C(34)	1.42(2)	C(34)-C(38)	1.43(2)
C(35)-C(36)	1.40(2)	C(35)-H(78)	0.9500
C(36)-C(37)	1.39(2)	C(36)-H(77)	0.9500
C(37)-C(38)	1.40(2)	C(37)-H(76)	0.9500
C(39)-C(40)	1.42(2)	C(40)-C(41)	1.35(2)
C(40)-H(45)	0.9500	C(41)-C(42)	1.39(2)
C(41)-H(44)	0.9500	C(42)-H(43)	0.9500
C(43)-C(44)	1.39(2)	C(43)-C(47)	1.44(3)
C(43)-C(52)	1.47(3)	C(44)-C(45)	1.41(2)
C(44)-C(51)	1.53(2)	C(45)-C(46)	1.41(2)
C(45)-C(50)	1.52(2)	C(46)-C(47)	1.41(2)
C(46)-C(49)	1.48(2)	C(47)-C(48)	1.51(2)
C(48)-H(46)	0.9800	C(48)-H(47)	0.9800
C(48)-H(48)	0.9800	C(49)-H(49)	0.9800
C(49)-H(50)	0.9800	C(49)-H(51)	0.9800
C(50)-H(52)	0.9800	C(50)-H(53)	0.9800
C(50)-H(54)	0.9800	C(51)-H(55)	0.9800

Table S10. Bond distances (Å) and Angle (deg) for **4** (continued).

C(51)-H(56)	0.9800	C(51)-H(57)	0.9800
C(52)-H(58)	0.9800	C(52)-H(59)	0.9800
C(52)-H(60)	0.9800	C(53)-C(57)	1.40(3)
C(53)-C(54)	1.46(2)	C(53)-C(62)	1.49(3)
C(54)-C(55)	1.41(3)	C(54)-C(61)	1.53(2)
C(55)-C(56)	1.38(3)	C(55)-C(60)	1.51(3)
C(56)-C(57)	1.40(3)	C(56)-C(59)	1.52(3)
C(57)-C(58)	1.46(3)	C(58)-H(61)	0.9800
C(58)-H(62)	0.9800	C(58)-H(63)	0.9800
C(59)-H(64)	0.9800	C(59)-H(65)	0.9800
C(59)-H(66)	0.9800	C(60)-H(67)	0.9800
C(60)-H(68)	0.9800	C(60)-H(69)	0.9800
C(61)-H(70)	0.9800	C(61)-H(71)	0.9800
C(61)-H(72)	0.9800	C(62)-H(73)	0.9800
C(62)-H(74)	0.9800	C(62)-H(75)	0.9800
C(63)-H(79)	0.9800	C(63)-H(80)	0.9800
C(63)-H(81)	0.9800	C(64)-H(82)	0.9800
C(64)-H(83)	0.9800	C(64)-H(84)	0.9800

C(31)-Pt(1)-N(1)	94(1)	C(31)-Pt(1)-N(2)	174(1)
N(1)-Pt(1)-N(2)	79.4(5)	C(31)-Pt(1)-C(32)	93(1)
N(1)-Pt(1)-C(32)	172.5(8)	N(2)-Pt(1)-C(32)	93.1(8)
N(3)-Yb(1)-N(4)	35.1(5)	N(3)-Yb(1)-C(21)	105.6(5)
N(4)-Yb(1)-C(21)	91.3(5)	N(3)-Yb(1)-C(24)	117.5(6)
N(4)-Yb(1)-C(24)	131.9(6)	C(21)-Yb(1)-C(24)	52.6(5)
N(3)-Yb(1)-C(15)	81.9(5)	N(4)-Yb(1)-C(15)	90.7(5)
C(21)-Yb(1)-C(15)	169.3(6)	C(24)-Yb(1)-C(15)	131.0(6)
N(3)-Yb(1)-C(25)	133.9(5)	N(4)-Yb(1)-C(25)	124.2(5)
C(21)-Yb(1)-C(25)	33.0(5)	C(24)-Yb(1)-C(25)	32.7(6)
C(15)-Yb(1)-C(25)	142.7(5)	N(3)-Yb(1)-C(11)	106.1(5)
N(4)-Yb(1)-C(11)	122.4(5)	C(21)-Yb(1)-C(11)	145.7(5)
C(24)-Yb(1)-C(11)	100.4(5)	C(15)-Yb(1)-C(11)	31.9(5)
C(25)-Yb(1)-C(11)	112.8(5)	N(3)-Yb(1)-C(23)	86.5(6)
N(4)-Yb(1)-C(23)	102.7(5)	C(21)-Yb(1)-C(23)	51.9(5)
C(24)-Yb(1)-C(23)	31.7(6)	C(15)-Yb(1)-C(23)	137.5(6)
C(25)-Yb(1)-C(23)	53.9(6)	C(11)-Yb(1)-C(23)	117.9(5)
N(3)-Yb(1)-C(14)	91.3(6)	N(4)-Yb(1)-C(14)	81.5(5)
C(21)-Yb(1)-C(14)	139.0(7)	C(24)-Yb(1)-C(14)	146.4(6)
C(15)-Yb(1)-C(14)	31.3(6)	C(25)-Yb(1)-C(14)	132.1(7)
C(11)-Yb(1)-C(14)	52.2(5)	C(23)-Yb(1)-C(14)	168.7(7)
N(3)-Yb(1)-C(12)	133.5(5)	N(4)-Yb(1)-C(12)	133.2(5)
C(21)-Yb(1)-C(12)	120.8(5)	C(24)-Yb(1)-C(12)	94.6(6)
C(15)-Yb(1)-C(12)	51.7(5)	C(25)-Yb(1)-C(12)	91.5(6)
C(11)-Yb(1)-C(12)	31.0(5)	C(23)-Yb(1)-C(12)	123.7(6)
C(14)-Yb(1)-C(12)	51.8(6)	N(3)-Yb(1)-C(13)	122.3(6)
N(4)-Yb(1)-C(13)	105.4(6)	C(21)-Yb(1)-C(13)	117.8(6)
C(24)-Yb(1)-C(13)	118.7(7)	C(15)-Yb(1)-C(13)	51.6(6)
C(25)-Yb(1)-C(13)	101.4(6)	C(11)-Yb(1)-C(13)	51.8(5)
C(23)-Yb(1)-C(13)	150.4(7)	C(14)-Yb(1)-C(13)	31.2(7)
C(12)-Yb(1)-C(13)	31.4(5)	N(3)-Yb(1)-C(22)	80.6(5)
N(4)-Yb(1)-C(22)	81.0(5)	C(21)-Yb(1)-C(22)	31.1(5)
C(24)-Yb(1)-C(22)	51.4(6)	C(15)-Yb(1)-C(22)	159.4(6)
C(25)-Yb(1)-C(22)	53.3(6)	C(11)-Yb(1)-C(22)	148.3(5)
C(23)-Yb(1)-C(22)	30.5(5)	C(14)-Yb(1)-C(22)	159.4(5)
C(12)-Yb(1)-C(22)	143.6(5)	C(13)-Yb(1)-C(22)	148.9(6)
C(1)-N(1)-C(10)	115(2)	C(1)-N(1)-Pt(1)	113(2)
C(10)-N(1)-Pt(1)	132(1)	C(2)-N(2)-C(3)	116(2)
C(2)-N(2)-Pt(1)	114(1)	C(3)-N(2)-Pt(1)	131(1)
N(4)-N(3)-C(6)	121(1)	N(4)-N(3)-Yb(1)	73.3(8)
C(6)-N(3)-Yb(1)	165(1)	C(7)-N(4)-N(3)	121(2)
C(7)-N(4)-Yb(1)	167(1)	N(3)-N(4)-Yb(1)	71.6(8)
N(1)-C(1)-C(7)	124(2)	N(1)-C(1)-C(2)	117(2)
C(7)-C(1)-C(2)	119(2)	N(2)-C(2)-C(6)	125(2)
N(2)-C(2)-C(1)	117(2)	C(6)-C(2)-C(1)	118(2)
C(4)-C(3)-N(2)	122(2)	C(4)-C(3)-H(36)	118.8
N(2)-C(3)-H(36)	118.8	C(3)-C(4)-C(5)	120(2)
C(3)-C(4)-H(35)	119.8	C(5)-C(4)-H(35)	119.8
C(6)-C(5)-C(4)	118(2)	C(6)-C(5)-H(34)	121.3
C(4)-C(5)-H(34)	121.3	C(2)-C(6)-N(3)	121(1)
C(2)-C(6)-C(5)	119(2)	N(3)-C(6)-C(5)	120(1)
N(4)-C(7)-C(8)	122(2)	N(4)-C(7)-C(1)	120(2)
C(8)-C(7)-C(1)	118(2)	C(9)-C(8)-C(7)	119(2)
C(9)-C(8)-H(3)	120.6	C(7)-C(8)-H(3)	120.6
C(8)-C(9)-C(10)	121(2)	C(8)-C(9)-H(2)	119.6
C(10)-C(9)-H(2)	119.6	C(9)-C(10)-N(1)	123(2)
C(9)-C(10)-H(1)	118.4	N(1)-C(10)-H(1)	118.4

Table S10. Bond distances (Å) and Angle (deg) for **4** (continued).

C(12)-C(11)-C(15)	107(1)	C(12)-C(11)-C(20)	129(2)
C(15)-C(11)-C(20)	124(2)	C(12)-C(11)-Yb(1)	75(1)
C(15)-C(11)-Yb(1)	74(1)	C(20)-C(11)-Yb(1)	118(1)
C(11)-C(12)-C(13)	109(2)	C(11)-C(12)-C(19)	126(2)
C(13)-C(12)-C(19)	124(2)	C(11)-C(12)-Yb(1)	74(1)
C(13)-C(12)-Yb(1)	75(1)	C(19)-C(12)-Yb(1)	127(1)
C(14)-C(13)-C(12)	108(2)	C(14)-C(13)-C(18)	130(2)
C(12)-C(13)-C(18)	122(2)	C(14)-C(13)-Yb(1)	74(1)
C(12)-C(13)-Yb(1)	74(1)	C(18)-C(13)-Yb(1)	122(1)
C(15)-C(14)-C(13)	108(2)	C(15)-C(14)-C(17)	128(2)
C(13)-C(14)-C(17)	124(2)	C(15)-C(14)-Yb(1)	74(1)
C(13)-C(14)-Yb(1)	75(1)	C(17)-C(14)-Yb(1)	120(1)
C(14)-C(15)-C(11)	108(2)	C(14)-C(15)-C(16)	124(2)
C(11)-C(15)-C(16)	128(2)	C(14)-C(15)-Yb(1)	75(1)
C(11)-C(15)-Yb(1)	75(1)	C(16)-C(15)-Yb(1)	120(1)
C(15)-C(16)-H(4)	109.5	C(15)-C(16)-H(5)	109.5
H(4)-C(16)-H(5)	109.5	C(15)-C(16)-H(6)	109.5
H(4)-C(16)-H(6)	109.5	H(5)-C(16)-H(6)	109.5
C(14)-C(17)-H(7)	109.5	C(14)-C(17)-H(8)	109.5
H(7)-C(17)-H(8)	109.5	C(14)-C(17)-H(9)	109.5
H(7)-C(17)-H(9)	109.5	H(8)-C(17)-H(9)	109.5
C(13)-C(18)-H(10)	109.5	C(13)-C(18)-H(11)	109.5
H(10)-C(18)-H(11)	109.5	C(13)-C(18)-H(12)	109.5
H(10)-C(18)-H(12)	109.5	H(11)-C(18)-H(12)	109.5
C(12)-C(19)-H(13)	109.5	C(12)-C(19)-H(14)	109.5
H(13)-C(19)-H(14)	109.5	C(12)-C(19)-H(15)	109.5
H(13)-C(19)-H(15)	109.5	H(14)-C(19)-H(15)	109.5
C(11)-C(20)-H(16)	109.5	C(11)-C(20)-H(17)	109.5
H(16)-C(20)-H(17)	109.5	C(11)-C(20)-H(18)	109.5
H(16)-C(20)-H(18)	109.5	H(17)-C(20)-H(18)	109.5
C(22)-C(21)-C(25)	110(1)	C(22)-C(21)-C(30)	126(2)
C(25)-C(21)-C(30)	124(2)	C(22)-C(21)-Yb(1)	77(1)
C(25)-C(21)-Yb(1)	74(1)	C(30)-C(21)-Yb(1)	118(1)
C(23)-C(22)-C(21)	110(2)	C(23)-C(22)-C(29)	124(2)
C(21)-C(22)-C(29)	127(2)	C(23)-C(22)-Yb(1)	74(1)
C(21)-C(22)-Yb(1)	72(1)	C(29)-C(22)-Yb(1)	122(1)
C(22)-C(23)-C(24)	108(2)	C(22)-C(23)-C(28)	127(2)
C(24)-C(23)-C(28)	124(2)	C(22)-C(23)-Yb(1)	76(1)
C(24)-C(23)-Yb(1)	74(1)	C(28)-C(23)-Yb(1)	121(1)
C(23)-C(24)-C(25)	110(2)	C(23)-C(24)-C(27)	131(2)
C(25)-C(24)-C(27)	119(2)	C(23)-C(24)-Yb(1)	75(1)
C(25)-C(24)-Yb(1)	74(1)	C(27)-C(24)-Yb(1)	123(1)
C(24)-C(25)-C(21)	103(2)	C(24)-C(25)-C(26)	131(2)
C(21)-C(25)-C(26)	125(2)	C(24)-C(25)-Yb(1)	74(1)
C(21)-C(25)-Yb(1)	73(1)	C(26)-C(25)-Yb(1)	126(1)
C(25)-C(26)-H(19)	109.5	C(25)-C(26)-H(20)	109.5
H(19)-C(26)-H(20)	109.5	C(25)-C(26)-H(21)	109.5
H(19)-C(26)-H(21)	109.5	H(20)-C(26)-H(21)	109.5
C(24)-C(27)-H(22)	109.5	C(24)-C(27)-H(23)	109.5
H(22)-C(27)-H(23)	109.5	C(24)-C(27)-H(24)	109.5
H(22)-C(27)-H(24)	109.5	H(23)-C(27)-H(24)	109.5
C(23)-C(28)-H(25)	109.5	C(23)-C(28)-H(26)	109.5
H(25)-C(28)-H(26)	109.5	C(23)-C(28)-H(27)	109.5
H(25)-C(28)-H(27)	109.5	H(26)-C(28)-H(27)	109.5
C(22)-C(29)-H(28)	109.5	C(22)-C(29)-H(29)	109.5
H(28)-C(29)-H(29)	109.5	C(22)-C(29)-H(30)	109.5
H(28)-C(29)-H(30)	109.5	H(29)-C(29)-H(30)	109.5
C(21)-C(30)-H(31)	109.5	C(21)-C(30)-H(32)	109.5
H(31)-C(30)-H(32)	109.5	C(21)-C(30)-H(33)	109.5
H(31)-C(30)-H(33)	109.5	H(32)-C(30)-H(33)	109.5
Pt(1)-C(31)-H(37)	109.5	Pt(1)-C(31)-H(38)	109.5
H(37)-C(31)-H(38)	109.5	Pt(1)-C(31)-H(39)	109.5
H(37)-C(31)-H(39)	109.5	H(38)-C(31)-H(39)	109.5
Pt(1)-C(32)-H(40)	109.5	Pt(1)-C(32)-H(41)	109.5
H(40)-C(32)-H(41)	109.5	Pt(1)-C(32)-H(42)	109.5
H(40)-C(32)-H(42)	109.5	H(41)-C(32)-H(42)	109.5
C(63)-Pt(2)-C(64)	91.9(7)	C(63)-Pt(2)-N(5)	172.2(5)
C(64)-Pt(2)-N(5)	94.8(6)	C(63)-Pt(2)-N(6)	93.4(6)
C(64)-Pt(2)-N(6)	174.6(6)	N(5)-Pt(2)-N(6)	80.0(5)
N(8)-Yb(2)-N(7)	35.1(4)	N(8)-Yb(2)-C(57)	80.0(5)
N(7)-Yb(2)-C(57)	83.3(5)	N(8)-Yb(2)-C(53)	103.8(6)
N(7)-Yb(2)-C(53)	90.8(5)	C(57)-Yb(2)-C(53)	32.0(6)
N(8)-Yb(2)-C(45)	103.9(5)	N(7)-Yb(2)-C(45)	119.8(4)
C(57)-Yb(2)-C(45)	145.5(6)	C(53)-Yb(2)-C(45)	149.2(5)
N(8)-Yb(2)-C(56)	91.1(5)	N(7)-Yb(2)-C(56)	109.0(5)
C(57)-Yb(2)-C(56)	31.8(6)	C(53)-Yb(2)-C(56)	52.4(6)
C(45)-Yb(2)-C(56)	113.9(6)	N(8)-Yb(2)-C(47)	94.4(5)

Table S10. Bond distances (Å) and Angle (deg) for **4** (continued).

N(7)-Yb(2)-C(47)	82.1(5)	C(57)-Yb(2)-C(47)	161.4(6)
C(53)-Yb(2)-C(47)	136.9(6)	C(45)-Yb(2)-C(47)	53.0(5)
C(56)-Yb(2)-C(47)	166.8(6)	N(8)-Yb(2)-C(46)	82.3(5)
N(7)-Yb(2)-C(46)	88.8(4)	C(57)-Yb(2)-C(46)	158.9(5)
C(53)-Yb(2)-C(46)	168.5(6)	C(45)-Yb(2)-C(46)	31.8(5)
C(56)-Yb(2)-C(46)	138.2(6)	C(47)-Yb(2)-C(46)	31.8(5)
N(8)-Yb(2)-C(55)	122.0(6)	N(7)-Yb(2)-C(55)	136.1(5)
C(57)-Yb(2)-C(55)	52.9(6)	C(53)-Yb(2)-C(55)	53.2(5)
C(45)-Yb(2)-C(55)	99.9(5)	C(56)-Yb(2)-C(55)	31.1(6)
C(47)-Yb(2)-C(55)	140.6(6)	C(46)-Yb(2)-C(55)	131.7(5)
N(8)-Yb(2)-C(44)	133.5(5)	N(7)-Yb(2)-C(44)	134.3(5)
C(57)-Yb(2)-C(44)	141.7(5)	C(53)-Yb(2)-C(44)	122.7(6)
C(45)-Yb(2)-C(44)	31.8(5)	C(56)-Yb(2)-C(44)	116.1(6)
C(47)-Yb(2)-C(44)	52.2(5)	C(46)-Yb(2)-C(44)	52.0(5)
C(55)-Yb(2)-C(44)	89.3(6)	N(8)-Yb(2)-C(54)	133.2(5)
N(7)-Yb(2)-C(54)	123.3(5)	C(57)-Yb(2)-C(54)	53.4(5)
C(53)-Yb(2)-C(54)	33.0(6)	C(45)-Yb(2)-C(54)	116.2(5)
C(56)-Yb(2)-C(54)	51.9(6)	C(47)-Yb(2)-C(54)	128.3(6)
C(46)-Yb(2)-C(54)	143.9(5)	C(55)-Yb(2)-C(54)	31.6(6)
C(44)-Yb(2)-C(54)	91.9(5)	N(8)-Yb(2)-C(43)	126.6(5)
N(7)-Yb(2)-C(43)	108.8(5)	C(57)-Yb(2)-C(43)	148.5(5)
C(53)-Yb(2)-C(43)	117.0(6)	C(45)-Yb(2)-C(43)	52.8(5)
C(56)-Yb(2)-C(43)	140.8(6)	C(47)-Yb(2)-C(43)	32.2(6)
C(46)-Yb(2)-C(43)	52.6(5)	C(55)-Yb(2)-C(43)	110.0(6)
C(44)-Yb(2)-C(43)	31.1(5)	C(54)-Yb(2)-C(43)	97.5(5)
C(33)-N(5)-C(42)	119(1)	C(33)-N(5)-Pt(2)	112(1)
C(42)-N(5)-Pt(2)	130(1)	C(35)-N(6)-C(34)	118(1)
C(35)-N(6)-Pt(2)	131(1)	C(34)-N(6)-Pt(2)	111(1)
C(38)-N(7)-N(8)	121(1)	C(38)-N(7)-Yb(2)	166(1)
N(8)-N(7)-Yb(2)	72.3(7)	C(39)-N(8)-N(7)	121(1)
C(39)-N(8)-Yb(2)	166(1)	N(7)-N(8)-Yb(2)	72.6(7)
N(5)-C(33)-C(39)	123(1)	N(5)-C(33)-C(34)	119(1)
C(39)-C(33)-C(34)	118(1)	N(6)-C(34)-C(33)	119(1)
N(6)-C(34)-C(38)	123(1)	C(33)-C(34)-C(38)	118(1)
N(6)-C(35)-C(36)	122(1)	N(6)-C(35)-H(78)	118.9
C(36)-C(35)-H(78)	118.9	C(37)-C(36)-C(35)	121(1)
C(37)-C(36)-H(77)	119.7	C(35)-C(36)-H(77)	119.7
C(36)-C(37)-C(38)	119(1)	C(36)-C(37)-H(76)	120.6
C(38)-C(37)-H(76)	120.6	N(7)-C(38)-C(37)	122(1)
N(7)-C(38)-C(34)	121(1)	C(37)-C(38)-C(34)	117(1)
N(8)-C(39)-C(33)	121(1)	N(8)-C(39)-C(40)	122(1)
C(33)-C(39)-C(40)	117(1)	C(41)-C(40)-C(39)	118(1)
C(41)-C(40)-H(45)	121.1	C(39)-C(40)-H(45)	121.1
C(40)-C(41)-C(42)	124(2)	C(40)-C(41)-H(44)	118.3
C(42)-C(41)-H(44)	118.3	N(5)-C(42)-C(41)	120(1)
N(5)-C(42)-H(43)	120.2	C(41)-C(42)-H(43)	120.2
C(44)-C(43)-C(47)	107(2)	C(44)-C(43)-C(52)	128(2)
C(47)-C(43)-C(52)	125(2)	C(44)-C(43)-Yb(2)	74(1)
C(47)-C(43)-Yb(2)	73(1)	C(52)-C(43)-Yb(2)	121(1)
C(43)-C(44)-C(45)	110(1)	C(43)-C(44)-C(51)	125(2)
C(45)-C(44)-C(51)	124(2)	C(43)-C(44)-Yb(2)	75(1)
C(45)-C(44)-Yb(2)	72.9(8)	C(51)-C(44)-Yb(2)	128(1)
C(46)-C(45)-C(44)	107(1)	C(46)-C(45)-C(50)	126(2)
C(44)-C(45)-C(50)	128(1)	C(46)-C(45)-Yb(2)	74.6(8)
C(44)-C(45)-Yb(2)	75.3(8)	C(50)-C(45)-Yb(2)	119(1)
C(45)-C(46)-C(47)	109(1)	C(45)-C(46)-C(49)	126(2)
C(47)-C(46)-C(49)	125(2)	C(45)-C(46)-Yb(2)	73.6(8)
C(47)-C(46)-Yb(2)	74(1)	C(49)-C(46)-Yb(2)	120(1)
C(46)-C(47)-C(43)	107(1)	C(46)-C(47)-C(48)	126(2)
C(43)-C(47)-C(48)	127(2)	C(46)-C(47)-Yb(2)	74(1)
C(43)-C(47)-Yb(2)	75(1)	C(48)-C(47)-Yb(2)	121(1)
C(47)-C(48)-H(46)	109.5	C(47)-C(48)-H(47)	109.5
H(46)-C(48)-H(47)	109.5	C(47)-C(48)-H(48)	109.5
H(46)-C(48)-H(48)	109.5	H(47)-C(48)-H(48)	109.5
C(46)-C(49)-H(49)	109.5	C(46)-C(49)-H(50)	109.5
H(49)-C(49)-H(50)	109.5	C(46)-C(49)-H(51)	109.5
H(49)-C(49)-H(51)	109.5	H(50)-C(49)-H(51)	109.5
C(45)-C(50)-H(52)	109.5	C(45)-C(50)-H(53)	109.5
H(52)-C(50)-H(53)	109.5	C(45)-C(50)-H(54)	109.5
H(52)-C(50)-H(54)	109.5	H(53)-C(50)-H(54)	109.5
C(44)-C(51)-H(55)	109.5	C(44)-C(51)-H(56)	109.5
H(55)-C(51)-H(56)	109.5	C(44)-C(51)-H(57)	109.5
H(55)-C(51)-H(57)	109.5	H(56)-C(51)-H(57)	109.5
C(43)-C(52)-H(58)	109.5	C(43)-C(52)-H(59)	109.5
H(58)-C(52)-H(59)	109.5	C(43)-C(52)-H(60)	109.5
H(58)-C(52)-H(60)	109.5	H(59)-C(52)-H(60)	109.5
C(57)-C(53)-C(54)	107(2)	C(57)-C(53)-C(62)	125(2)

Table S10. Bond distances (Å) and Angle (deg) for **4** (continued).

C(54)-C(53)-C(62)	128(2)	C(57)-C(53)-Yb(2)	74(1)
C(54)-C(53)-Yb(2)	75(1)	C(62)-C(53)-Yb(2)	117(1)
C(55)-C(54)-C(53)	107(2)	C(55)-C(54)-C(61)	132(2)
C(53)-C(54)-C(61)	122(2)	C(55)-C(54)-Yb(2)	74(1)
C(53)-C(54)-Yb(2)	72(1)	C(61)-C(54)-Yb(2)	122(1)
C(56)-C(55)-C(54)	108(2)	C(56)-C(55)-C(60)	128(2)
C(54)-C(55)-C(60)	123(2)	C(56)-C(55)-Yb(2)	73(1)
C(54)-C(55)-Yb(2)	74(1)	C(60)-C(55)-Yb(2)	126(1)
C(55)-C(56)-C(57)	111(2)	C(55)-C(56)-C(59)	125(2)
C(57)-C(56)-C(59)	124(2)	C(55)-C(56)-Yb(2)	76(1)
C(57)-C(56)-Yb(2)	73(1)	C(59)-C(56)-Yb(2)	119(1)
C(56)-C(57)-C(53)	107(1)	C(56)-C(57)-C(58)	128(2)
C(53)-C(57)-C(58)	124(2)	C(56)-C(57)-Yb(2)	75(1)
C(53)-C(57)-Yb(2)	74(1)	C(58)-C(57)-Yb(2)	122(1)
C(57)-C(58)-H(61)	109.5	C(57)-C(58)-H(62)	109.5
H(61)-C(58)-H(62)	109.5	C(57)-C(58)-H(63)	109.5
H(61)-C(58)-H(63)	109.5	H(62)-C(58)-H(63)	109.5
C(56)-C(59)-H(64)	109.5	C(56)-C(59)-H(65)	109.5
H(64)-C(59)-H(65)	109.5	C(56)-C(59)-H(66)	109.5
H(64)-C(59)-H(66)	109.5	H(65)-C(59)-H(66)	109.5
C(55)-C(60)-H(67)	109.5	C(55)-C(60)-H(68)	109.5
H(67)-C(60)-H(68)	109.5	C(55)-C(60)-H(69)	109.5
H(67)-C(60)-H(69)	109.5	H(68)-C(60)-H(69)	109.5
C(54)-C(61)-H(70)	109.5	C(54)-C(61)-H(71)	109.5
H(70)-C(61)-H(71)	109.5	C(54)-C(61)-H(72)	109.5
H(70)-C(61)-H(72)	109.5	H(71)-C(61)-H(72)	109.5
C(53)-C(62)-H(73)	109.5	C(53)-C(62)-H(74)	109.5
H(73)-C(62)-H(74)	109.5	C(53)-C(62)-H(75)	109.5
H(73)-C(62)-H(75)	109.5	H(74)-C(62)-H(75)	109.5
Pt(2)-C(63)-H(79)	109.5	Pt(2)-C(63)-H(80)	109.5
H(79)-C(63)-H(80)	109.5	Pt(2)-C(63)-H(81)	109.5
H(79)-C(63)-H(81)	109.5	H(80)-C(63)-H(81)	109.5
Pt(2)-C(64)-H(82)	109.5	Pt(2)-C(64)-H(83)	109.5
H(82)-C(64)-H(83)	109.5	Pt(2)-C(64)-H(84)	109.5
H(82)-C(64)-H(84)	109.5	H(83)-C(64)-H(84)	109.5

Table S11. Bond distances (Å) and Angle (deg) for **5**.

Yb(01)-C(52)	2.561(8)	Yb(01)-C(23)	2.57(1)
Yb(01)-C(51)	2.57(1)	Yb(01)-C(22)	2.60(1)
Yb(01)-C(21)	2.62(1)	Yb(01)-C(24)	2.63(1)
Yb(01)-C(35)	2.640(8)	Yb(01)-C(25)	2.64(1)
Yb(01)-C(33)	2.64(1)	Yb(01)-C(31)	2.65(1)
Yb(01)-C(32)	2.652(8)	Yb(01)-C(34)	2.65(1)
Yb(01)-H(51A)	2.3(1)	Yb(01)-H(52B)	2.3854
Yb(01)-H(52C)	2.4268	Yb(02)-N(4)	2.199(8)
Yb(02)-N(3)	2.228(7)	Yb(02)-C(15)	2.58(1)
Yb(02)-C(12)	2.58(1)	Yb(02)-C(14)	2.59(1)
Yb(02)-C(13)	2.59(1)	Yb(02)-C(11)	2.59(1)
Yb(02)-C(5)	2.60(1)	Yb(02)-C(1)	2.607(8)
Yb(02)-C(4)	2.61(1)	Yb(02)-C(3)	2.62(1)
Yb(02)-C(2)	2.619(8)	Pd(03)-C(51)	2.05(1)
Pd(03)-C(52)	2.06(1)	Pd(03)-N(1)	2.118(7)
Pd(03)-N(2)	2.121(8)	N(1)-C(48)	1.34(1)
N(1)-C(47)	1.34(1)	N(2)-C(49)	1.33(1)
N(2)-C(41)	1.37(1)	N(3)-C(50)	1.35(1)
N(3)-N(4)	1.44(1)	N(4)-C(44)	1.35(1)
C(1)-C(2)	1.42(1)	C(1)-C(5)	1.43(1)
C(1)-C(6)	1.48(1)	C(2)-C(3)	1.43(1)
C(2)-C(7)	1.49(1)	C(3)-C(4)	1.41(2)
C(3)-C(8)	1.51(1)	C(4)-C(5)	1.42(1)
C(4)-C(9)	1.52(1)	C(5)-C(10)	1.49(1)
C(6)-H(6A)	0.9800	C(6)-H(6B)	0.9800
C(6)-H(6C)	0.9800	C(7)-H(7A)	0.9800
C(7)-H(7B)	0.9800	C(7)-H(7C)	0.9800
C(8)-H(8A)	0.9800	C(8)-H(8B)	0.9800
C(8)-H(8C)	0.9800	C(9)-H(9A)	0.9800
C(9)-H(9B)	0.9800	C(9)-H(9C)	0.9800
C(10)-H(10A)	0.9800	C(10)-H(10B)	0.9800
C(10)-H(10C)	0.9800	C(11)-C(15)	1.42(2)
C(11)-C(12)	1.43(2)	C(11)-C(16)	1.50(2)
C(12)-C(13)	1.39(2)	C(12)-C(17)	1.52(2)
C(13)-C(14)	1.39(2)	C(13)-C(18)	1.52(2)
C(14)-C(15)	1.37(2)	C(14)-C(19)	1.55(2)
C(15)-C(20)	1.52(2)	C(16)-H(16A)	0.9800
C(16)-H(16B)	0.9800	C(16)-H(16C)	0.9800
C(17)-H(17A)	0.9800	C(17)-H(17B)	0.9800
C(17)-H(17C)	0.9800	C(18)-H(18A)	0.9800
C(18)-H(18B)	0.9800	C(18)-H(18C)	0.9800
C(19)-H(19A)	0.9800	C(19)-H(19B)	0.9800
C(19)-H(19C)	0.9800	C(20)-H(20A)	0.9800
C(20)-H(20B)	0.9800	C(20)-H(20C)	0.9800
C(21)-C(25)	1.40(2)	C(21)-C(22)	1.41(1)
C(21)-C(26)	1.49(2)	C(22)-C(23)	1.43(1)
C(22)-C(27)	1.51(1)	C(23)-C(24)	1.41(1)
C(23)-C(28)	1.48(2)	C(24)-C(25)	1.40(2)
C(24)-C(29)	1.50(1)	C(25)-C(30)	1.52(1)
C(26)-H(26A)	0.9800	C(26)-H(26B)	0.9800
C(26)-H(26C)	0.9800	C(27)-H(27A)	0.9800
C(27)-H(27B)	0.9800	C(27)-H(27C)	0.9800
C(28)-H(28A)	0.9800	C(28)-H(28B)	0.9800
C(28)-H(28C)	0.9800	C(29)-H(29A)	0.9800
C(29)-H(29B)	0.9800	C(29)-H(29C)	0.9800
C(30)-H(30A)	0.9800	C(30)-H(30B)	0.9800
C(30)-H(30C)	0.9800	C(31)-C(35)	1.41(1)
C(31)-C(32)	1.43(1)	C(31)-C(36)	1.51(1)
C(32)-C(33)	1.42(1)	C(32)-C(37)	1.48(1)
C(33)-C(34)	1.42(2)	C(33)-C(38)	1.51(1)
C(34)-C(35)	1.41(1)	C(34)-C(39)	1.51(1)
C(35)-C(40)	1.51(1)	C(36)-H(36A)	0.9800
C(36)-H(36B)	0.9800	C(36)-H(36C)	0.9800
C(37)-H(37A)	0.9800	C(37)-H(37B)	0.9800
C(37)-H(37C)	0.9800	C(38)-H(38A)	0.9800
C(38)-H(38B)	0.9800	C(38)-H(38C)	0.9800
C(39)-H(39A)	0.9800	C(39)-H(39B)	0.9800
C(39)-H(39C)	0.9800	C(40)-H(40A)	0.9800
C(40)-H(40B)	0.9800	C(40)-H(40C)	0.9800
C(41)-C(42)	1.36(1)	C(41)-H(41)	0.9500
C(42)-C(43)	1.39(1)	C(42)-H(42)	0.9500
C(43)-C(50)	1.41(1)	C(43)-H(43)	0.9500
C(44)-C(48)	1.42(1)	C(44)-C(45)	1.44(1)
C(45)-C(46)	1.37(1)	C(45)-H(45)	0.9500
C(46)-C(47)	1.38(1)	C(46)-H(46)	0.9500
C(47)-H(47)	0.9500	C(48)-C(49)	1.45(1)

Table S11. Bond distances (Å) and Angle (deg) for **5** (continued).

C(49)-C(50)	1.42(1)	C(51)-H(51A)	1.1(1)
C(51)-H(51B)	1.2(1)	C(51)-H(51C)	0.8(1)
C(52)-H(52A)	1.0564	C(52)-H(52B)	1.0520
C(52)-H(52C)	0.8920		

C(52)-Yb(01)-C(23)	133.7(3)	C(52)-Yb(01)-C(51)	73.8(3)
C(23)-Yb(01)-C(51)	98.7(3)	C(52)-Yb(01)-C(22)	130.8(3)
C(23)-Yb(01)-C(22)	32.2(3)	C(51)-Yb(01)-C(22)	130.8(3)
C(52)-Yb(01)-C(21)	99.6(3)	C(23)-Yb(01)-C(21)	52.8(3)
C(51)-Yb(01)-C(21)	134.0(3)	C(22)-Yb(01)-C(21)	31.3(3)
C(52)-Yb(01)-C(24)	102.7(3)	C(23)-Yb(01)-C(24)	31.4(3)
C(51)-Yb(01)-C(24)	84.9(3)	C(22)-Yb(01)-C(24)	51.5(3)
C(21)-Yb(01)-C(24)	51.4(3)	C(52)-Yb(01)-C(35)	114.4(3)
C(23)-Yb(01)-C(35)	109.4(3)	C(51)-Yb(01)-C(35)	82.3(3)
C(22)-Yb(01)-C(35)	111.1(3)	C(21)-Yb(01)-C(35)	137.2(3)
C(24)-Yb(01)-C(35)	135.1(3)	C(52)-Yb(01)-C(25)	84.8(3)
C(23)-Yb(01)-C(25)	51.9(3)	C(51)-Yb(01)-C(25)	103.7(3)
C(22)-Yb(01)-C(25)	51.2(3)	C(21)-Yb(01)-C(25)	30.9(3)
C(24)-Yb(01)-C(25)	30.7(3)	C(35)-Yb(01)-C(25)	160.7(3)
C(52)-Yb(01)-C(33)	83.1(3)	C(23)-Yb(01)-C(33)	138.7(3)
C(51)-Yb(01)-C(33)	111.4(4)	C(22)-Yb(01)-C(33)	113.2(3)
C(21)-Yb(01)-C(33)	112.9(4)	C(24)-Yb(01)-C(33)	163.7(3)
C(35)-Yb(01)-C(33)	51.0(3)	C(25)-Yb(01)-C(33)	137.7(4)
C(52)-Yb(01)-C(31)	133.3(3)	C(23)-Yb(01)-C(31)	92.5(3)
C(51)-Yb(01)-C(31)	110.7(3)	C(22)-Yb(01)-C(31)	82.9(3)
C(21)-Yb(01)-C(31)	106.3(3)	C(24)-Yb(01)-C(31)	123.9(3)
C(35)-Yb(01)-C(31)	30.9(3)	C(25)-Yb(01)-C(31)	134.0(3)
C(33)-Yb(01)-C(31)	51.2(3)	C(52)-Yb(01)-C(32)	110.7(3)
C(23)-Yb(01)-C(32)	107.7(3)	C(51)-Yb(01)-C(32)	131.9(3)
C(22)-Yb(01)-C(32)	84.1(3)	C(21)-Yb(01)-C(32)	93.6(3)
C(24)-Yb(01)-C(32)	135.4(3)	C(35)-Yb(01)-C(32)	51.4(3)
C(25)-Yb(01)-C(32)	124.3(3)	C(33)-Yb(01)-C(32)	31.0(3)
C(31)-Yb(01)-C(32)	31.3(3)	C(52)-Yb(01)-C(34)	85.3(3)
C(23)-Yb(01)-C(34)	140.1(3)	C(51)-Yb(01)-C(34)	82.5(3)
C(22)-Yb(01)-C(34)	132.8(3)	C(21)-Yb(01)-C(34)	143.3(3)
C(24)-Yb(01)-C(34)	162.6(3)	C(35)-Yb(01)-C(34)	30.8(3)
C(25)-Yb(01)-C(34)	166.3(3)	C(33)-Yb(01)-C(34)	31.1(3)
C(31)-Yb(01)-C(34)	51.3(3)	C(32)-Yb(01)-C(34)	51.6(3)
C(52)-Yb(01)-H(51A)	79(3)	C(23)-Yb(01)-H(51A)	113(3)
C(51)-Yb(01)-H(51A)	25(3)	C(22)-Yb(01)-H(51A)	143(3)
C(21)-Yb(01)-H(51A)	159(3)	C(24)-Yb(01)-H(51A)	108(3)
C(35)-Yb(01)-H(51A)	59(3)	C(25)-Yb(01)-H(51A)	129(3)
C(33)-Yb(01)-H(51A)	88(3)	C(31)-Yb(01)-H(51A)	89(3)
C(32)-Yb(01)-H(51A)	107(3)	C(34)-Yb(01)-H(51A)	58(3)
C(52)-Yb(01)-H(52B)	24.2	C(23)-Yb(01)-H(52B)	157.9
C(51)-Yb(01)-H(52B)	76.0	C(22)-Yb(01)-H(52B)	145.7
C(21)-Yb(01)-H(52B)	116.0	C(24)-Yb(01)-H(52B)	126.7
C(35)-Yb(01)-H(52B)	91.3	C(25)-Yb(01)-H(52B)	107.9
C(33)-Yb(01)-H(52B)	61.0	C(31)-Yb(01)-H(52B)	109.4
C(32)-Yb(01)-H(52B)	90.9	C(34)-Yb(01)-H(52B)	61.3
H(51A)-Yb(01)-H(52B)	70.8	C(52)-Yb(01)-H(52C)	20.4
C(23)-Yb(01)-H(52C)	119.3	C(51)-Yb(01)-H(52C)	89.1
C(22)-Yb(01)-H(52C)	110.6	C(21)-Yb(01)-H(52C)	79.3
C(24)-Yb(01)-H(52C)	91.2	C(35)-Yb(01)-H(52C)	131.3
C(25)-Yb(01)-H(52C)	67.7	C(33)-Yb(01)-H(52C)	89.6
C(31)-Yb(01)-H(52C)	140.0	C(32)-Yb(01)-H(52C)	110.4
C(34)-Yb(01)-H(52C)	100.6	H(51A)-Yb(01)-H(52C)	98.7
H(52B)-Yb(01)-H(52C)	40.3	N(4)-Yb(02)-N(3)	38.0(3)
N(4)-Yb(02)-C(15)	127.4(4)	N(3)-Yb(02)-C(15)	135.8(3)
N(4)-Yb(02)-C(12)	81.6(3)	N(3)-Yb(02)-C(12)	84.4(4)
C(15)-Yb(02)-C(12)	52.5(4)	N(4)-Yb(02)-C(14)	132.2(3)
N(3)-Yb(02)-C(14)	116.4(4)	C(15)-Yb(02)-C(14)	30.7(4)
C(12)-Yb(02)-C(14)	51.9(4)	N(4)-Yb(02)-C(13)	102.6(4)
N(3)-Yb(02)-C(13)	87.2(3)	C(15)-Yb(02)-C(13)	51.4(4)
C(12)-Yb(02)-C(13)	31.2(4)	C(14)-Yb(02)-C(13)	31.1(4)
N(4)-Yb(02)-C(11)	95.5(4)	N(3)-Yb(02)-C(11)	112.8(4)
C(15)-Yb(02)-C(11)	31.9(5)	C(12)-Yb(02)-C(11)	32.0(4)
C(14)-Yb(02)-C(11)	51.8(4)	C(13)-Yb(02)-C(11)	51.9(3)
N(4)-Yb(02)-C(5)	108.6(3)	N(3)-Yb(02)-C(5)	92.5(3)
C(15)-Yb(02)-C(5)	123.5(4)	C(12)-Yb(02)-C(5)	158.7(4)
C(14)-Yb(02)-C(5)	112.6(3)	C(13)-Yb(02)-C(5)	127.8(3)
C(11)-Yb(02)-C(5)	154.1(4)	N(4)-Yb(02)-C(1)	81.7(3)
N(3)-Yb(02)-C(1)	81.1(3)	C(15)-Yb(02)-C(1)	143.0(3)
C(12)-Yb(02)-C(1)	163.1(3)	C(14)-Yb(02)-C(1)	143.8(3)
C(13)-Yb(02)-C(1)	154.8(3)	C(11)-Yb(02)-C(1)	153.2(3)
C(5)-Yb(02)-C(1)	31.8(3)	N(4)-Yb(02)-C(4)	133.7(3)

Table S11. Bond distances (Å) and Angle (deg) for **5** (continued).

N(3)-Yb(02)-C(4)	124.2(3)	C(15)-Yb(02)-C(4)	94.1(4)
C(12)-Yb(02)-C(4)	144.6(4)	C(14)-Yb(02)-C(4)	93.7(4)
C(13)-Yb(02)-C(4)	121.0(4)	C(11)-Yb(02)-C(4)	122.6(5)
C(5)-Yb(02)-C(4)	31.8(3)	C(1)-Yb(02)-C(4)	52.3(3)
N(4)-Yb(02)-C(3)	117.7(3)	N(3)-Yb(02)-C(3)	133.1(3)
C(15)-Yb(02)-C(3)	91.0(3)	C(12)-Yb(02)-C(3)	139.9(4)
C(14)-Yb(02)-C(3)	106.4(4)	C(13)-Yb(02)-C(3)	137.5(4)
C(11)-Yb(02)-C(3)	108.1(4)	C(5)-Yb(02)-C(3)	52.4(3)
C(1)-Yb(02)-C(3)	52.4(3)	C(4)-Yb(02)-C(3)	31.2(3)
N(4)-Yb(02)-C(2)	86.9(3)	N(3)-Yb(02)-C(2)	104.0(3)
C(15)-Yb(02)-C(2)	118.0(3)	C(12)-Yb(02)-C(2)	148.7(4)
C(14)-Yb(02)-C(2)	138.1(4)	C(13)-Yb(02)-C(2)	168.8(3)
C(11)-Yb(02)-C(2)	122.0(3)	C(5)-Yb(02)-C(2)	52.3(3)
C(1)-Yb(02)-C(2)	31.5(3)	C(4)-Yb(02)-C(2)	52.0(3)
C(3)-Yb(02)-C(2)	31.7(3)	C(51)-Pd(03)-C(52)	97.2(4)
C(51)-Pd(03)-N(1)	171.1(4)	C(52)-Pd(03)-N(1)	91.6(3)
C(51)-Pd(03)-N(2)	90.9(4)	C(52)-Pd(03)-N(2)	171.6(3)
N(1)-Pd(03)-N(2)	80.2(3)	C(51)-Pd(03)-Yb(01)	55.9(3)
C(52)-Pd(03)-Yb(01)	55.6(2)	N(1)-Pd(03)-Yb(01)	130.9(2)
N(2)-Pd(03)-Yb(01)	132.0(2)	C(48)-N(1)-C(47)	119.0(8)
C(48)-N(1)-Pd(03)	110.5(6)	C(47)-N(1)-Pd(03)	130.4(6)
C(49)-N(2)-C(41)	119.4(8)	C(49)-N(2)-Pd(03)	110.8(6)
C(41)-N(2)-Pd(03)	129.4(6)	C(50)-N(3)-N(4)	120.3(7)
C(50)-N(3)-Yb(02)	166.7(7)	N(4)-N(3)-Yb(02)	69.9(4)
C(44)-N(4)-N(3)	119.2(8)	C(44)-N(4)-Yb(02)	163.1(7)
N(3)-N(4)-Yb(02)	72.1(4)	C(2)-C(1)-C(5)	107.9(8)
C(2)-C(1)-C(6)	125(1)	C(5)-C(1)-C(6)	127(1)
C(2)-C(1)-Yb(02)	74.7(5)	C(5)-C(1)-Yb(02)	73.8(5)
C(6)-C(1)-Yb(02)	119.2(6)	C(1)-C(2)-C(3)	108.1(8)
C(1)-C(2)-C(7)	126(1)	C(3)-C(2)-C(7)	126(1)
C(1)-C(2)-Yb(02)	73.8(5)	C(3)-C(2)-Yb(02)	74.1(5)
C(7)-C(2)-Yb(02)	119.0(6)	C(4)-C(3)-C(2)	107.7(8)
C(4)-C(3)-C(8)	129(1)	C(2)-C(3)-C(8)	123(1)
C(4)-C(3)-Yb(02)	74.0(5)	C(2)-C(3)-Yb(02)	74.2(5)
C(8)-C(3)-Yb(02)	124.9(7)	C(3)-C(4)-C(5)	109(1)
C(3)-C(4)-C(9)	127(1)	C(5)-C(4)-C(9)	123(1)
C(3)-C(4)-Yb(02)	74.8(5)	C(5)-C(4)-Yb(02)	73.7(5)
C(9)-C(4)-Yb(02)	125.9(8)	C(4)-C(5)-C(1)	107.5(8)
C(4)-C(5)-C(10)	128(1)	C(1)-C(5)-C(10)	125(1)
C(4)-C(5)-Yb(02)	74.5(5)	C(1)-C(5)-Yb(02)	74.4(5)
C(10)-C(5)-Yb(02)	120.2(7)	C(1)-C(6)-H(6A)	109.5
C(1)-C(6)-H(6B)	109.5	H(6A)-C(6)-H(6B)	109.5
C(1)-C(6)-H(6C)	109.5	H(6A)-C(6)-H(6C)	109.5
H(6B)-C(6)-H(6C)	109.5	C(2)-C(7)-H(7A)	109.5
C(2)-C(7)-H(7B)	109.5	H(7A)-C(7)-H(7B)	109.5
C(2)-C(7)-H(7C)	109.5	H(7A)-C(7)-H(7C)	109.5
H(7B)-C(7)-H(7C)	109.5	C(3)-C(8)-H(8A)	109.5
C(3)-C(8)-H(8B)	109.5	H(8A)-C(8)-H(8B)	109.5
C(3)-C(8)-H(8C)	109.5	H(8A)-C(8)-H(8C)	109.5
H(8B)-C(8)-H(8C)	109.5	C(4)-C(9)-H(9A)	109.5
C(4)-C(9)-H(9B)	109.5	H(9A)-C(9)-H(9B)	109.5
C(4)-C(9)-H(9C)	109.5	H(9A)-C(9)-H(9C)	109.5
H(9B)-C(9)-H(9C)	109.5	C(5)-C(10)-H(10A)	109.5
C(5)-C(10)-H(10B)	109.5	H(10A)-C(10)-H(10B)	109.5
C(5)-C(10)-H(10C)	109.5	H(10A)-C(10)-H(10C)	109.5
H(10B)-C(10)-H(10C)	109.5	C(15)-C(11)-C(12)	107(1)
C(15)-C(11)-C(16)	127(2)	C(12)-C(11)-C(16)	127(2)
C(15)-C(11)-Yb(02)	73.5(6)	C(12)-C(11)-Yb(02)	73.5(6)
C(16)-C(11)-Yb(02)	119.2(8)	C(13)-C(12)-C(11)	107(1)
C(13)-C(12)-C(17)	126(1)	C(11)-C(12)-C(17)	127(1)
C(13)-C(12)-Yb(02)	74.8(6)	C(11)-C(12)-Yb(02)	74.5(6)
C(17)-C(12)-Yb(02)	121.1(8)	C(14)-C(13)-C(12)	109(1)
C(14)-C(13)-C(18)	128(1)	C(12)-C(13)-C(18)	123(1)
C(14)-C(13)-Yb(02)	74.3(6)	C(12)-C(13)-Yb(02)	74.0(6)
C(18)-C(13)-Yb(02)	118.0(7)	C(15)-C(14)-C(13)	109(1)
C(15)-C(14)-C(19)	127(1)	C(13)-C(14)-C(19)	124(1)
C(15)-C(14)-Yb(02)	74.4(6)	C(13)-C(14)-Yb(02)	74.6(6)
C(19)-C(14)-Yb(02)	122.7(8)	C(14)-C(15)-C(11)	108(1)
C(14)-C(15)-C(20)	125(2)	C(11)-C(15)-C(20)	126(2)
C(14)-C(15)-Yb(02)	74.9(6)	C(11)-C(15)-Yb(02)	74.6(6)
C(20)-C(15)-Yb(02)	125.9(8)	C(11)-C(16)-H(16A)	109.5
C(11)-C(16)-H(16B)	109.5	H(16A)-C(16)-H(16B)	109.5
C(11)-C(16)-H(16C)	109.5	H(16A)-C(16)-H(16C)	109.5
H(16B)-C(16)-H(16C)	109.5	C(12)-C(17)-H(17A)	109.5
C(12)-C(17)-H(17B)	109.5	H(17A)-C(17)-H(17B)	109.5
C(12)-C(17)-H(17C)	109.5	H(17A)-C(17)-H(17C)	109.5
H(17B)-C(17)-H(17C)	109.5	C(13)-C(18)-H(18A)	109.5

Table S11. Bond distances (Å) and Angle (deg) for **5** (continued).

C(13)-C(18)-H(18B)	109.5	H(18A)-C(18)-H(18B)	109.5
C(13)-C(18)-H(18C)	109.5	H(18A)-C(18)-H(18C)	109.5
H(18B)-C(18)-H(18C)	109.5	C(14)-C(19)-H(19A)	109.5
C(14)-C(19)-H(19B)	109.5	H(19A)-C(19)-H(19B)	109.5
C(14)-C(19)-H(19C)	109.5	H(19A)-C(19)-H(19C)	109.5
H(19B)-C(19)-H(19C)	109.5	C(15)-C(20)-H(20A)	109.5
C(15)-C(20)-H(20B)	109.5	H(20A)-C(20)-H(20B)	109.5
C(15)-C(20)-H(20C)	109.5	H(20A)-C(20)-H(20C)	109.5
H(20B)-C(20)-H(20C)	109.5	C(25)-C(21)-C(22)	107(1)
C(25)-C(21)-C(26)	125(1)	C(22)-C(21)-C(26)	127(1)
C(25)-C(21)-Yb(01)	75.4(6)	C(22)-C(21)-Yb(01)	73.7(6)
C(26)-C(21)-Yb(01)	123.6(8)	C(21)-C(22)-C(23)	108.5(8)
C(21)-C(22)-C(27)	123(1)	C(23)-C(22)-C(27)	126(1)
C(21)-C(22)-Yb(01)	75.0(6)	C(23)-C(22)-Yb(01)	72.7(5)
C(27)-C(22)-Yb(01)	132.2(7)	C(24)-C(23)-C(22)	106(1)
C(24)-C(23)-C(28)	125(1)	C(22)-C(23)-C(28)	128(1)
C(24)-C(23)-Yb(01)	76.7(5)	C(22)-C(23)-Yb(01)	75.2(5)
C(28)-C(23)-Yb(01)	121.2(7)	C(25)-C(24)-C(23)	109(1)
C(25)-C(24)-C(29)	125(1)	C(23)-C(24)-C(29)	125(1)
C(25)-C(24)-Yb(01)	75.1(6)	C(23)-C(24)-Yb(01)	71.9(5)
C(29)-C(24)-Yb(01)	126.9(7)	C(24)-C(25)-C(21)	109(1)
C(24)-C(25)-C(30)	127(1)	C(21)-C(25)-C(30)	123(1)
C(24)-C(25)-Yb(01)	74.2(6)	C(21)-C(25)-Yb(01)	73.7(6)
C(30)-C(25)-Yb(01)	126.9(7)	C(21)-C(26)-H(26A)	109.5
C(21)-C(26)-H(26B)	109.5	H(26A)-C(26)-H(26B)	109.5
C(21)-C(26)-H(26C)	109.5	H(26A)-C(26)-H(26C)	109.5
H(26B)-C(26)-H(26C)	109.5	C(22)-C(27)-H(27A)	109.5
C(22)-C(27)-H(27B)	109.5	H(27A)-C(27)-H(27B)	109.5
C(22)-C(27)-H(27C)	109.5	H(27A)-C(27)-H(27C)	109.5
H(27B)-C(27)-H(27C)	109.5	C(23)-C(28)-H(28A)	109.5
C(23)-C(28)-H(28B)	109.5	H(28A)-C(28)-H(28B)	109.5
C(23)-C(28)-H(28C)	109.5	H(28A)-C(28)-H(28C)	109.5
H(28B)-C(28)-H(28C)	109.5	C(24)-C(29)-H(29A)	109.5
C(24)-C(29)-H(29B)	109.5	H(29A)-C(29)-H(29B)	109.5
C(24)-C(29)-H(29C)	109.5	H(29A)-C(29)-H(29C)	109.5
H(29B)-C(29)-H(29C)	109.5	C(25)-C(30)-H(30A)	109.5
C(25)-C(30)-H(30B)	109.5	H(30A)-C(30)-H(30B)	109.5
C(25)-C(30)-H(30C)	109.5	H(30A)-C(30)-H(30C)	109.5
H(30B)-C(30)-H(30C)	109.5	C(35)-C(31)-C(32)	107.8(8)
C(35)-C(31)-C(36)	124(1)	C(32)-C(31)-C(36)	128(1)
C(35)-C(31)-Yb(01)	74.2(5)	C(32)-C(31)-Yb(01)	74.4(5)
C(36)-C(31)-Yb(01)	126.3(7)	C(33)-C(32)-C(31)	107.0(8)
C(33)-C(32)-C(37)	124(1)	C(31)-C(32)-C(37)	128(1)
C(33)-C(32)-Yb(01)	74.2(5)	C(31)-C(32)-Yb(01)	74.3(5)
C(37)-C(32)-Yb(01)	127.2(7)	C(32)-C(33)-C(34)	109(1)
C(32)-C(33)-C(38)	123(1)	C(34)-C(33)-C(38)	127(1)
C(32)-C(33)-Yb(01)	74.8(5)	C(34)-C(33)-Yb(01)	74.8(5)
C(38)-C(33)-Yb(01)	122.6(7)	C(35)-C(34)-C(33)	107(1)
C(35)-C(34)-C(39)	127(1)	C(33)-C(34)-C(39)	126(1)
C(35)-C(34)-Yb(01)	74.1(5)	C(33)-C(34)-Yb(01)	74.1(5)
C(39)-C(34)-Yb(01)	121.6(6)	C(34)-C(35)-C(31)	109(1)
C(34)-C(35)-C(40)	127(1)	C(31)-C(35)-C(40)	124(1)
C(34)-C(35)-Yb(01)	75.1(5)	C(31)-C(35)-Yb(01)	74.9(5)
C(40)-C(35)-Yb(01)	121.2(6)	C(31)-C(36)-H(36A)	109.5
C(31)-C(36)-H(36B)	109.5	H(36A)-C(36)-H(36B)	109.5
C(31)-C(36)-H(36C)	109.5	H(36A)-C(36)-H(36C)	109.5
H(36B)-C(36)-H(36C)	109.5	C(32)-C(37)-H(37A)	109.5
C(32)-C(37)-H(37B)	109.5	H(37A)-C(37)-H(37B)	109.5
C(32)-C(37)-H(37C)	109.5	H(37A)-C(37)-H(37C)	109.5
H(37B)-C(37)-H(37C)	109.5	C(33)-C(38)-H(38A)	109.5
C(33)-C(38)-H(38B)	109.5	H(38A)-C(38)-H(38B)	109.5
C(33)-C(38)-H(38C)	109.5	H(38A)-C(38)-H(38C)	109.5
H(38B)-C(38)-H(38C)	109.5	C(34)-C(39)-H(39A)	109.5
C(34)-C(39)-H(39B)	109.5	H(39A)-C(39)-H(39B)	109.5
C(34)-C(39)-H(39C)	109.5	H(39A)-C(39)-H(39C)	109.5
H(39B)-C(39)-H(39C)	109.5	C(35)-C(40)-H(40A)	109.5
C(35)-C(40)-H(40B)	109.5	H(40A)-C(40)-H(40B)	109.5
C(35)-C(40)-H(40C)	109.5	H(40A)-C(40)-H(40C)	109.5
H(40B)-C(40)-H(40C)	109.5	C(42)-C(41)-N(2)	120(1)
C(42)-C(41)-H(41)	120.3	N(2)-C(41)-H(41)	120.3
C(41)-C(42)-C(43)	123(1)	C(41)-C(42)-H(42)	118.5
C(43)-C(42)-H(42)	118.5	C(42)-C(43)-C(50)	118(1)
C(42)-C(43)-H(43)	121.1	C(50)-C(43)-H(43)	121.1
N(4)-C(44)-C(48)	123(1)	N(4)-C(44)-C(45)	121.1(8)
C(48)-C(44)-C(45)	116.3(8)	C(46)-C(45)-C(44)	118(1)
C(46)-C(45)-H(45)	120.9	C(44)-C(45)-H(45)	120.9

Table S11. Bond distances (Å) and Angle (deg) for **5** (continued).

C(45)-C(46)-C(47)	122(1)	C(45)-C(46)-H(46)	119.2
C(47)-C(46)-H(46)	119.2	N(1)-C(47)-C(46)	121.6(8)
N(1)-C(47)-H(47)	119.2	C(46)-C(47)-H(47)	119.2
N(1)-C(48)-C(44)	123.3(8)	N(1)-C(48)-C(49)	118.9(8)
C(44)-C(48)-C(49)	117.9(8)	N(2)-C(49)-C(50)	123.9(8)
N(2)-C(49)-C(48)	118.4(8)	C(50)-C(49)-C(48)	117.6(8)
N(3)-C(50)-C(43)	121.3(8)	N(3)-C(50)-C(49)	122.3(8)
C(43)-C(50)-C(49)	116.4(8)	Pd(03)-C(51)-Yb(01)	82.8(3)
Pd(03)-C(51)-H(51A)	122(6)	Yb(01)-C(51)-H(51A)	64(6)
Pd(03)-C(51)-H(51B)	114(5)	Yb(01)-C(51)-H(51B)	89(5)
H(51A)-C(51)-H(51B)	112(8)	Pd(03)-C(51)-H(51C)	120(9)
Yb(01)-C(51)-H(51C)	149(9)	H(51A)-C(51)-H(51C)	86(10)
H(51B)-C(51)-H(51C)	98(10)	Pd(03)-C(52)-Yb(01)	83.0(3)
Pd(03)-C(52)-H(52A)	109.5	Yb(01)-C(52)-H(52A)	167.5
Pd(03)-C(52)-H(52B)	117.6	Yb(01)-C(52)-H(52B)	68.5
H(52A)-C(52)-H(52B)	104.6	Pd(03)-C(52)-H(52C)	102.8
Yb(01)-C(52)-H(52C)	71.3	H(52A)-C(52)-H(52C)	104.5
H(52B)-C(52)-H(52C)	117.0		

Table S12. Bond distances (Å) and Angle (deg) for **6**.

Pt(1)-C(51)	2.07(1)	Pt(1)-C(52)	2.08(1)
Pt(1)-N(1)	2.09(1)	Pt(1)-N(2)	2.12(1)
Pt(1)-Yb(1)	3.0493(7	Yb(1)-C(51)	2.56(1)
Yb(1)-C(5)	2.59(1)	Yb(1)-C(4)	2.60(1)
Yb(1)-C(52)	2.60(1)	Yb(1)-C(3)	2.62(1)
Yb(1)-C(1)	2.63(1)	Yb(1)-C(13)	2.65(1)
Yb(1)-C(14)	2.65(1)	Yb(1)-C(15)	2.66(1)
Yb(1)-C(11)	2.66(1)	Yb(1)-C(12)	2.66(1)
Yb(1)-C(2)	2.69(1)	Yb(2)-N(4)	2.21(1)
Yb(2)-N(3)	2.22(1)	Yb(2)-C(44)	2.58(1)
Yb(2)-C(45)	2.58(1)	Yb(2)-C(41)	2.59(1)
Yb(2)-C(42)	2.59(1)	Yb(2)-C(34)	2.60(1)
Yb(2)-C(32)	2.61(1)	Yb(2)-C(35)	2.61(1)
Yb(2)-C(43)	2.61(1)	Yb(2)-C(33)	2.61(1)
Yb(2)-C(31)	2.61(1)	N(1)-C(21)	1.33(1)
N(1)-C(30)	1.37(2)	N(2)-C(22)	1.34(1)
N(2)-C(23)	1.37(1)	N(3)-C(26)	1.36(1)
N(3)-N(4)	1.45(1)	N(4)-C(27)	1.34(1)
C(1)-C(2)	1.41(2)	C(1)-C(5)	1.42(2)
C(1)-C(10)	1.50(2)	C(2)-C(3)	1.42(2)
C(2)-C(9)	1.49(2)	C(3)-C(4)	1.40(2)
C(3)-C(8)	1.52(2)	C(4)-C(5)	1.43(2)
C(4)-C(7)	1.51(2)	C(5)-C(6)	1.50(2)
C(6)-H(1)	0.9800	C(6)-H(2)	0.9800
C(6)-H(3)	0.9800	C(7)-H(4)	0.9800
C(7)-H(5)	0.9800	C(7)-H(6)	0.9800
C(8)-H(7)	0.9800	C(8)-H(8)	0.9800
C(8)-H(9)	0.9800	C(9)-H(10)	0.9800
C(9)-H(11)	0.9800	C(9)-H(12)	0.9800
C(10)-H(13)	0.9800	C(10)-H(14)	0.9800
C(10)-H(15)	0.9800	C(11)-C(12)	1.39(2)
C(11)-C(15)	1.42(2)	C(11)-C(20)	1.52(2)
C(12)-C(13)	1.41(2)	C(12)-C(19)	1.51(2)
C(13)-C(14)	1.39(2)	C(13)-C(18)	1.54(2)
C(14)-C(15)	1.42(2)	C(14)-C(17)	1.51(2)
C(15)-C(16)	1.52(2)	C(16)-H(16)	0.9800
C(16)-H(17)	0.9800	C(16)-H(18)	0.9800
C(17)-H(19)	0.9800	C(17)-H(20)	0.9800
C(17)-H(21)	0.9800	C(18)-H(22)	0.9800
C(18)-H(23)	0.9800	C(18)-H(24)	0.9800
C(19)-H(25)	0.9800	C(19)-H(26)	0.9800
C(19)-H(27)	0.9800	C(20)-H(28)	0.9800
C(20)-H(29)	0.9800	C(20)-H(30)	0.9800
C(21)-C(27)	1.40(2)	C(21)-C(22)	1.47(2)
C(22)-C(26)	1.41(2)	C(23)-C(24)	1.39(2)
C(23)-H(66)	0.9500	C(24)-C(25)	1.38(2)
C(24)-H(65)	0.9500	C(25)-C(26)	1.44(2)
C(25)-H(64)	0.9500	C(27)-C(28)	1.41(2)
C(28)-C(29)	1.38(2)	C(28)-H(33)	0.9500
C(29)-C(30)	1.38(2)	C(29)-H(32)	0.9500
C(30)-H(31)	0.9500	C(31)-C(32)	1.42(2)
C(31)-C(35)	1.42(2)	C(31)-C(40)	1.47(2)
C(32)-C(33)	1.43(2)	C(32)-C(39)	1.50(2)
C(33)-C(34)	1.41(2)	C(33)-C(38)	1.48(2)
C(34)-C(35)	1.41(2)	C(34)-C(37)	1.53(2)
C(35)-C(36)	1.52(2)	C(36)-H(34)	0.9800
C(36)-H(35)	0.9800	C(36)-H(36)	0.9800
C(37)-H(37)	0.9800	C(37)-H(38)	0.9800
C(37)-H(39)	0.9800	C(38)-H(40)	0.9800
C(38)-H(41)	0.9800	C(38)-H(42)	0.9800
C(39)-H(43)	0.9800	C(39)-H(44)	0.9800
C(39)-H(45)	0.9800	C(40)-H(46)	0.9800
C(40)-H(47)	0.9800	C(40)-H(48)	0.9800
C(41)-C(42)	1.34(2)	C(41)-C(45)	1.44(2)
C(41)-C(50)	1.51(2)	C(42)-C(43)	1.38(2)
C(42)-C(49)	1.52(2)	C(43)-C(44)	1.41(2)
C(43)-C(48)	1.52(2)	C(44)-C(45)	1.40(2)
C(44)-C(47)	1.50(2)	C(45)-C(46)	1.50(2)
C(46)-H(49)	0.9800	C(46)-H(50)	0.9800
C(46)-H(51)	0.9800	C(47)-H(52)	0.9800
C(47)-H(53)	0.9800	C(47)-H(54)	0.9800
C(48)-H(55)	0.9800	C(48)-H(56)	0.9800
C(48)-H(57)	0.9800	C(49)-H(58)	0.9800
C(49)-H(59)	0.9800	C(49)-H(60)	0.9800
C(50)-H(61)	0.9800	C(50)-H(62)	0.9800
C(50)-H(63)	0.9800	C(51)-H(51A)	0.9800
C(51)-H(51B)	0.9800	C(51)-H(51C)	0.9800

Table S12. Bond distances (Å) and Angle (deg) for **6** (Continued).

C(52)-H(52A)	0.9800	C(52)-H(52B)	0.9800
C(52)-H(52C)	0.9800		

C(51)-Pt(1)-C(52)	97.1(5)	C(51)-Pt(1)-N(1)	92.1(4)
C(52)-Pt(1)-N(1)	170.6(5)	C(51)-Pt(1)-N(2)	171.8(4)
C(52)-Pt(1)-N(2)	90.9(5)	N(1)-Pt(1)-N(2)	79.9(4)
C(51)-Pt(1)-Yb(1)	56.2(3)	C(52)-Pt(1)-Yb(1)	57.3(4)
N(1)-Pt(1)-Yb(1)	130.8(3)	N(2)-Pt(1)-Yb(1)	131.0(3)
C(51)-Yb(1)-C(5)	135.3(4)	C(51)-Yb(1)-C(4)	131.4(4)
C(5)-Yb(1)-C(4)	32.0(4)	C(51)-Yb(1)-C(52)	73.9(4)
C(5)-Yb(1)-C(52)	100.8(4)	C(4)-Yb(1)-C(52)	132.8(5)
C(51)-Yb(1)-C(3)	100.4(4)	C(5)-Yb(1)-C(3)	52.5(4)
C(4)-Yb(1)-C(3)	31.0(4)	C(52)-Yb(1)-C(3)	136.2(4)
C(51)-Yb(1)-C(1)	104.1(4)	C(5)-Yb(1)-C(1)	31.7(4)
C(4)-Yb(1)-C(1)	51.8(4)	C(52)-Yb(1)-C(1)	86.9(4)
C(3)-Yb(1)-C(1)	51.7(4)	C(51)-Yb(1)-C(13)	131.7(4)
C(5)-Yb(1)-C(13)	92.5(4)	C(4)-Yb(1)-C(13)	82.9(4)
C(52)-Yb(1)-C(13)	109.6(4)	C(3)-Yb(1)-C(13)	105.9(4)
C(1)-Yb(1)-C(13)	124.1(4)	C(51)-Yb(1)-C(14)	113.8(4)
C(5)-Yb(1)-C(14)	109.0(4)	C(4)-Yb(1)-C(14)	110.5(4)
C(52)-Yb(1)-C(14)	81.2(4)	C(3)-Yb(1)-C(14)	136.3(4)
C(1)-Yb(1)-C(14)	134.9(4)	C(13)-Yb(1)-C(14)	30.4(4)
C(51)-Yb(1)-C(15)	84.3(4)	C(5)-Yb(1)-C(15)	139.8(4)
C(4)-Yb(1)-C(15)	132.1(4)	C(52)-Yb(1)-C(15)	81.2(4)
C(3)-Yb(1)-C(15)	142.3(4)	C(1)-Yb(1)-C(15)	163.0(4)
C(13)-Yb(1)-C(15)	50.7(4)	C(14)-Yb(1)-C(15)	31.1(4)
C(51)-Yb(1)-C(11)	82.7(4)	C(5)-Yb(1)-C(11)	137.3(4)
C(4)-Yb(1)-C(11)	112.0(4)	C(52)-Yb(1)-C(11)	110.2(4)
C(3)-Yb(1)-C(11)	112.0(5)	C(1)-Yb(1)-C(11)	162.9(4)
C(13)-Yb(1)-C(11)	50.1(4)	C(14)-Yb(1)-C(11)	50.7(4)
C(15)-Yb(1)-C(11)	30.9(4)	C(51)-Yb(1)-C(12)	109.6(4)
C(5)-Yb(1)-C(12)	107.1(4)	C(4)-Yb(1)-C(12)	83.6(4)
C(52)-Yb(1)-C(12)	130.1(4)	C(3)-Yb(1)-C(12)	93.2(5)
C(1)-Yb(1)-C(12)	135.2(4)	C(13)-Yb(1)-C(12)	30.8(4)
C(14)-Yb(1)-C(12)	51.0(4)	C(15)-Yb(1)-C(12)	51.0(4)
C(11)-Yb(1)-C(12)	30.2(4)	C(51)-Yb(1)-C(2)	86.2(4)
C(5)-Yb(1)-C(2)	51.7(4)	C(4)-Yb(1)-C(2)	51.0(4)
C(52)-Yb(1)-C(2)	105.8(4)	C(3)-Yb(1)-C(2)	31.0(4)
C(1)-Yb(1)-C(2)	30.7(4)	C(13)-Yb(1)-C(2)	133.7(4)
C(14)-Yb(1)-C(2)	160.0(4)	C(15)-Yb(1)-C(2)	166.1(5)
C(11)-Yb(1)-C(2)	137.5(4)	C(12)-Yb(1)-C(2)	124.1(4)
N(4)-Yb(2)-N(3)	38.1(4)	N(4)-Yb(2)-C(44)	95.2(5)
N(3)-Yb(2)-C(44)	112.8(5)	N(4)-Yb(2)-C(45)	81.5(4)
N(3)-Yb(2)-C(45)	84.8(4)	C(44)-Yb(2)-C(45)	31.6(5)
N(4)-Yb(2)-C(41)	103.5(5)	N(3)-Yb(2)-C(41)	88.0(5)
C(44)-Yb(2)-C(41)	52.2(5)	C(45)-Yb(2)-C(41)	32.3(5)
N(4)-Yb(2)-C(42)	132.0(5)	N(3)-Yb(2)-C(42)	116.4(5)
C(44)-Yb(2)-C(42)	51.8(5)	C(45)-Yb(2)-C(42)	51.8(5)
C(41)-Yb(2)-C(42)	30.1(5)	N(4)-Yb(2)-C(34)	133.7(4)
N(3)-Yb(2)-C(34)	123.8(4)	C(44)-Yb(2)-C(34)	122.9(6)
C(45)-Yb(2)-C(34)	144.6(4)	C(41)-Yb(2)-C(34)	120.0(5)
C(42)-Yb(2)-C(34)	93.9(5)	N(4)-Yb(2)-C(32)	81.9(4)
N(3)-Yb(2)-C(32)	81.3(4)	C(44)-Yb(2)-C(32)	152.9(4)
C(45)-Yb(2)-C(32)	163.4(4)	C(41)-Yb(2)-C(32)	154.7(4)
C(42)-Yb(2)-C(32)	143.9(4)	C(34)-Yb(2)-C(32)	52.0(4)
N(4)-Yb(2)-C(35)	117.4(4)	N(3)-Yb(2)-C(35)	132.7(4)
C(44)-Yb(2)-C(35)	108.3(5)	C(45)-Yb(2)-C(35)	139.7(5)
C(41)-Yb(2)-C(35)	136.9(5)	C(42)-Yb(2)-C(35)	106.8(5)
C(34)-Yb(2)-C(35)	31.5(5)	C(32)-Yb(2)-C(35)	51.8(4)
N(4)-Yb(2)-C(43)	126.7(5)	N(3)-Yb(2)-C(43)	135.4(4)
C(44)-Yb(2)-C(43)	31.5(5)	C(45)-Yb(2)-C(43)	51.8(5)
C(41)-Yb(2)-C(43)	50.7(5)	C(42)-Yb(2)-C(43)	30.7(5)
C(34)-Yb(2)-C(43)	94.8(5)	C(32)-Yb(2)-C(43)	143.1(4)
C(35)-Yb(2)-C(43)	91.7(5)	N(4)-Yb(2)-C(33)	108.7(4)
N(3)-Yb(2)-C(33)	92.4(4)	C(44)-Yb(2)-C(33)	154.2(6)
C(45)-Yb(2)-C(33)	159.0(5)	C(41)-Yb(2)-C(33)	127.0(5)
C(42)-Yb(2)-C(33)	112.7(5)	C(34)-Yb(2)-C(33)	31.5(4)
C(32)-Yb(2)-C(33)	31.9(4)	C(35)-Yb(2)-C(33)	52.3(4)
C(43)-Yb(2)-C(33)	124.0(5)	N(4)-Yb(2)-C(31)	86.7(4)
N(3)-Yb(2)-C(31)	103.8(4)	C(44)-Yb(2)-C(31)	121.7(5)
C(45)-Yb(2)-C(31)	147.9(5)	C(41)-Yb(2)-C(31)	168.2(5)
C(42)-Yb(2)-C(31)	138.4(5)	C(34)-Yb(2)-C(31)	52.5(5)
C(32)-Yb(2)-C(31)	31.5(4)	C(35)-Yb(2)-C(31)	31.6(4)
C(43)-Yb(2)-C(31)	118.2(5)	C(33)-Yb(2)-C(31)	52.8(4)
C(21)-N(1)-C(30)	117(1)	C(21)-N(1)-Pt(1)	113.6(8)
C(30)-N(1)-Pt(1)	129.5(8)	C(22)-N(2)-C(23)	120(1)

Table S12. Bond distances (Å) and Angle (deg) for **6** (Continued).

C(22)-N(2)-Pt(1)	111.1(8)	C(23)-N(2)-Pt(1)	128.3(8)
C(26)-N(3)-N(4)	119(1)	C(26)-N(3)-Yb(2)	167(1)
N(4)-N(3)-Yb(2)	70.4(6)	C(27)-N(4)-N(3)	121(1)
C(27)-N(4)-Yb(2)	163(1)	N(3)-N(4)-Yb(2)	71.5(6)
C(2)-C(1)-C(5)	109(1)	C(2)-C(1)-C(10)	126(1)
C(5)-C(1)-C(10)	124(1)	C(2)-C(1)-Yb(1)	77.0(7)
C(5)-C(1)-Yb(1)	72.6(7)	C(10)-C(1)-Yb(1)	127(1)
C(1)-C(2)-C(3)	108(1)	C(1)-C(2)-C(9)	127(1)
C(3)-C(2)-C(9)	125(2)	C(1)-C(2)-Yb(1)	72.2(7)
C(3)-C(2)-Yb(1)	71.8(7)	C(9)-C(2)-Yb(1)	125(1)
C(4)-C(3)-C(2)	108(1)	C(4)-C(3)-C(8)	126(1)
C(2)-C(3)-C(8)	125(1)	C(4)-C(3)-Yb(1)	73.8(8)
C(2)-C(3)-Yb(1)	77.1(8)	C(8)-C(3)-Yb(1)	124(1)
C(3)-C(4)-C(5)	109(1)	C(3)-C(4)-C(7)	124(2)
C(5)-C(4)-C(7)	124(1)	C(3)-C(4)-Yb(1)	75.2(8)
C(5)-C(4)-Yb(1)	73.4(7)	C(7)-C(4)-Yb(1)	133(1)
C(1)-C(5)-C(4)	106(1)	C(1)-C(5)-C(6)	126(1)
C(4)-C(5)-C(6)	128(1)	C(1)-C(5)-Yb(1)	75.7(7)
C(4)-C(5)-Yb(1)	74.6(8)	C(6)-C(5)-Yb(1)	121(1)
C(5)-C(6)-H(1)	109.5	C(5)-C(6)-H(2)	109.5
H(1)-C(6)-H(2)	109.5	C(5)-C(6)-H(3)	109.5
H(1)-C(6)-H(3)	109.5	H(2)-C(6)-H(3)	109.5
C(4)-C(7)-H(4)	109.5	C(4)-C(7)-H(5)	109.5
H(4)-C(7)-H(5)	109.5	C(4)-C(7)-H(6)	109.5
H(4)-C(7)-H(6)	109.5	H(5)-C(7)-H(6)	109.5
C(3)-C(8)-H(7)	109.5	C(3)-C(8)-H(8)	109.5
H(7)-C(8)-H(8)	109.5	C(3)-C(8)-H(9)	109.5
H(7)-C(8)-H(9)	109.5	H(8)-C(8)-H(9)	109.5
C(2)-C(9)-H(10)	109.5	C(2)-C(9)-H(11)	109.5
H(10)-C(9)-H(11)	109.5	C(2)-C(9)-H(12)	109.5
H(10)-C(9)-H(12)	109.5	H(11)-C(9)-H(12)	109.5
C(1)-C(10)-H(13)	109.5	C(1)-C(10)-H(14)	109.5
H(13)-C(10)-H(14)	109.5	C(1)-C(10)-H(15)	109.5
H(13)-C(10)-H(15)	109.5	H(14)-C(10)-H(15)	109.5
C(12)-C(11)-C(15)	110(1)	C(12)-C(11)-C(20)	125(2)
C(15)-C(11)-C(20)	126(2)	C(12)-C(11)-Yb(1)	74.9(7)
C(15)-C(11)-Yb(1)	74.5(7)	C(20)-C(11)-Yb(1)	121.3(8)
C(11)-C(12)-C(13)	107(1)	C(11)-C(12)-C(19)	124(1)
C(13)-C(12)-C(19)	128(1)	C(11)-C(12)-Yb(1)	74.9(7)
C(13)-C(12)-Yb(1)	74.0(7)	C(19)-C(12)-Yb(1)	126(1)
C(14)-C(13)-C(12)	110(1)	C(14)-C(13)-C(18)	123(1)
C(12)-C(13)-C(18)	127(1)	C(14)-C(13)-Yb(1)	74.8(7)
C(12)-C(13)-Yb(1)	75.3(7)	C(18)-C(13)-Yb(1)	126(1)
C(13)-C(14)-C(15)	108(1)	C(13)-C(14)-C(17)	126(1)
C(15)-C(14)-C(17)	126(1)	C(13)-C(14)-Yb(1)	74.7(7)
C(15)-C(14)-Yb(1)	75.0(7)	C(17)-C(14)-Yb(1)	121(1)
C(11)-C(15)-C(14)	106(1)	C(11)-C(15)-C(16)	127(1)
C(14)-C(15)-C(16)	126(1)	C(11)-C(15)-Yb(1)	74.6(7)
C(14)-C(15)-Yb(1)	73.9(7)	C(16)-C(15)-Yb(1)	122.4(8)
C(15)-C(16)-H(16)	109.5	C(15)-C(16)-H(17)	109.5
H(16)-C(16)-H(17)	109.5	C(15)-C(16)-H(18)	109.5
H(16)-C(16)-H(18)	109.5	H(17)-C(16)-H(18)	109.5
C(14)-C(17)-H(19)	109.5	C(14)-C(17)-H(20)	109.5
H(19)-C(17)-H(20)	109.5	C(14)-C(17)-H(21)	109.5
H(19)-C(17)-H(21)	109.5	H(20)-C(17)-H(21)	109.5
C(13)-C(18)-H(22)	109.5	C(13)-C(18)-H(23)	109.5
H(22)-C(18)-H(23)	109.5	C(13)-C(18)-H(24)	109.5
H(22)-C(18)-H(24)	109.5	H(23)-C(18)-H(24)	109.5
C(12)-C(19)-H(25)	109.5	C(12)-C(19)-H(26)	109.5
H(25)-C(19)-H(26)	109.5	C(12)-C(19)-H(27)	109.5
H(25)-C(19)-H(27)	109.5	H(26)-C(19)-H(27)	109.5
C(11)-C(20)-H(28)	109.5	C(11)-C(20)-H(29)	109.5
H(28)-C(20)-H(29)	109.5	C(11)-C(20)-H(30)	109.5
H(28)-C(20)-H(30)	109.5	H(29)-C(20)-H(30)	109.5
N(1)-C(21)-C(27)	126(1)	N(1)-C(21)-C(22)	116(1)
C(27)-C(21)-C(22)	117(1)	N(2)-C(22)-C(26)	123(1)
N(2)-C(22)-C(21)	118(1)	C(26)-C(22)-C(21)	119(1)
N(2)-C(23)-C(24)	119(1)	N(2)-C(23)-H(66)	120.4
C(24)-C(23)-H(66)	120.4	C(25)-C(24)-C(23)	122(1)
C(25)-C(24)-H(65)	118.8	C(23)-C(24)-H(65)	118.8
C(24)-C(25)-C(26)	118(1)	C(24)-C(25)-H(64)	120.8
C(26)-C(25)-H(64)	120.8	N(3)-C(26)-C(22)	121(1)
N(3)-C(26)-C(25)	122(1)	C(22)-C(26)-C(25)	117(1)
N(4)-C(27)-C(21)	123(1)	N(4)-C(27)-C(28)	122(1)
C(21)-C(27)-C(28)	116(1)	C(29)-C(28)-C(27)	118(1)
C(29)-C(28)-H(33)	121.0	C(27)-C(28)-H(33)	121.0
C(28)-C(29)-C(30)	122(1)	C(28)-C(29)-H(32)	119.0

Table S12. Bond distances (Å) and Angle (deg) for **6** (Continued).

C(30)-C(29)-H(32)	119.0	N(1)-C(30)-C(29)	121(1)
N(1)-C(30)-H(31)	119.5	C(29)-C(30)-H(31)	119.5
C(32)-C(31)-C(35)	107(1)	C(32)-C(31)-C(40)	125(1)
C(35)-C(31)-C(40)	128(1)	C(32)-C(31)-Yb(2)	74.1(7)
C(35)-C(31)-Yb(2)	74.0(7)	C(40)-C(31)-Yb(2)	121(1)
C(31)-C(32)-C(33)	109(1)	C(31)-C(32)-C(39)	124(1)
C(33)-C(32)-C(39)	127(1)	C(31)-C(32)-Yb(2)	74.5(7)
C(33)-C(32)-Yb(2)	74.2(7)	C(39)-C(32)-Yb(2)	118.2(7)
C(34)-C(33)-C(32)	107(1)	C(34)-C(33)-C(38)	128(1)
C(32)-C(33)-C(38)	125(1)	C(34)-C(33)-Yb(2)	73.6(8)
C(32)-C(33)-Yb(2)	74.0(7)	C(38)-C(33)-Yb(2)	120(1)
C(33)-C(34)-C(35)	109(1)	C(33)-C(34)-C(37)	123(2)
C(35)-C(34)-C(37)	127(2)	C(33)-C(34)-Yb(2)	74.9(8)
C(35)-C(34)-Yb(2)	74.8(7)	C(37)-C(34)-Yb(2)	126(1)
C(34)-C(35)-C(31)	109(1)	C(34)-C(35)-C(36)	126(2)
C(31)-C(35)-C(36)	125(2)	C(34)-C(35)-Yb(2)	73.7(7)
C(31)-C(35)-Yb(2)	74.4(7)	C(36)-C(35)-Yb(2)	125(1)
C(35)-C(36)-H(34)	109.5	C(35)-C(36)-H(35)	109.5
H(34)-C(36)-H(35)	109.5	C(35)-C(36)-H(36)	109.5
H(34)-C(36)-H(36)	109.5	H(35)-C(36)-H(36)	109.5
C(34)-C(37)-H(37)	109.5	C(34)-C(37)-H(38)	109.5
H(37)-C(37)-H(38)	109.5	C(34)-C(37)-H(39)	109.5
H(37)-C(37)-H(39)	109.5	H(38)-C(37)-H(39)	109.5
C(33)-C(38)-H(40)	109.5	C(33)-C(38)-H(41)	109.5
H(40)-C(38)-H(41)	109.5	C(33)-C(38)-H(42)	109.5
H(40)-C(38)-H(42)	109.5	H(41)-C(38)-H(42)	109.5
C(32)-C(39)-H(43)	109.5	C(32)-C(39)-H(44)	109.5
H(43)-C(39)-H(44)	109.5	C(32)-C(39)-H(45)	109.5
H(43)-C(39)-H(45)	109.5	H(44)-C(39)-H(45)	109.5
C(31)-C(40)-H(46)	109.5	C(31)-C(40)-H(47)	109.5
H(46)-C(40)-H(47)	109.5	C(31)-C(40)-H(48)	109.5
H(46)-C(40)-H(48)	109.5	H(47)-C(40)-H(48)	109.5
C(42)-C(41)-C(45)	109(2)	C(42)-C(41)-C(50)	130(2)
C(45)-C(41)-C(50)	121(2)	C(42)-C(41)-Yb(2)	75(1)
C(45)-C(41)-Yb(2)	73.8(8)	C(50)-C(41)-Yb(2)	118(1)
C(41)-C(42)-C(43)	110(2)	C(41)-C(42)-C(49)	121(2)
C(43)-C(42)-C(49)	128(2)	C(41)-C(42)-Yb(2)	75(1)
C(43)-C(42)-Yb(2)	75(1)	C(49)-C(42)-Yb(2)	123(1)
C(42)-C(43)-C(44)	108(2)	C(42)-C(43)-C(48)	125(2)
C(44)-C(43)-C(48)	126(2)	C(42)-C(43)-Yb(2)	74(1)
C(44)-C(43)-Yb(2)	72.9(8)	C(48)-C(43)-Yb(2)	124(1)
C(45)-C(44)-C(43)	107(1)	C(45)-C(44)-C(47)	127(2)
C(43)-C(44)-C(47)	125(2)	C(45)-C(44)-Yb(2)	74.5(8)
C(43)-C(44)-Yb(2)	76(1)	C(47)-C(44)-Yb(2)	119(1)
C(44)-C(45)-C(41)	106(1)	C(44)-C(45)-C(46)	126(2)
C(41)-C(45)-C(46)	128(2)	C(44)-C(45)-Yb(2)	73.9(8)
C(41)-C(45)-Yb(2)	73.9(7)	C(46)-C(45)-Yb(2)	121(1)
C(45)-C(46)-H(49)	109.5	C(45)-C(46)-H(50)	109.5
H(49)-C(46)-H(50)	109.5	C(45)-C(46)-H(51)	109.5
H(49)-C(46)-H(51)	109.5	H(50)-C(46)-H(51)	109.5
C(44)-C(47)-H(52)	109.5	C(44)-C(47)-H(53)	109.5
H(52)-C(47)-H(53)	109.5	C(44)-C(47)-H(54)	109.5
H(52)-C(47)-H(54)	109.5	H(53)-C(47)-H(54)	109.5
C(43)-C(48)-H(55)	109.5	C(43)-C(48)-H(56)	109.5
H(55)-C(48)-H(56)	109.5	C(43)-C(48)-H(57)	109.5
H(55)-C(48)-H(57)	109.5	H(56)-C(48)-H(57)	109.5
C(42)-C(49)-H(58)	109.5	C(42)-C(49)-H(59)	109.5
H(58)-C(49)-H(59)	109.5	C(42)-C(49)-H(60)	109.5
H(58)-C(49)-H(60)	109.5	H(59)-C(49)-H(60)	109.5
C(41)-C(50)-H(61)	109.5	C(41)-C(50)-H(62)	109.5
H(61)-C(50)-H(62)	109.5	C(41)-C(50)-H(63)	109.5
H(61)-C(50)-H(63)	109.5	H(62)-C(50)-H(63)	109.5
Pt(1)-C(51)-Yb(1)	81.7(4)	Pt(1)-C(51)-H(51A)	109.5
Yb(1)-C(51)-H(51A)	57.9	Pt(1)-C(51)-H(51B)	109.5
Yb(1)-C(51)-H(51B)	72.9	H(51A)-C(51)-H(51B)	109.5
Pt(1)-C(51)-H(51C)	109.5	Yb(1)-C(51)-H(51C)	166.2
H(51A)-C(51)-H(51C)	109.5	H(51B)-C(51)-H(51C)	109.5
Pt(1)-C(52)-Yb(1)	80.5(4)	Pt(1)-C(52)-H(52A)	109.5
Yb(1)-C(52)-H(52A)	62.0	Pt(1)-C(52)-H(52B)	109.5
Yb(1)-C(52)-H(52B)	69.7	H(52A)-C(52)-H(52B)	109.5
Pt(1)-C(52)-H(52C)	109.5	Yb(1)-C(52)-H(52C)	169.1
H(52A)-C(52)-H(52C)	109.5	H(52B)-C(52)-H(52C)	109.5
